# *Geotrichum candidum* gene expression and metabolite accumulation inside the cells reflect the strain oxidative stress sensitivity and ability to produce flavour compounds

**DOI:** 10.1093/femsyr/foy111

**Published:** 2018-10-05

**Authors:** P Pracharova, P Lieben, B Pollet, J M Beckerich, P Bonnarme, S Landaud, D Swennen

**Affiliations:** UMR GMPA, AgroParisTech, INRA, Université Paris-Saclay, 78850 Thiverval-Grignon, France

**Keywords:** cheese, yeast, flavour metabolism, methionine catabolism, oxidative stress, metabolome

## Abstract

*Geotrichum candidum* is a fungus-like yeast widely used as a starter culture for cheese ripening for its proteolytic and lipolytic activities and its contribution to the cheese flavours. The sequenced strain *G. candidum* CLIB 918 was isolated from cheese Pont-L’Evêque. This strain's ability to produce volatile compounds was compared to the ability of a known strong sulphur compound producer *G. candidum* strain (Gc203). The aminotransferase-coding genes *BAT2* and *ARO8* were identified to be involved in methionine catabolism. The production of volatile compounds indicated that the sequenced strain was a moderate producer compared to the strong producer strain. The major volatile compounds were produced from sulphur amino acid, branched-chain amino acid and fatty acid metabolisms. Metabolite content of the cells showed that the ability of the strain to produce volatile compounds was inversely proportional to its ability to store amino acids inside the cells. Reduced glutathione, hypotaurine and taurine intracellular concentrations and volatile fatty aldehyde production indicated the role of oxidative stress sensitivity in flavour production. The increase in expression of several genes in a Reblochon-type cheese at the end of ripening confirmed that oxygen and iron were key factors regulating cheese flavour production.

## Teaser

The ability of *Geotrichum candidum* to produce volatile compounds is strain dependent and inversely proportional to its ability to store metabolites inside the cells

## INTRODUCTION

*Geotrichum candidum*, a fungus-like yeast, is found in soil, plants, insects or raw milk and consequently in raw milk cheese. It has long been used as a starter culture for cheese ripening for its proteolytic and lipolytic activities and its contribution to the cheese flavours (Boutrou and Guéguen 2005). Cheese ripening involves complex biochemical, biophysical and microbiological transformations. The process begins by milk curdling after adding lactic bacteria and rennet enzymes. The substrates present in the resulting acidic curd are mainly proteins and peptides, triglycerides and free fatty acids, lactose and lactate, and citrate. The action of yeasts, moulds and ripening bacteria, which can grow sequentially in the curd, results in the production of aroma compounds. These aroma compounds are produced through secondary metabolism when there is an unbalance in the central metabolism due to unavailability of substrates, cofactors, oxygen or metals. Amino acids can be catabolised in aldehydes, alcohols, carboxylic acids and sulphur compounds; free fatty acids contribute to the production of ester, S-methyl thioesters, methyl-ketones or secondary alcohols (Marilley 2004). Unsaturated fatty acids can be hydroperoxidised and produce aldehydes.

Due to their low perception threshold, volatile sulphur compounds are responsible for the typical flavour of the fermented product. These compounds arise primarily from the degradation by the microorganisms of the sulphur/carbon bond of methionine producing methanethiol, a common precursor of volatile sulphur compounds (Landaud, Helinck and Bonnarme 2008). Nevertheless, these technological abilities are variable amongst the *G. candidum* strains isolated from cheese (Guichard and Bonnarme 2005) and raise a question about the origin of metabolic differences, which is a practical question for cheese makers and starter producers. To address these issues, we have compared the *G. candidum* sequenced strain's ability (CLIB918 (Gc918) isolated from cheese Pont-L’Evêque; Morel *et al.*^2015^) to produce volatile compounds to the ability of a known *G. candidum* strong sulphur compound producer, the Gc203 strain (Guichard and Bonnarme 2005). In order to control the environmental parameters, a liquid medium was formulated using the main substrates encountered in cheese. This choice was also necessary in order to follow intracellular metabolites and to compare the strains’ metabolism. The expression of aminotransferase-coding genes was measured to identify which genes were involved in methionine catabolism. The Gc918 gene expression at the end of ripening in a Reblochon-type cheese was analysed to correlate the results obtained in synthetic condition with those obtained in real conditions.

## MATERIALS AND METHODS

### Strains and culture conditions

The *Geotrichum candidum* strain CLIB918/ATCC204307 was obtained from the French collection CIRM-Levures (https://www6.inra.fr/cirm/Levures) and the Gc203 strain from the UCMA collection of University of Caen, France (Guichard and Bonnarme 2005). The analysis of the intracellular metabolite content is not possible in cheese because the cells cannot be separated from the curd. To understand the strain variability, the *G. candidum* strains were cultivated in a synthetic medium. The strains were grown in a 500-mL flask with 100 mL of minimal medium enriched or not with methionine (Sigma Aldrich, Steinheim, germany) 6 g/L at 25°C with orbital agitation (180 rpm). The minimal medium was composed of Difco Yeast Nitrogen Base without amino acids and ammonium sulphate (Becton Dickinson and Company, Sparks, Maryland, USA) with sodium lactate (BDH Chemicals, London, UK) 15 g/L and proline (Sigma Aldrich, Steinheim, Germany) 1 g/L. Three cultures were made for each condition, and after 3 days of culture, samples were collected for analyses. A culture volume of 10 mL was centrifuged (15 min 4000 rpm, room temperature), the cells were frozen in liquid nitrogen and kept at –80°C until RNA analyses. A volume of 10 mL was collected in glass flasks and kept at –80°C for Gas Chromatography-Mass Spectrometry (GC-MS) analyses. A volume of 40 mL was collected for Ultra High Pressure liquid Chromatography-High Relsolution-Mass Spectrometry (UHPLC-HR-MS) analyses.

#### Volatile compounds extraction and analyses

The volatile compounds were analysed by a dynamic head space analyser coupled to GC-MS as previously described (Forquin *et al.*^2011^; see GC-MS Table S1, Supporting Information)

#### Extraction and analyses of intracellular metabolites

The extraction of metabolites was adapted from the method previously described (Hebert *et al.*^2013^). Cultures were centrifuged and washed with ultrapure water (quality LC-MS (Liquid Chromatography-Mass Spectrometry), Optima Chemical, Douglas, Georgia, USA) (15 min, 4000 rpm, room temperature), and then the pellets were transferred in a pre-weighted 2-mL tube and centrifuged (5 min, 15 000 rpm room temperature). We weighted precisely 1 g of wet cells that were suspended in 1 mL of formic acid (quality LC-MS, Optima Chemical, Douglas, Georgia, USA) 0.1% and incubated at 95°C for 10 min. After centrifugation (5 min, 15 000 rpm, 4°C), the supernatants were filtrated (nylon membrane, porosity 0.22 μm, diameter 13 mm) and diluted in formic acid 0.1% for analyses quality LC/MS (Optima Fisher Chemical) for analyses by UHPLC-HR-MS (UHPLC Ultimate 3000 and HR-MS-Q EXACTIVE, Thermo Fisher Scientific, Pittsburgh, Pennsylvania, USA). UHPLC conditions were as follows: metabolites were separated on Hypersil Gold phenyl column (length = 150 mm, internal diameter = 2.1 mm, particles size = 3 μm, Thermo Fisher Scientific, Pittsburgh, Pennsylvania, USA). The pressure at the beginning of the gradient was 120 bar, and the column temperature was 25°C. The flow was 0.25 mL/min, and the solvents were D: acetonitrile (quality LC-MS, Optima Chemical, Douglas, Georgia, USA) and B: water (quality LC-MS, Optima Chemical, Douglas, Georgia, USA) with nonafluoropentanoic acid (Sigma Aldrich, Steinheim, Germany) (3 mM). The elution gradient was as follows: 4 min at 98% B + 2% D, then 98% to 2% B in D for 6 min, level at 2% B and 98% D during 3 min. The injection volume was 5 μL, and samples were stored at 7°C in the autosampler before the injection. The duration of one analysis was 14 min.

The detection was performed with a Q-EXACTIVE-Orbitrap mass spectrometer with a Heated Electrospray Ionization probe operated in the positive ionization mode. Full scans were acquired with a scan speed of 3.7 scan/sec and a mass range from 50 to 700 u.m.a. (unified atomic mass unit) with a high resolution of 70 000.

Data were identified and quantified (ng/g wet weight) using TraceFinder software according to the calibration solution (Calibration table: see Calibration Table, Supporting Information; see LC-MS Table, Supporting Information)

### Statistical analysis

The data of volatile compounds and intracellular metabolites obtained from three independent cultures were graphically represented using the R software version 3.3.4 (https://cran.r-project.org/, https://www.rstudio.com/; see Boxplots, Supporting Information).

### Aminotransferase-coding genes expression

RNA extraction, DNase treatment and reverse transcription: The –80°C cell pellets were melted with 1 mL TRIzol reagent (Invitrogen, Cergy Pontoise, France) and broken with 0.5-mm diameter zirconium beads (Biospec products, Bartlesville, OK) in a BeadBeater (Precellys, Saint Quentin en Yvelynes, France) using two 20-s mixing sequences at a speed of 6500 rpm. RNA extraction, DNase treatment and reverse transcription were done following protocols previously described (Monnet, Back and Irlinger 2012).

Real-time PCR and data analyses: Oligonucleotide primers were designed using LightCycler probe design software (v1.0; Roche Applied Science, Mannheim, Germany) and synthesised by Eurogentec (Seraing, Belgium) (primers list, see Primers Table, Supporting Information). The PCR and the data analyses were done as previously described (Monnet, Back and Irlinger 2012).

### Metabolic pathways

The map of sulphur metabolism pathways was adapted from Hebert *et al.* (2013). The metabolic maps were built and *G. candidum* homologous genes were identified using information from these websites: http://bioinformatics.psb.ugent.be/orcae/overview/Geca; https://www.yeastgenome.org/; http://gryc.inra.fr/; https://metacyc.org/; http://www.kegg.jp/kegg/; https://www.ebi.ac.uk/chebi/ and https://blast.ncbi.nlm.nih.gov/Blast.cgi.

### Gene expression in Reblochon-type cheese

Gc918 strain was previously used for Reblochon-type cheese manufacturing in semi-industrial conditions (Castellote *et al.*^2015^), and RNA were extracted and sequenced (Monnet *et al.*^2016^). The *G. candidum* genes playing a role in the production of volatile compounds during metabolism were selected, and their expression at the end of ripening was analysed to validate the hypothesis in real conditions (see Reblochon Table, Supporting Information).

## RESULTS AND DISCUSSION

### Production of Intracellular metabolites and volatile compounds

**Figure 1.**
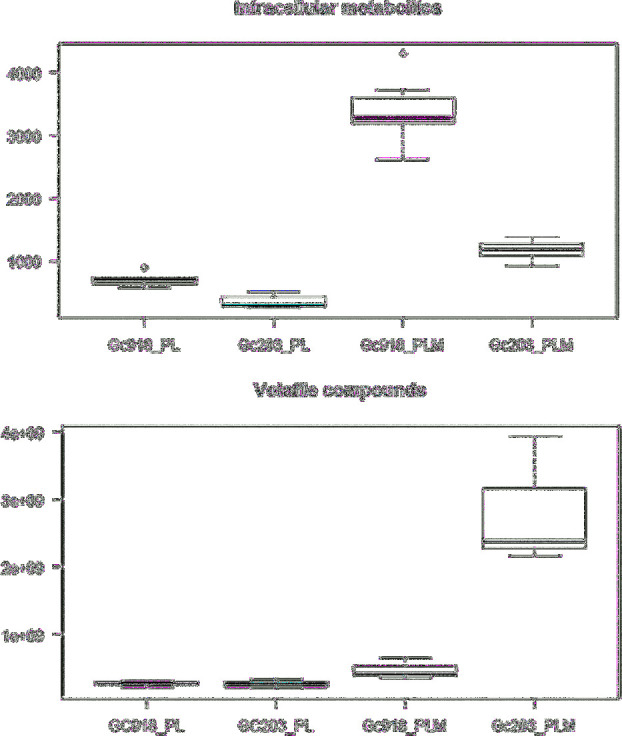
Production of intracellular metabolites and volatile compounds. The quantified intracellular metabolites (y-axis unit: ng/g wet weight) and volatile compounds (y-axis unit: area/g dry weight/L) are represented as boxplots for each strain (x-axis) on both media. PL: proline–lactate, PLM: proline–lactate–methionine.

**Figure 2.**
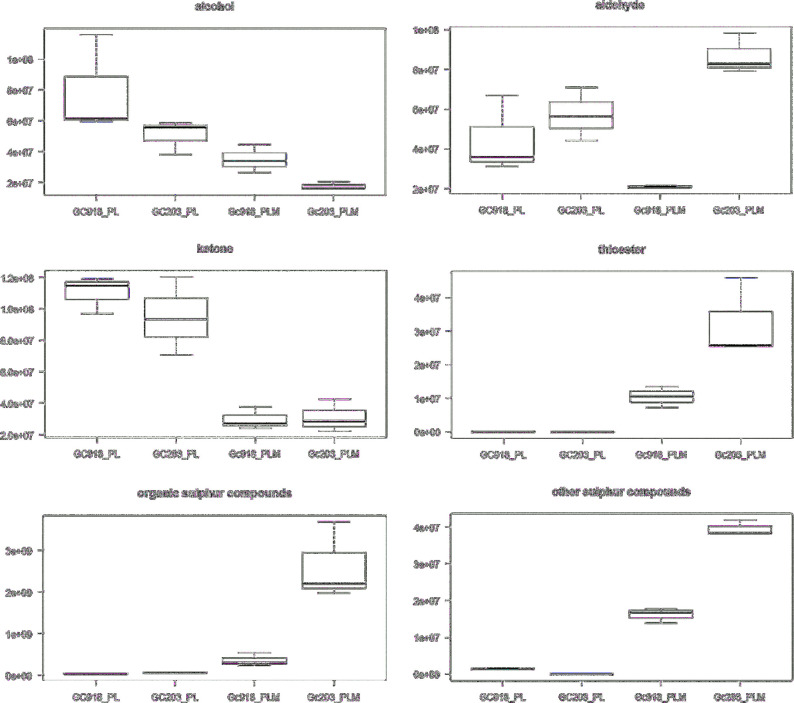
Production of volatile compound classes. Classes of volatile compounds (y-axis unit: area/g dry weight/L) are represented as boxplots for each strain (x-axis) on both media. PL: proline–lactate, PLM: proline–lactate–methionine.

Cells were collected after 3 days of culture during the stationary phase to simulate what could be the condition at the end of cheese ripening when the availability of nutrients is lowered on the cheese surface. The growth parameters and biomass production of both strains were similar in our conditions (data not shown). Volatile compounds (underlined in the next sections) and intracellular metabolites produced by Gc918 and Gc203 after 3 days in minimal medium enriched or not with methionine were separated and analysed (boxplots representation for each molecule presented, see Boxplots, Supporting Information). Both strains had the same behaviour with an increase in metabolites and volatile compounds when methionine was added to the medium, with the highest increase in volatile compounds for Gc203. However, the level of metabolites in the cells and the volatile compounds produced seemed inversely abundant depending on the strain as shown in Fig. 1. As indicated in Fig. 2, both strains produced approximately the same level of volatile compounds on a minimal medium. When methionine was added, organic sulphur compounds, thioesters and other compounds derived from methionine catabolism were more highly produced by Gc203 than by Gc918, ketone production being the same for both strains. Interestingly, Gc918 had a slightly higher production of alcohol versus aldehyde when aldehyde production of Gc203 was five times higher than alcohol production in methionine-enriched medium. The equilibrium between aldehyde and alcohol is linked to the equilibrium between the alcohol dehydrogenase cofactors, NADH and NAD^+^, which could reflect the redox state of the cells and modulate the production of volatile compounds (Bloem *et al.*^2016^). NADH pool is used for the oxidative phosphorylation to produce energy. NAD^+^ can be a co-substrate of sirtuins and play a role in lifespan extension (Lin and Guarente 2003). Oxygen diffusion inside the cell could be strain dependent and inside the ecosystem's surface could be subject to a gradient of concentration as observed in cheese matrix (Tammam *et al.*^2001^). The oxygen availability results in production of various aldehydes or alcohols depending on the availability of NADH cofactors and could have a role in the final flavour production. This effect of oxygen is well known in fermented products where oxygen is almost absent (wine, beer, bread) but has not yet been described on aerobic processes such as cheese ripening.

**Figure 3.**
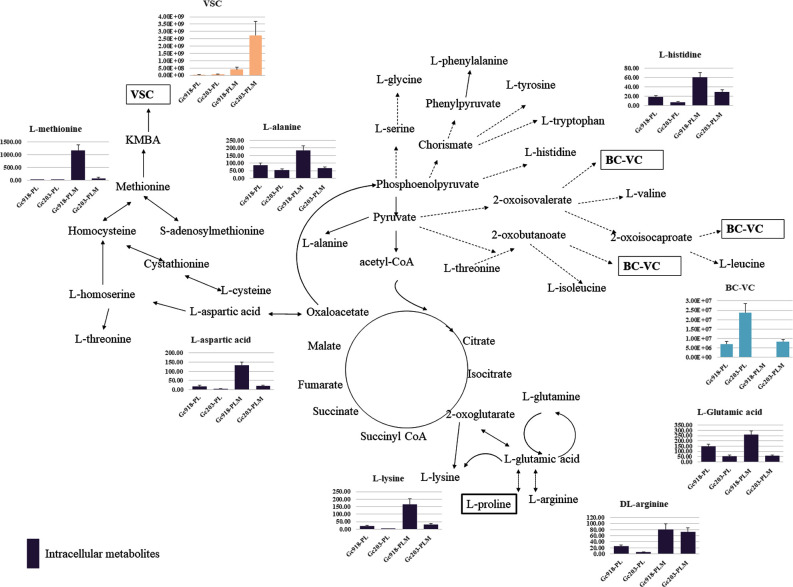
Metabolism of volatile compounds. Volatile sulphur compounds (VSC, y-axis unit: area/g dry weight/L) and branched-chain volatile compounds (BC-VC, y-axis unit: area/g dry weight/L) are represented for both strains (x-axis) on both media. PL: proline–lactate, PLM: proline–lactate–methionine, and parts of central carbon metabolism involved in the production of these compounds are depicted. The most abundant amino acids (y-axis ng/g wet weight) are presented. Dashed arrows indicate that several reactions are involved.

In our experimental conditions, the carbon and nitrogen sources for biosynthesis were provided by proline and methionine when added. The map indicating the metabolism producing volatile sulphur compounds and branched-chain volatile compounds from proline and methionine is depicted in Fig. 3, and the most abundant intracellular amino acids are shown. Methionine, glutamic acid, lysine, aspartic acid, alanine and histidine were highly stored in Gc918, especially in the medium enriched in methionine contrary to Gc203. Only arginine was equally abundant in both strains when methionine was added to the medium.

### Methionine metabolism

**Figure 4.**
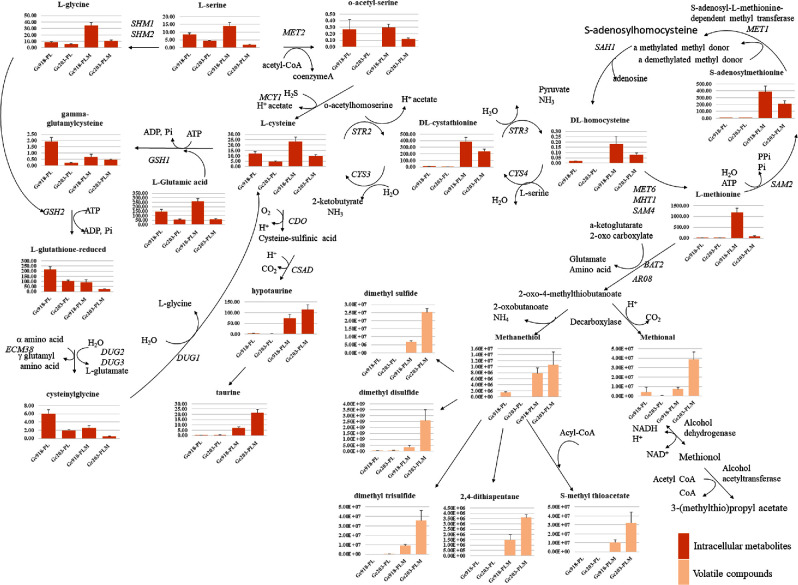
Metabolism of sulphur amino acids. Volatile sulphur compounds (VSC, y-axis area/g dry weight/L) and metabolites (y-axis unit: ng/g wet weight) produced from sulphur metabolism are presented for each strain (x-axis) in both media (PL: proline–lactate, PLM: proline–lactate–methionine). The standard names of the *Saccharomyces cerevisiae* genes involved in these pathways are indicated and homologous *G. candidum* genes are listed in Reblochon Table, Supporting Information. Biosynthesis of taurine is absent in *S. cerevisiae*. CDO: cysteine dioxygenase; CSAD: cysteine sulphinic acid decarboxylase.

An increase in volatile sulphur compounds was observed when methionine was added to the culture medium, especially for Gc203 (Fig. 4). Dimethyl disulphide (DMDS), 3-(methylthio)-propanal (methional), dimethyl trisulphide (DMTS), S-methyl thioacetate, dimethyl sulphide (DMS) and methanethiol were the most abundant molecules. The degradation of methionine in *Geotrichum candidum* involves two steps as in other yeasts (Arfi, Landaud and Bonnarme 2006), the first step resulting from the action of a non-specific transaminase giving 2-oxo-4-methylthiobutanoate (KMBA), which is subsequently converted to methanethiol or methional. DMDS, DMTS, DMS and 2,4-dithiapentane molecules result from a chemical reaction involving methanethiol and depend on the oxidation state of the cells (Landaud, Helinck and Bonnarme 2008). When the detected metabolites involved in sulphur metabolism accumulated in the cells, their respective concentrations were higher in Gc918 than in Gc203, except for taurine and hypotaurine. The high concentration of methionine added to the medium probably induced an oxidative stress, especially for Gc203 that seemed more sensitive to this stress as indicated by the lower level of reduced glutathione in Gc203 than in Gc918. Glutathione is important to prevent damages resulting from oxidation. This sensitivity is also reflected by the levels of hypotaurine and taurine, which could have a protective role against oxidative stress (Lambert *et al.*^2015^; Bin, Huang and Zhou 2017).

To confirm the role of stress linked to a high concentration of methionine (enzymatic hydrolysis of caseins) at the end of ripening, the metatranscriptome of a cheese ecosystem, including Gc918 strain, was monitored in a Reblochon-type cheese. These results (see Reblochon Table, Supporting Information) showed that the expression of genes involved in the methyl cycle and in the synthesis of cysteine was enhanced at the end of ripening. The methyl cycle produces S-adenosyl-methionine, which is the major methyl donor for trans-methylation reactions. S-adenosyl-methionine is involved in cell cycle regulation and a high level of S-adenosyl-methionine could be a signal for the arrest of cell cycle (Mizunuma *et al.*^2004^). Cysteine is involved in the synthesis of glutathione, and the expression enhancement of genes involved in its synthesis could reflect a need for glutathione. The expression of the *DUG3* gene coding one protein of a complex involved in the degradation of glutathione was also enhanced at the end of ripening, which could be a way to recycle cysteine. Cysteine is also a component of iron–sulphur clusters, which are cofactors of numerous essential enzymes such as enzymes responsible of cellular respiration. The enhancement of sulphur metabolism gene expression could result in methionine production at the end of ripening and reflect the involvement of *G. candidum* in flavour compounds produced from methionine catabolism. The oxygen availability and the oxidative stress sensitivity of the strain have a major role in the catabolism of methionine producing the cheesy, cabbage or garlic aromas (Sablé and Cottenceau 1999) typical of surface-ripened cheeses.

### Branched-chain amino acids metabolism

**Figure 5.**
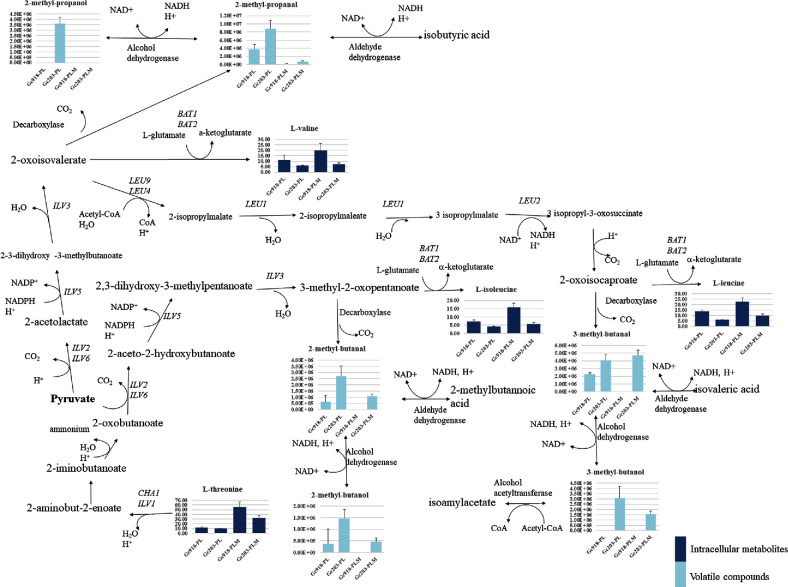
Branched-chain amino acids metabolism. Branched-chain volatile compounds (BC-VC, y-axis unit: area/g dry weight/L) and amino acids (y-axis unit: ng/g wet weight) are presented for each strain (x-axis) in both media (PL: proline–lactate, PLM: proline–lactate–methionine). The standard names of the *Saccharomyces cerevisiae* genes involved in these pathways are indicated and homologous *G. candidum* genes are listed in Reblochon Table, Supporting Information.

In our synthetic media, the yeasts have to biosynthesise their amino acids (except proline and methionine when added). In biosynthesis pathways (Fig. 5), branched-chain amino acids can be converted to volatile compounds. The metabolite 3-methyl-2-oxopentanoate can produce *L*-isoleucine by transamination or 2-methyl-butanal by decarboxylation, the latter being possibly reduced to 2-methyl-butanol by the action of an alcohol dehydrogenase with NADH and H^+^ consumption and NAD^+^ production. The transamination of 2-oxo-isovalerate leads to the production of *L*-valine and its decarboxylation gives 2-methyl-propanal and subsequently 2-methyl-propanol, whereas 2-oxo-isovalerate leads to the production of 2-oxo-isocaproate where transamination produces *L*-leucine and decarboxylation produces 3-methyl-butanal and 3-methyl-butanol. In the medium with proline as the only carbon and nitrogen source, the greatest amount of volatile compounds produced by Gc918 was aldehydes, especially 2-methyl-propanal and 3-methyl-butanal. The addition of methionine decreased the production of these molecules. For Gc203, 2-methyl-propanal was approximately twice as abundant as other molecules in the medium with proline only. When methionine was added, 3-methyl-butanal was then the most produced by Gc203. The level of branched-chain amino acids accumulated in Gc918 was higher than in Gc203 in which the level of amino acids remained nearly the same after addition of methionine. As observed for accumulated sulphur metabolites, a higher level was observed for Gc918 metabolites than for Gc203, and conversely volatile compounds were most produced by Gc203.

The genes involved in branched-chain amino acid metabolism were selected and their expression was analysed in a Reblochon-type cheese (see Reblochon Table, Supporting Information). Expression of genes involved in the synthesis of leucine was enhanced at the end of ripening and especially *LEU1* coding isopropylmalate isomerase, which needs iron for its activity. At the end of ripening, iron starvation could inactivate Leu1p leading to the accumulation of 2-isopropylmalate, which can be an enhancer of the expression of genes coding leucine biosynthesis proteins. Iron is an important element regulating the development of the aerobic surface ecosystem (Monnet *et al.*^2015^). Iron is complexed to various components such as proteins, carbohydrate or citrate (Vegarud, Langsrud and Svenning 2000). Milk lactoferrin is a mammalian protein belonging to the transferrin family able to bind two ferric ions. The high stability constant of the iron–protein complex confers to lactoferrin an anti-microbial activity (Ward, Paz and Conneely 2005), leading to low iron availability for microorganisms in the curd. Iron scarcity is involved in the inhibition of a great number of enzymes that use iron as cofactor (e.g. isopropylmalate isomerase (*LEU1*)), resulting in metabolite accumulation and central metabolism unbalance leading to volatile compounds production. In the Reblochon-type cheese, genes potentially involved in iron uptake, when they are regulated, are induced at day 14 or day 19; only the gene coding the fungi transcription factor induced during iron starvation (*HapX*; Hortschansky *et al.*^2007^) had its expression increased at the end of Reblochon-type cheese ripening.

*BAT2*, the aminotransferase-coding gene involved in the synthesis and degradation of leucine and methionine, as shown in this work (see below), was highly expressed all along the ripening process.

Alcohol dehydrogenases and aldehyde dehydrogenases are involved in the production of volatile compounds. Nine genes coding alcohol dehydrogenases and 10 genes coding aldehyde dehydrogenases were identified but their specificity is unknown. Three genes from each category had their expression increased at the end of Reblochon-type cheese ripening and could be involved in the equilibrium of NADH, H^+^ and NAD^+^ and the production of volatile compounds.

Pyruvate decarboxylase and pyruvate dehydrogenase coding genes were enhanced at the end of Reblochon-type cheese ripening. Pyruvate decarboxylase could be the enzyme involved in the decarboxylation of 2-oxoisovalerate (2-methyl-propanal production), 2-oxoisocaproate (3-methyl-butanal production) and 3-methyl-2-oxopentanoate (2-methyl-butanal production), as well as 2-oxo-4-methylthiobutanoate (methional production). The high enhancement of the expression of pyruvate decarboxylase coding genes could reflect the need to degrade metabolites that accumulate in the cells at the end of the ripening process. Pyruvate dehydrogenase allows the synthesis of acetyl-CoA where accumulation leads to the incorporation of an acetyl in carboxylic acid ester by the action of an alcohol acetyltransferase.

### Fatty acid peroxidation

**Figure 6.**
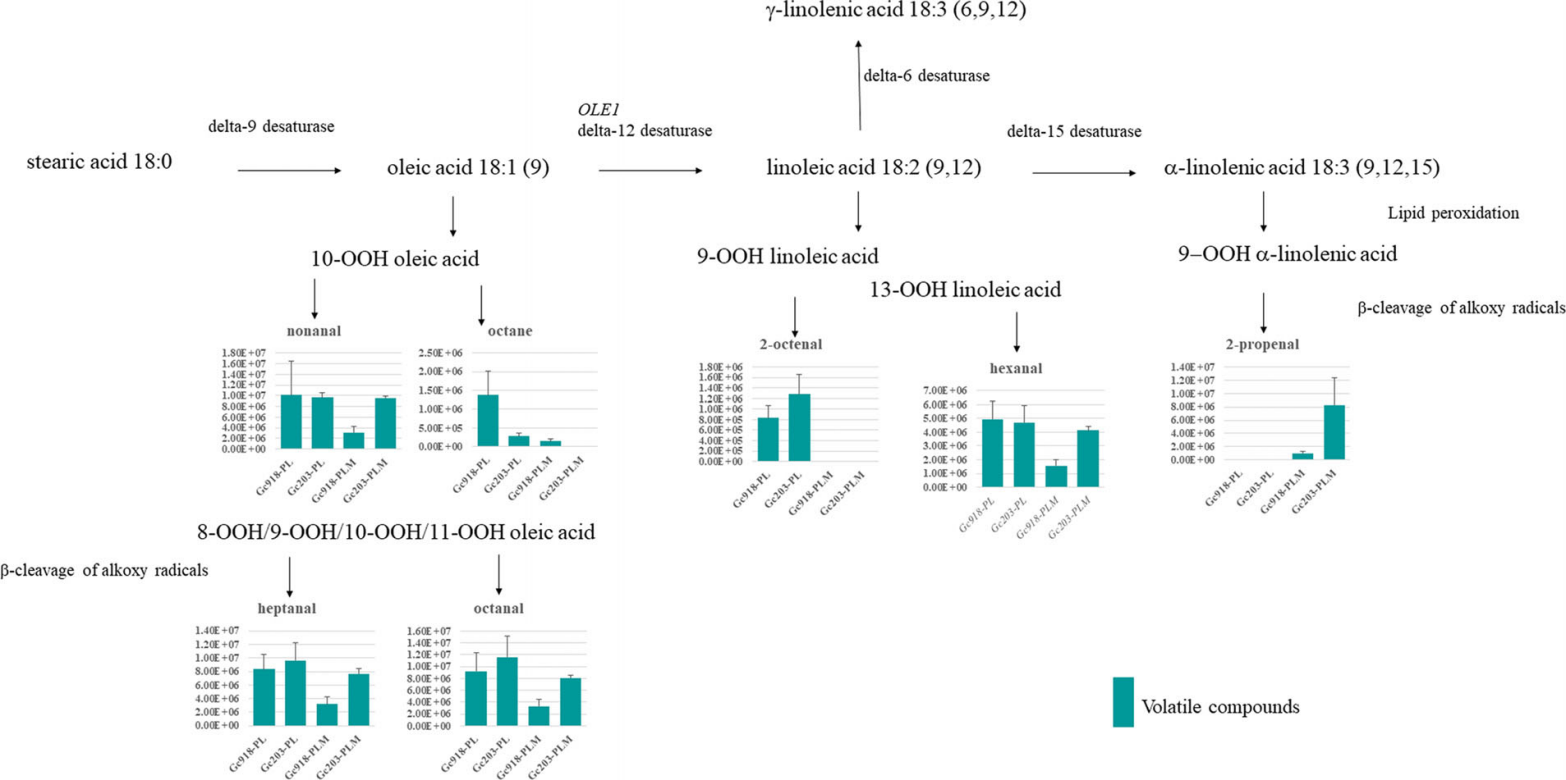
Fatty acid peroxidation. Pathways for the synthesis of unsaturated fatty acids from stearic acid are depicted. Fatty aldehyde (y-axis unit: area/g dry weight/L) produced from β-cleavage of alkoxy radicals of hydroperoxide fatty acid is presented for both strains (x-axis) on both media (PL: proline–lactate, PLM: proline-lactate-methionine). *Geotrichum candidum* genes are listed in Reblochon Table, Supporting Information.

Both strains produced fatty aldehydes and alkane in minimal medium, and Gc203 produced higher levels of these molecules than Gc918 when methionine was added to the medium (Fig. 6). The production of fatty aldehydes and alkane could be the result of hydroperoxidation of unsaturated fatty acids. Nonanal, octane, heptanal and octanal could be produced by β-cleavage of alkoxy radicals from hydroperoxide oleic acid; 2-octenal and hexanal from hydroperoxide linoleic acid (Frankel, Neff and Selke 1981); and 2-propenal from hydroperoxide α-linolenic acid (Wang and Cui 2015). These molecules were indicative of an oxidative stress, and the higher production in Gc203 than in Gc918, when methionine was added, confirmed its higher sensitivity.

Hydroperoxide fatty acid by-products can be degraded by aldoketo-reductase (Penning 2015); two genes coding these enzymes have their expression enhanced at the end of Reblochon-type cheese ripening (see Reblochon Table, Supporting Information). The gene coding hydroperoxide sensor (*HYR1*) and the transcription factor (*YAP1*) coding gene, required for oxidative stress tolerance, were also induced at the end of Reblochon-type cheese ripening as well as the genes coding a delta 9 and delta 12 desaturase indicative of the need to synthesise unsaturated fatty acids for the maintenance of cell membrane. The enhancement of the expression of these genes reflects an oxidative stress for *G. candidum* at the end of ripening, which is also confirmed by the increase of the expression of two genes involved in oxidative stress response (*PRX1* and *SOD1*). Hydroperoxidation of the fatty acids occurs only in the presence of oxygen; its availability is determinant for the production of these fatty aldehydes producing green flavours (Sablé and Cottenceau 1999).

### *Geotrichum candidum* genes involved in methionine catabolism

In *G. candidum*, the major pathway for the initial step to degrade methionine is the transamination pathway involving aminotransferase (Bonnarme *et al.*^2001^). No specific transaminase for methionine was identified in yeasts. In *Kluyveromyces lactis*, two aminotransferase genes (*ARO8-1* and *ARO8-2*) were identified to be involved in methionine transamination (Kagkli *et al.*^2006^), and *ARO8-2* was overexpressed in methionine-enriched medium (Hébert, Casaregola and Beckerich 2011). *Yarrowia lipolytica* aminotransferase *BAT1* gene was overexpressed and *ARO8* gene was repressed in methionine-enriched medium (Hebert *et al.*^2013^). In order to identify which genes were involved in methionine transamination in *G. candidum*, a quantitative PCR was done. In *G. candidum* genome (Morel *et al.*^2015^), four aminotransferase genes were identified, *BAT1* (GECA08s02584g), *BAT2* (GECA01s10493g), *ARO8-27* (GECA27s00967g) and *ARO8-03* (GECA03s05400g). To determine which transaminases were involved in methionine degradation, expression of these genes was compared on the two media studied (Table 1). For both strains, *BAT2* and *ARO8-03* genes showed the strongest expression when methionine was provided. It is therefore highly probable that in *G. candidum*, Bat2p and Aro8-03p are the aminotransferases involved in methionine catabolism.

**Table 1.** Expression of aminotransferase genes.

	BAT1	BAT2	ARO8-03	ARO8-27
Gc918-PL	1 ± 0.05	1 ± 0.28	1 ± 0.35	1 ± 0.04
Gc918-PLM	**1.54 ± 0.15**	**18.37 ± 3.38**	**6.79 ± 1.51**	**1.23 ± 0.12**
Gc203-PL	1 ± 0.13	1 ± 0.53	1 ± 0.12	1 ± 0.17
Gc203-PLM	1.32 ± 0.20	**3.28 ± 0.34**	**2.55 ± 0.30**	0.77 ± 0.38

Expression of aminotransferases genes was compared for both strains on medium enriched with methionine (PLM) using minimal medium (PL) as reference. The values are the means of three independent biological replicates. Values in bold indicate significant differences between gene expression on both media.

## CONCLUSION

Our results indicated that the sequenced strain Gc918 was a moderate producer of aroma compounds compared to the strong producer Gc203. The major volatile compounds were produced from sulphur, branched-chain amino acid and fatty acid metabolisms. Oxygen and iron are two major factors influencing the microorganism flavour metabolism of cheese surface ecosystem. The ability of *Geotrichum candidum* to produce volatile compounds is linked to the redox state of the cells, the oxidative stress sensitivity and is strain dependent and inversely proportional to its ability to store amino acids inside the cells. To determine the ability of volatile compound production with the future goal to have a relevant screening of technological strains, gene expression analyses are not enough and should be complemented by studies measuring the concentration of intracellular metabolites.

## FUNDING

Pavla Pracharova was funded with an ERASMUS grant and an INRA grant.

***Conflict of Interest*.** None declared.

## Supplementary Material

Supplementary Data
