# Supplementary material for: Geotrichum candidum gene expression and metabolite accumulation inside the cells reflect the strain oxidative stress sensitivity and ability to produce flavour compounds
Source: FEMS Yeast Res. 2018 Oct 5;19(1):foy111. doi: 10.1093/femsyr/foy111 (PMC6211236; doi:10.1093/femsyr/foy111)

## Slide 1
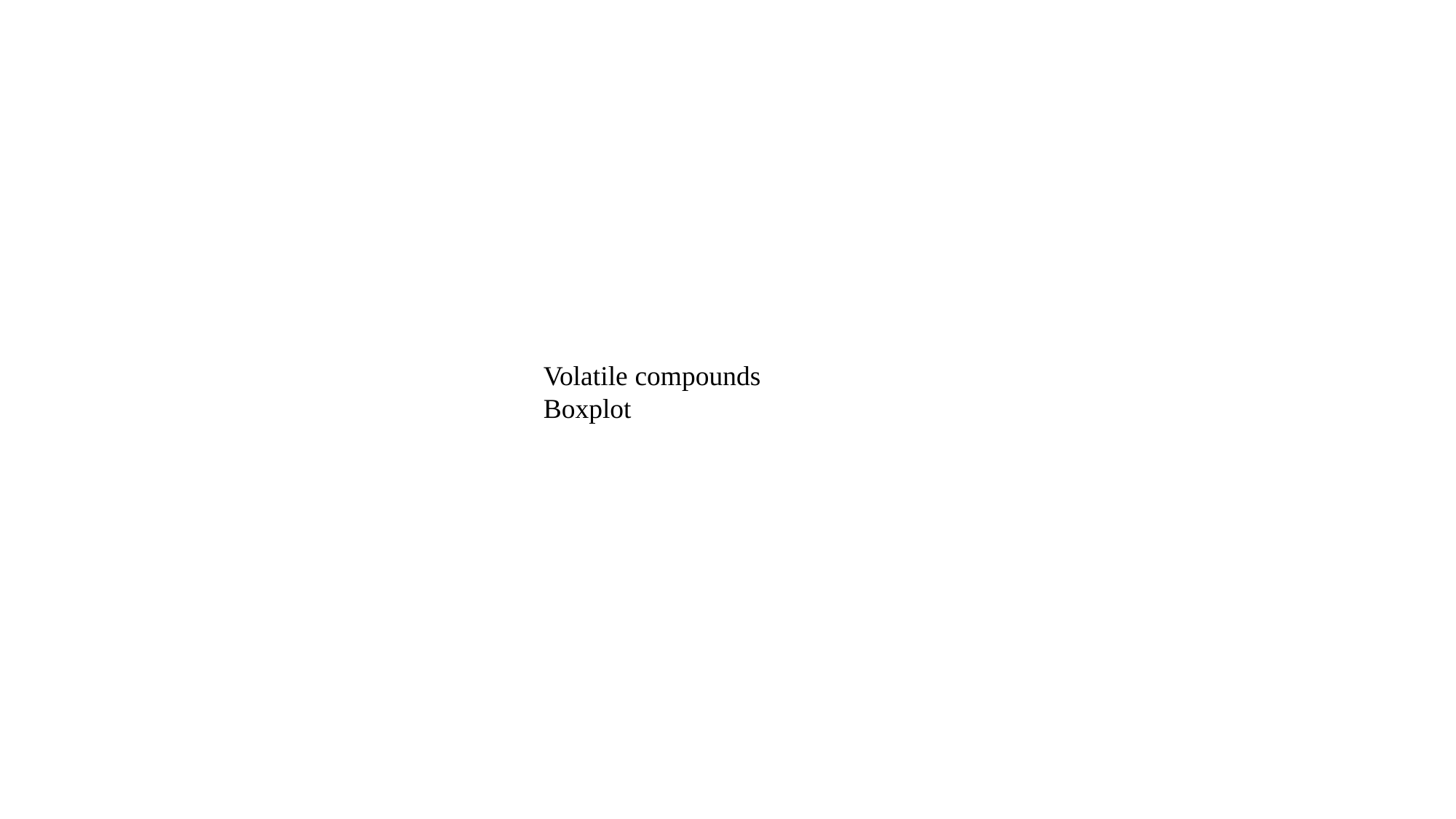

Volatile compounds
Boxplot

## Slide 2
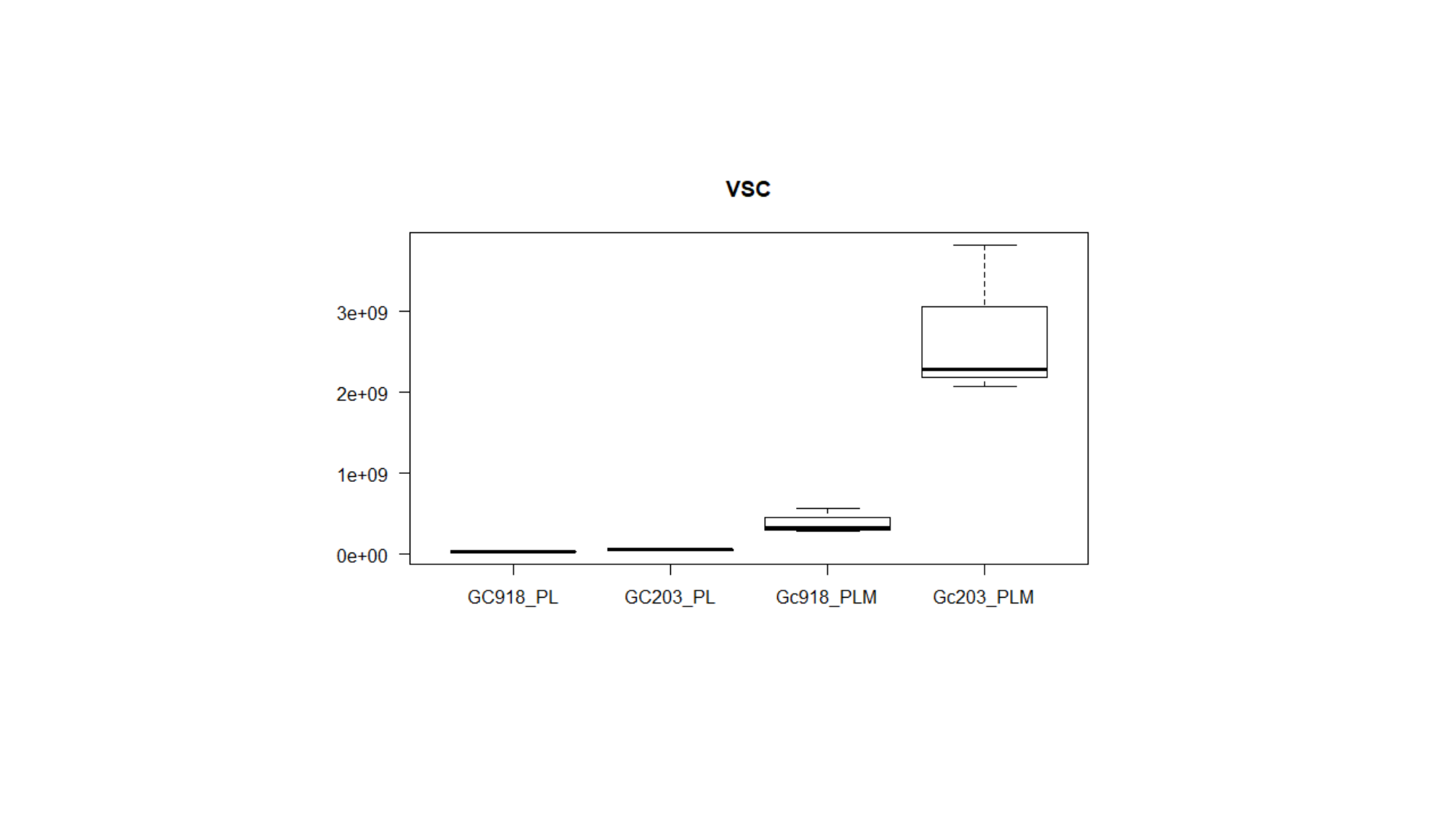

## Slide 3
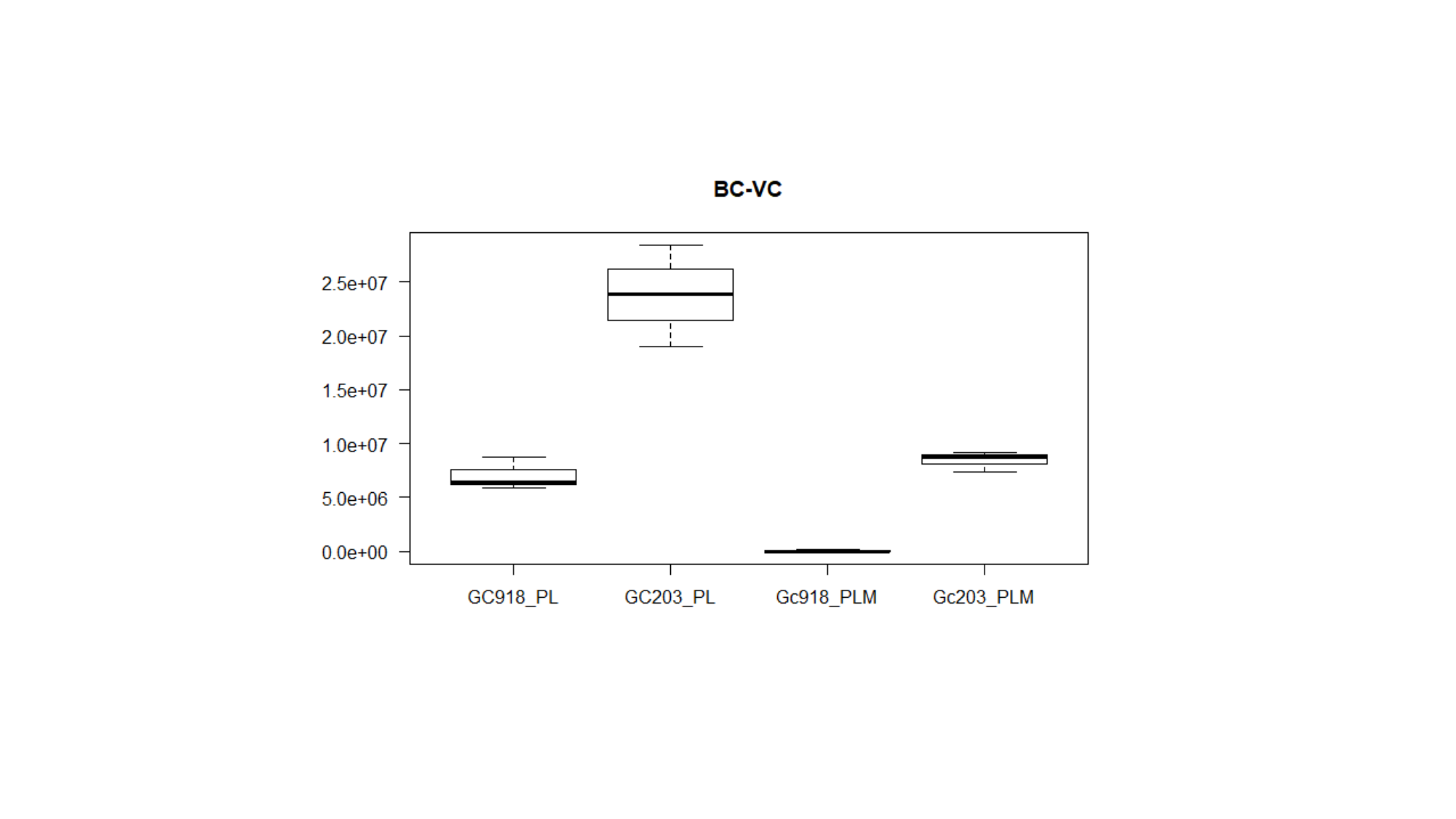

## Slide 4
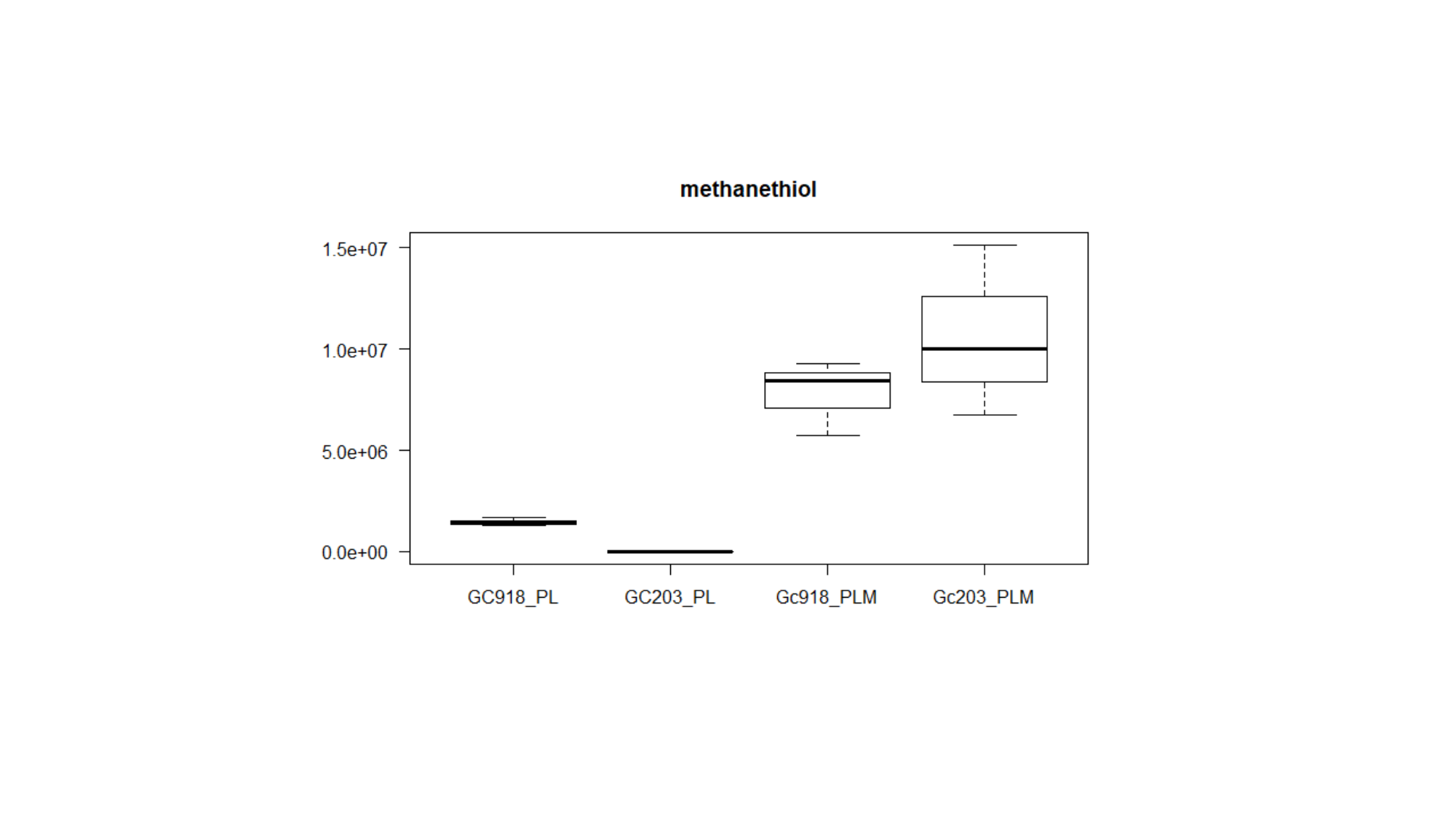

## Slide 5
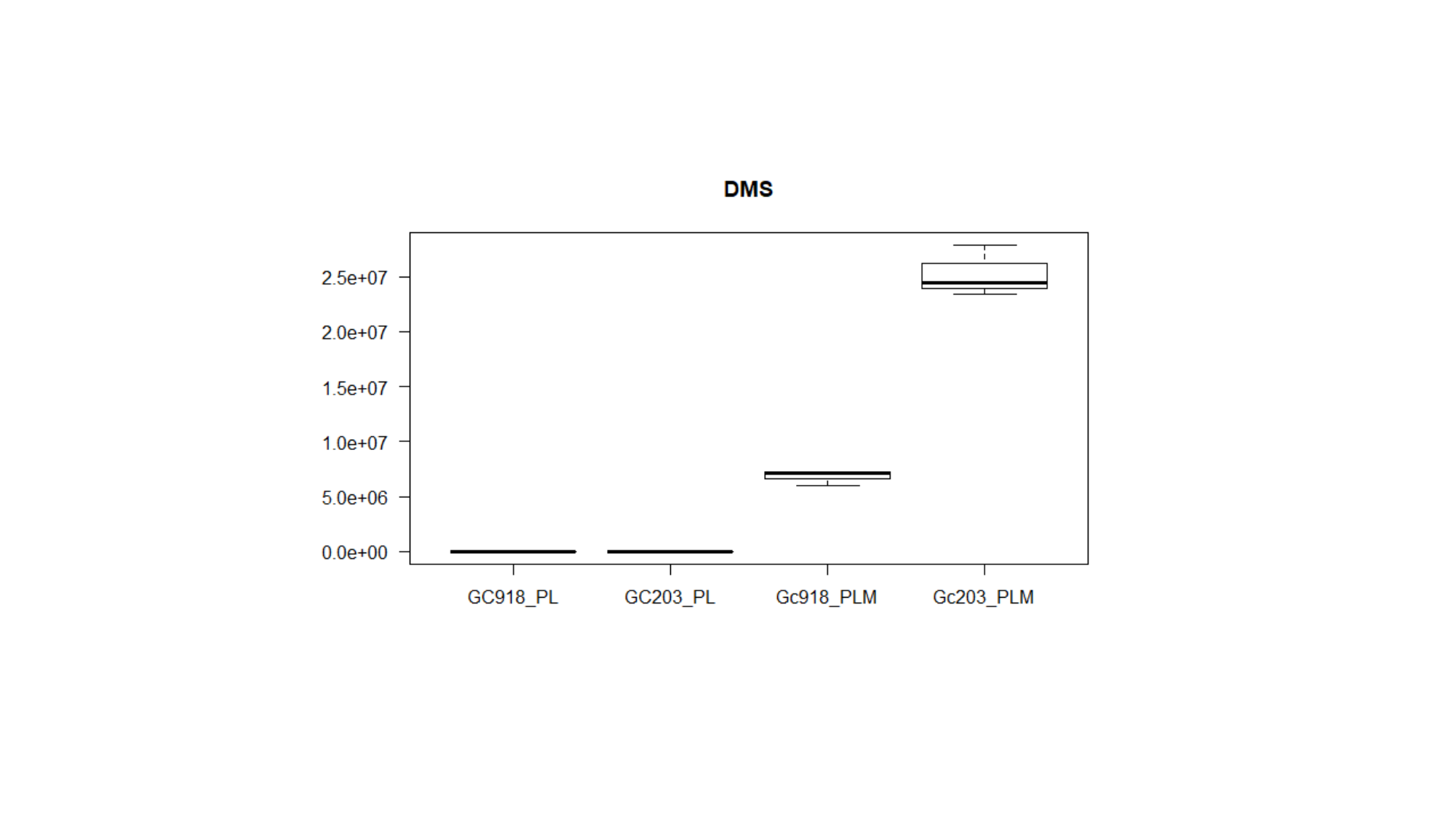

## Slide 6
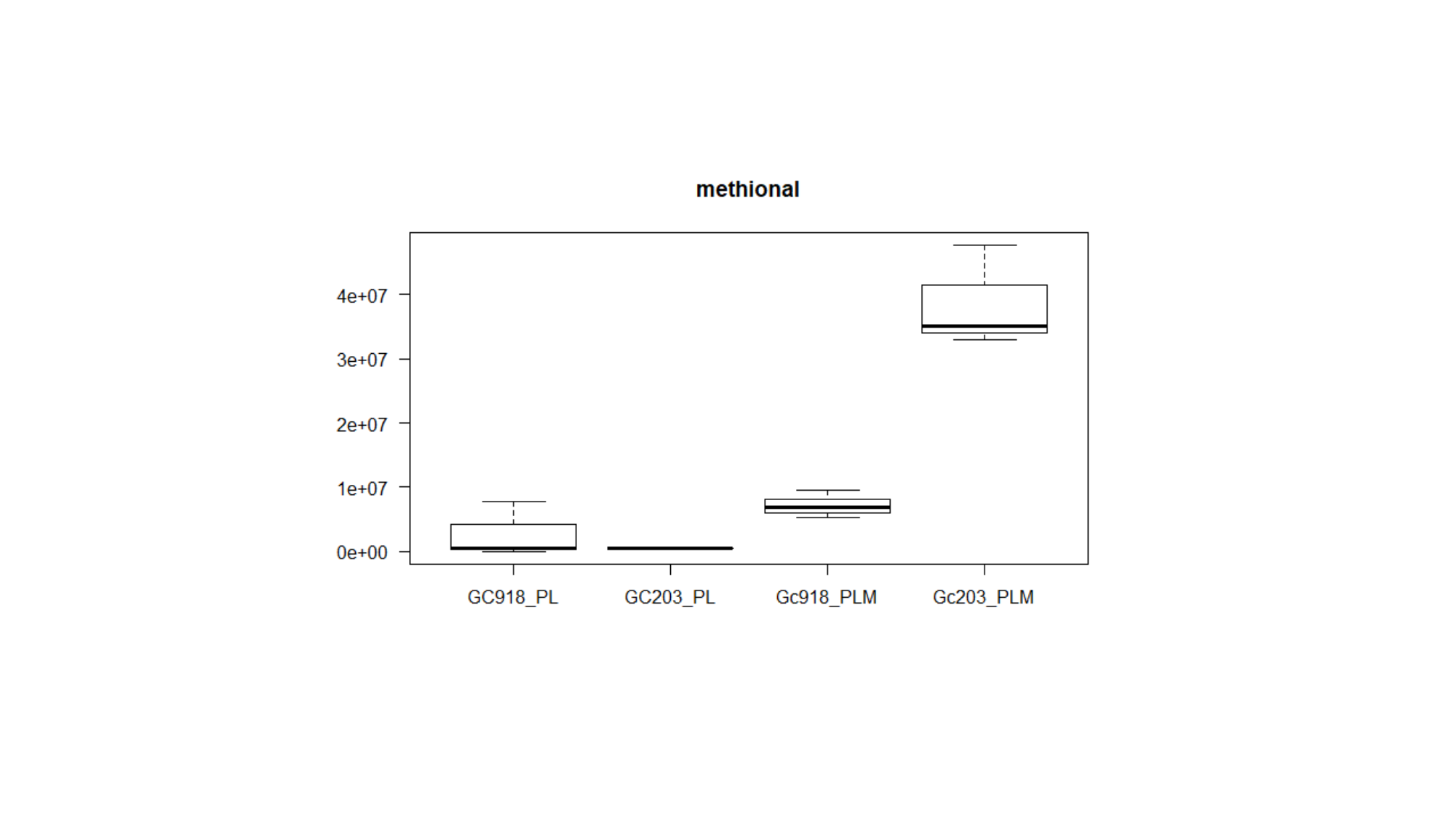

## Slide 7
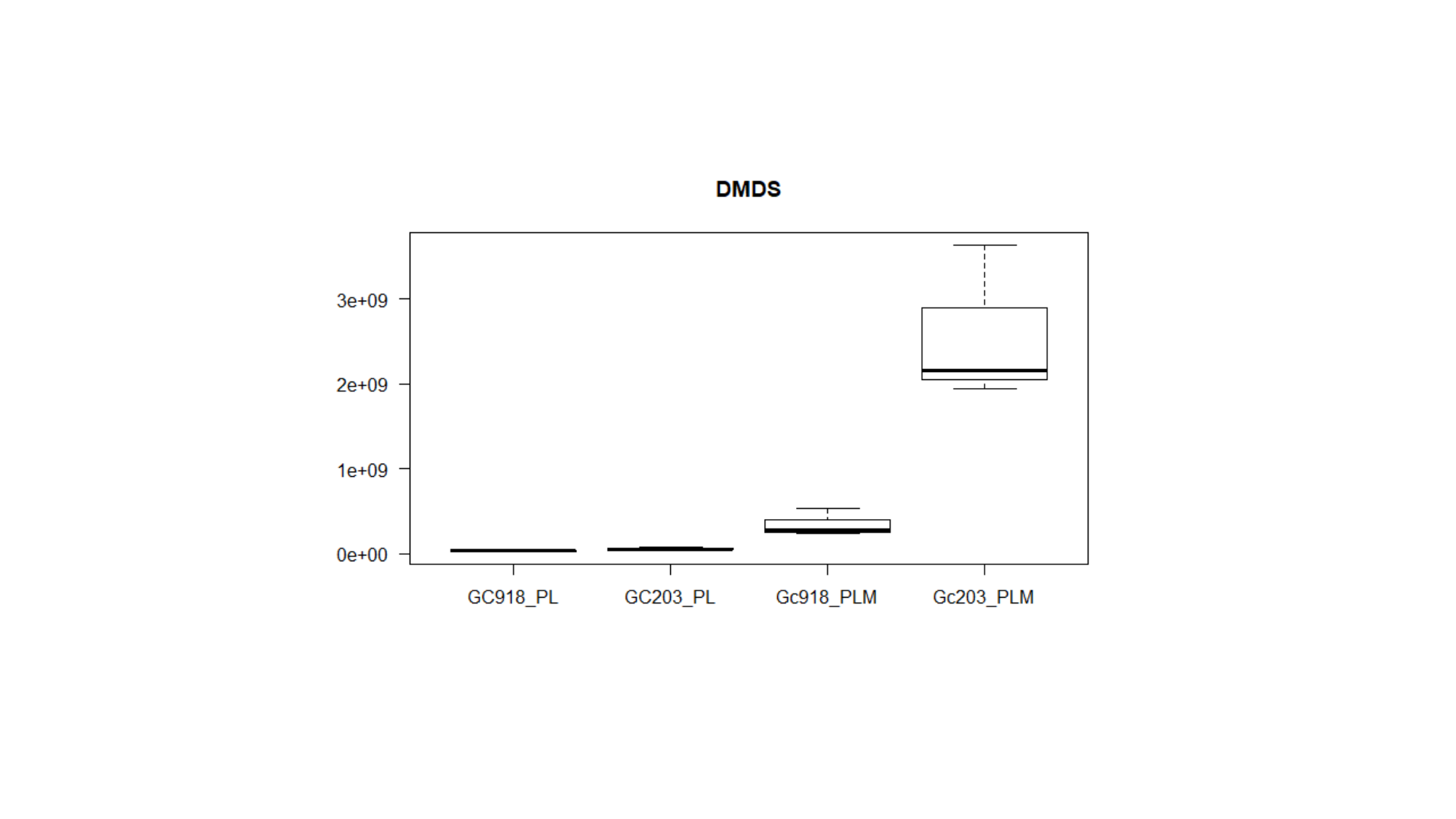

## Slide 8
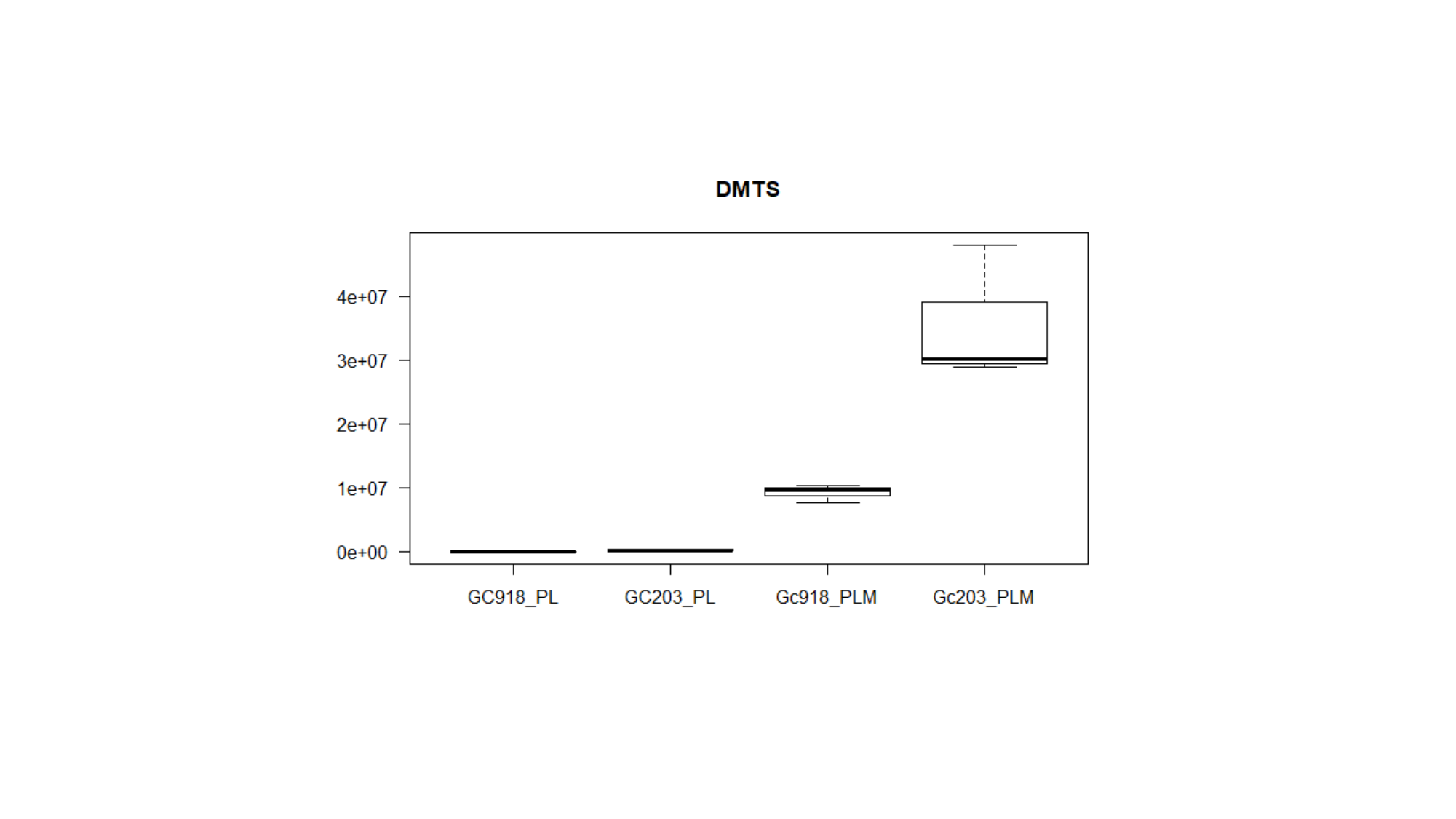

## Slide 9
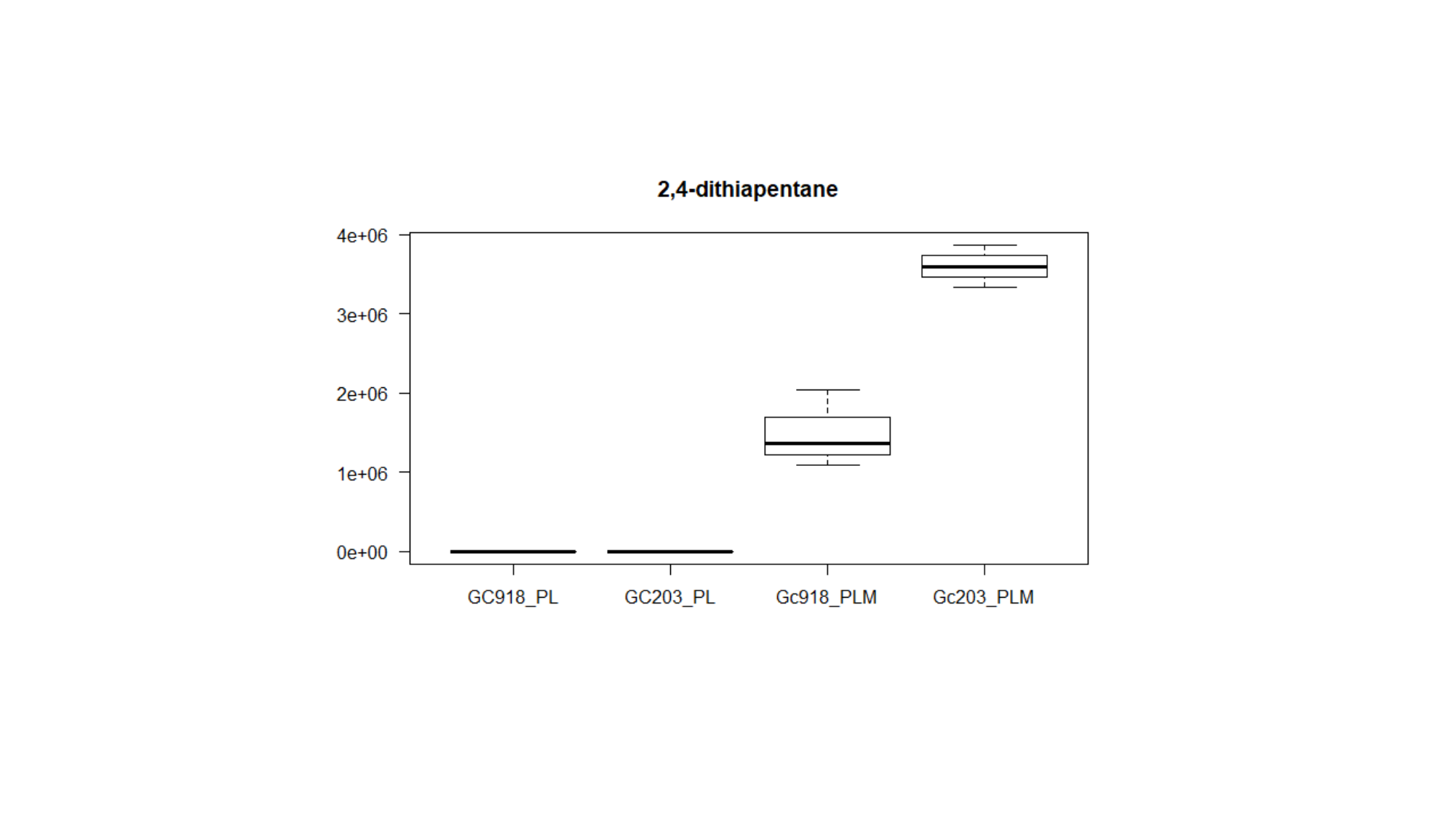

## Slide 10
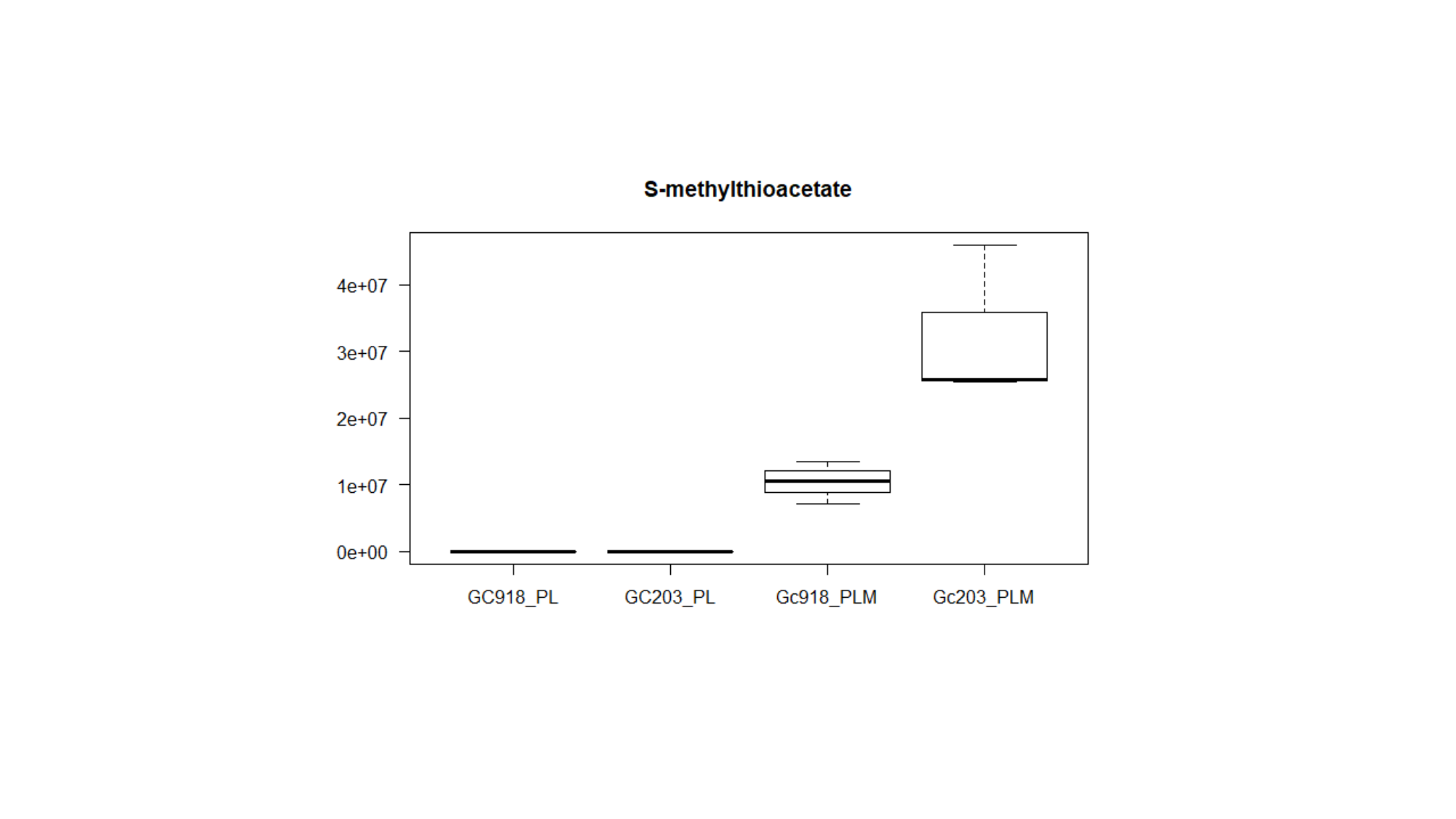

## Slide 11
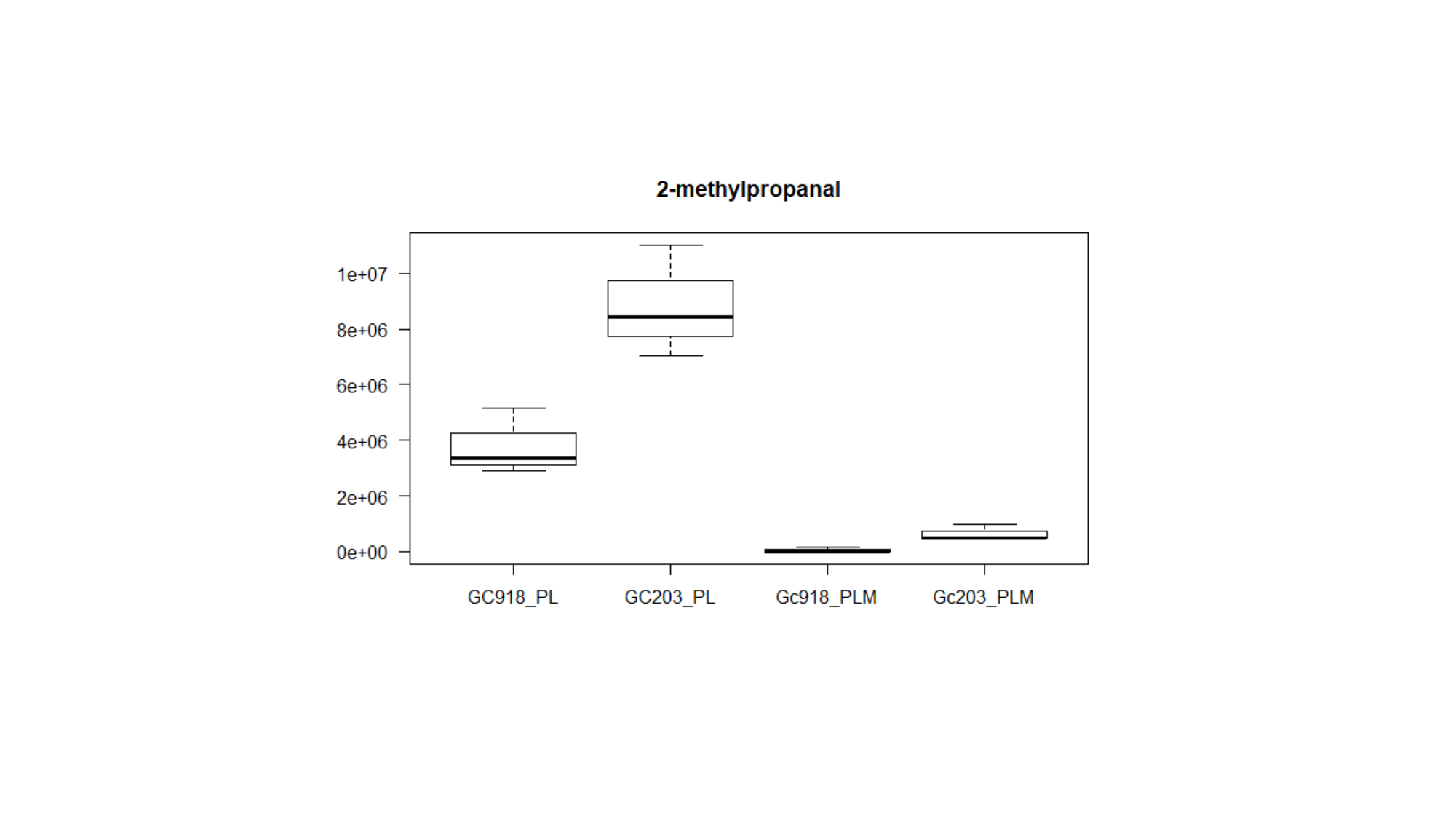

## Slide 12
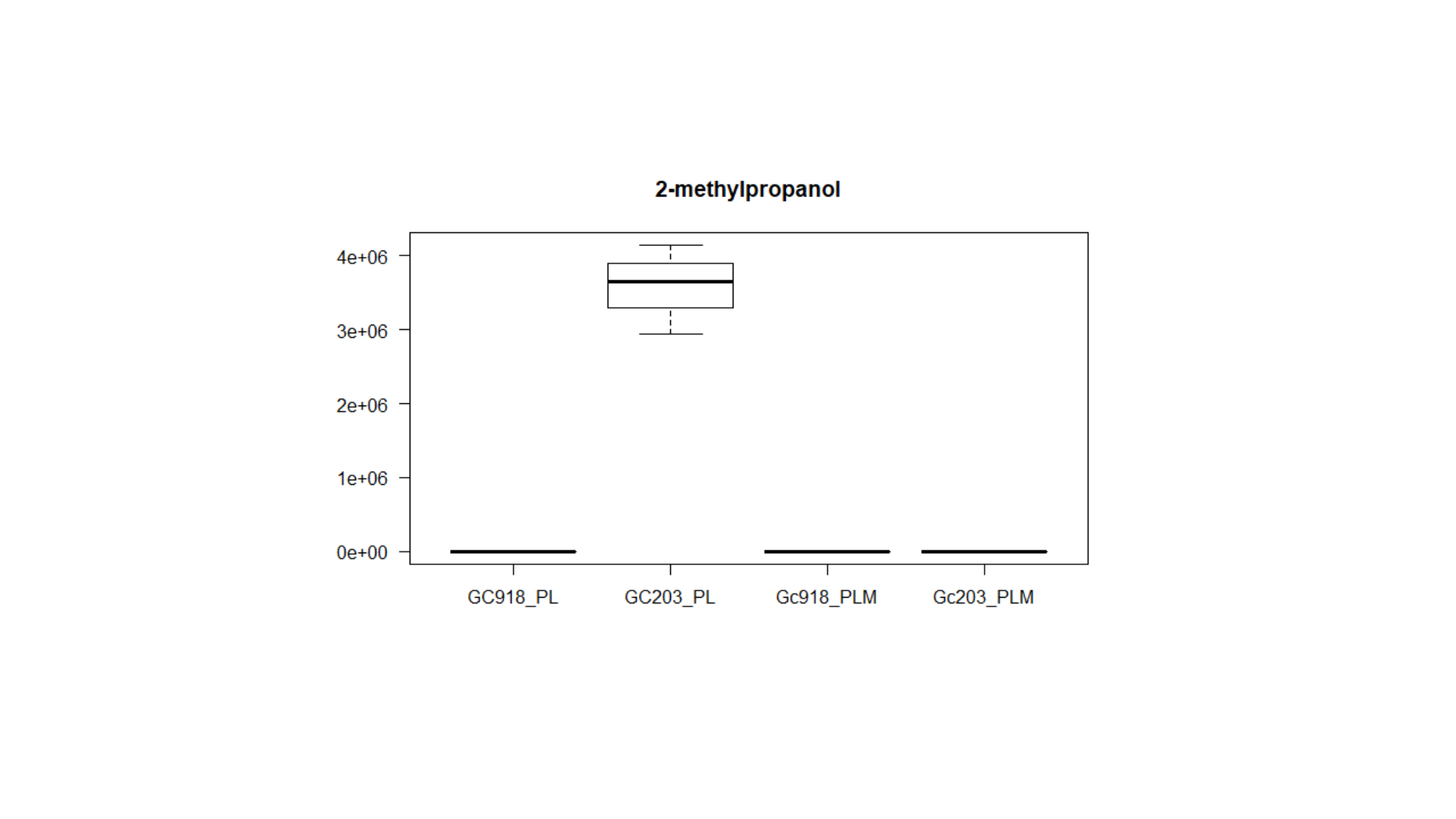

## Slide 13
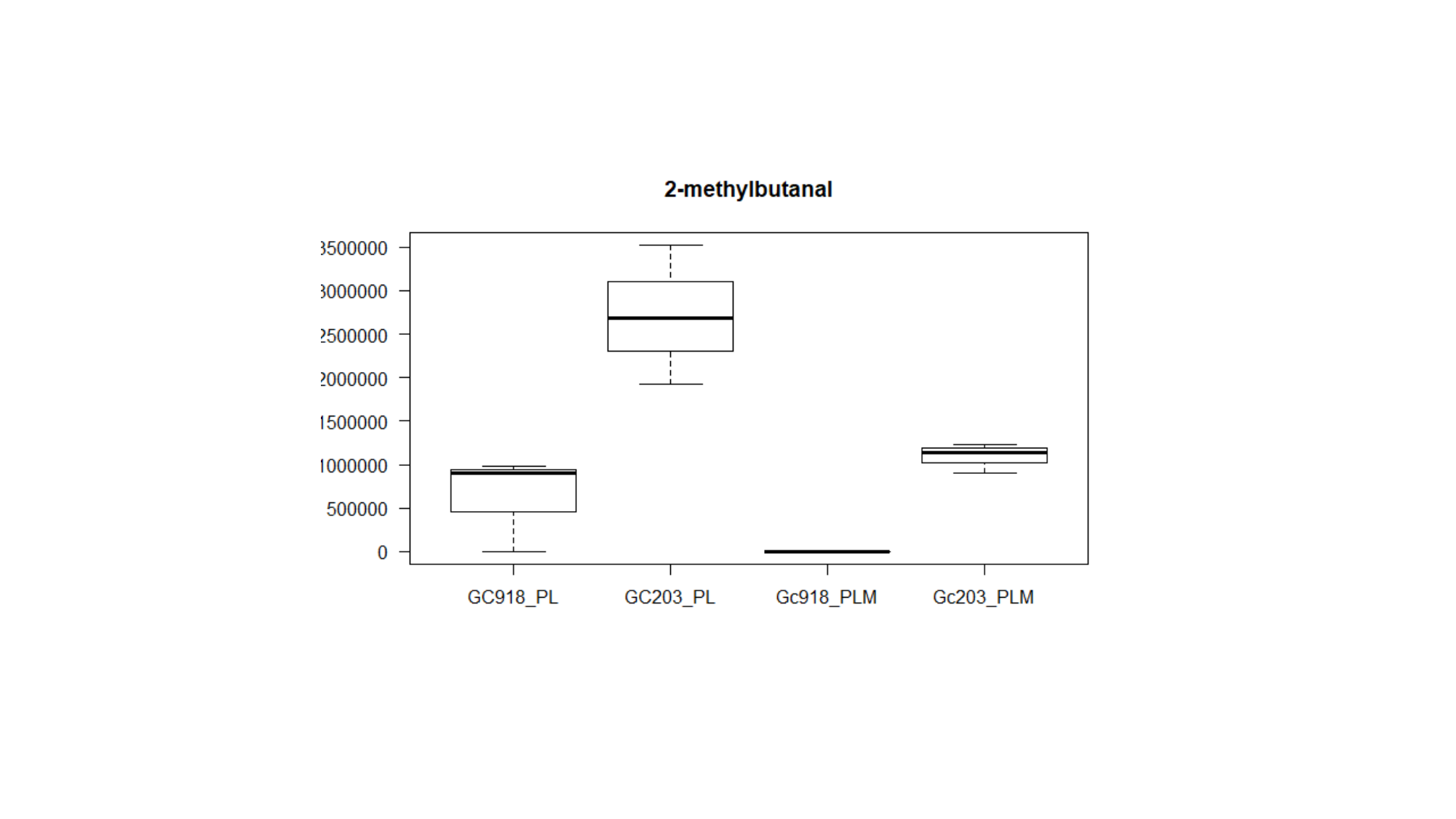

## Slide 14
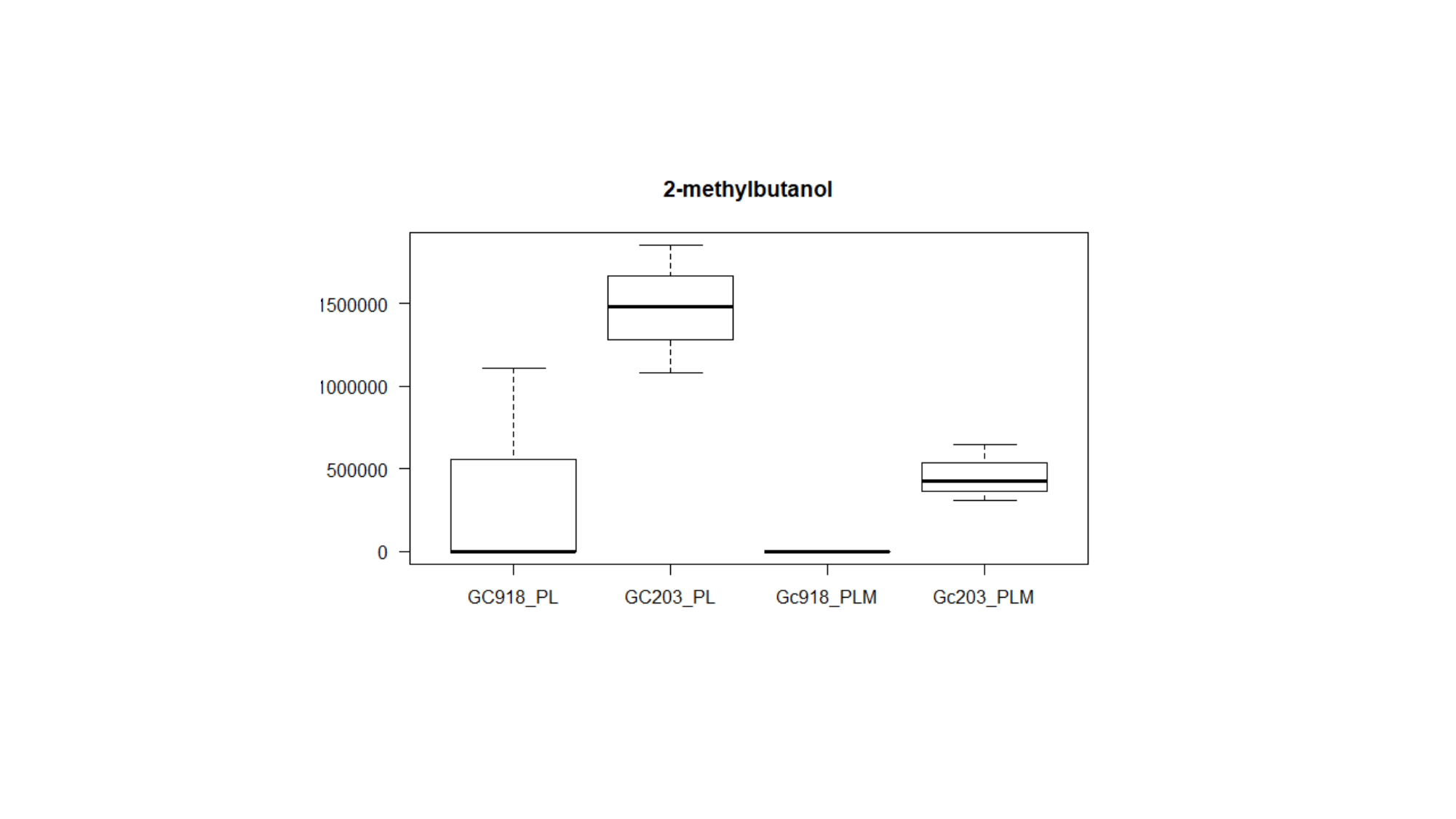

## Slide 15
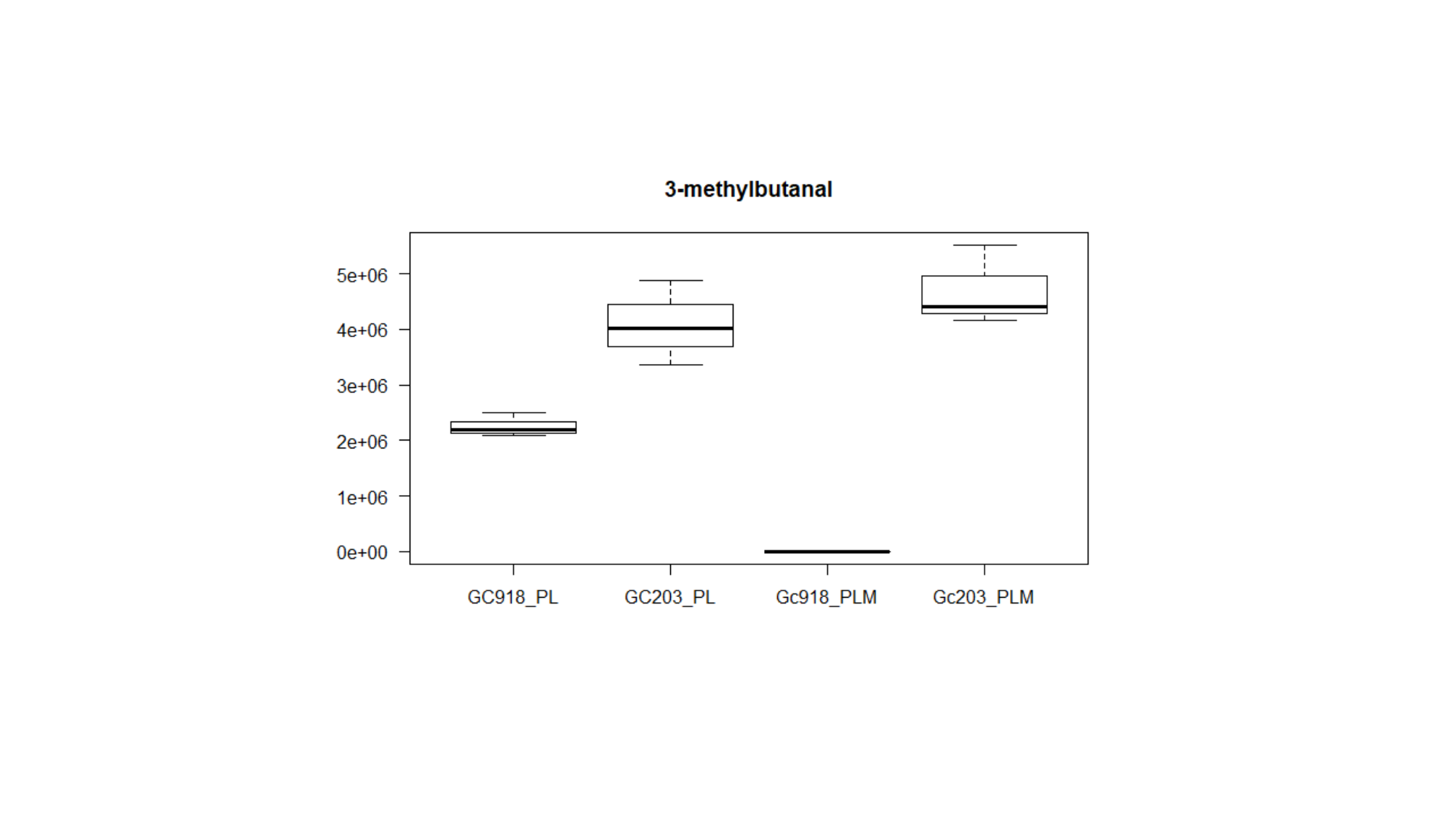

## Slide 16
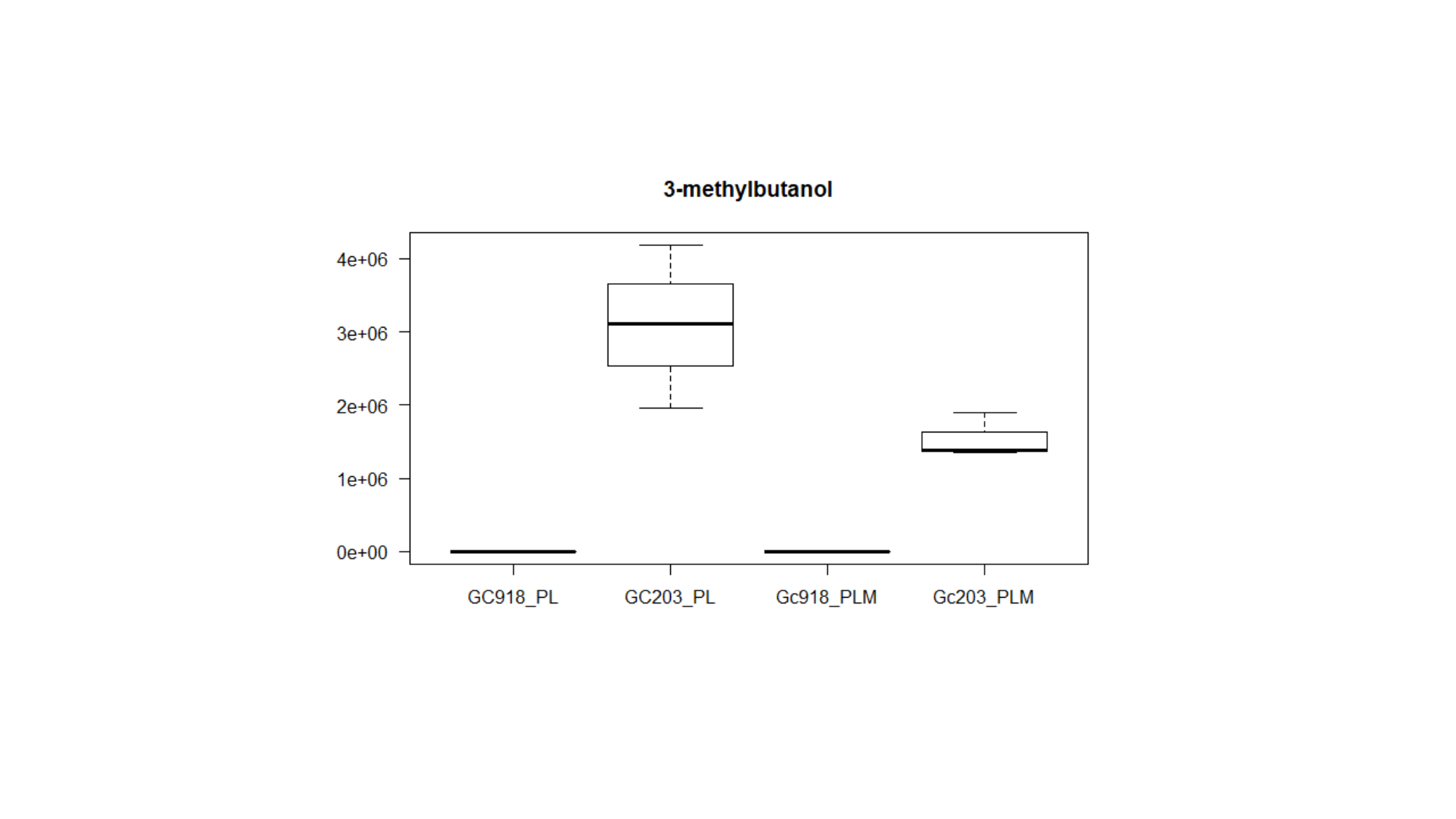

## Slide 17
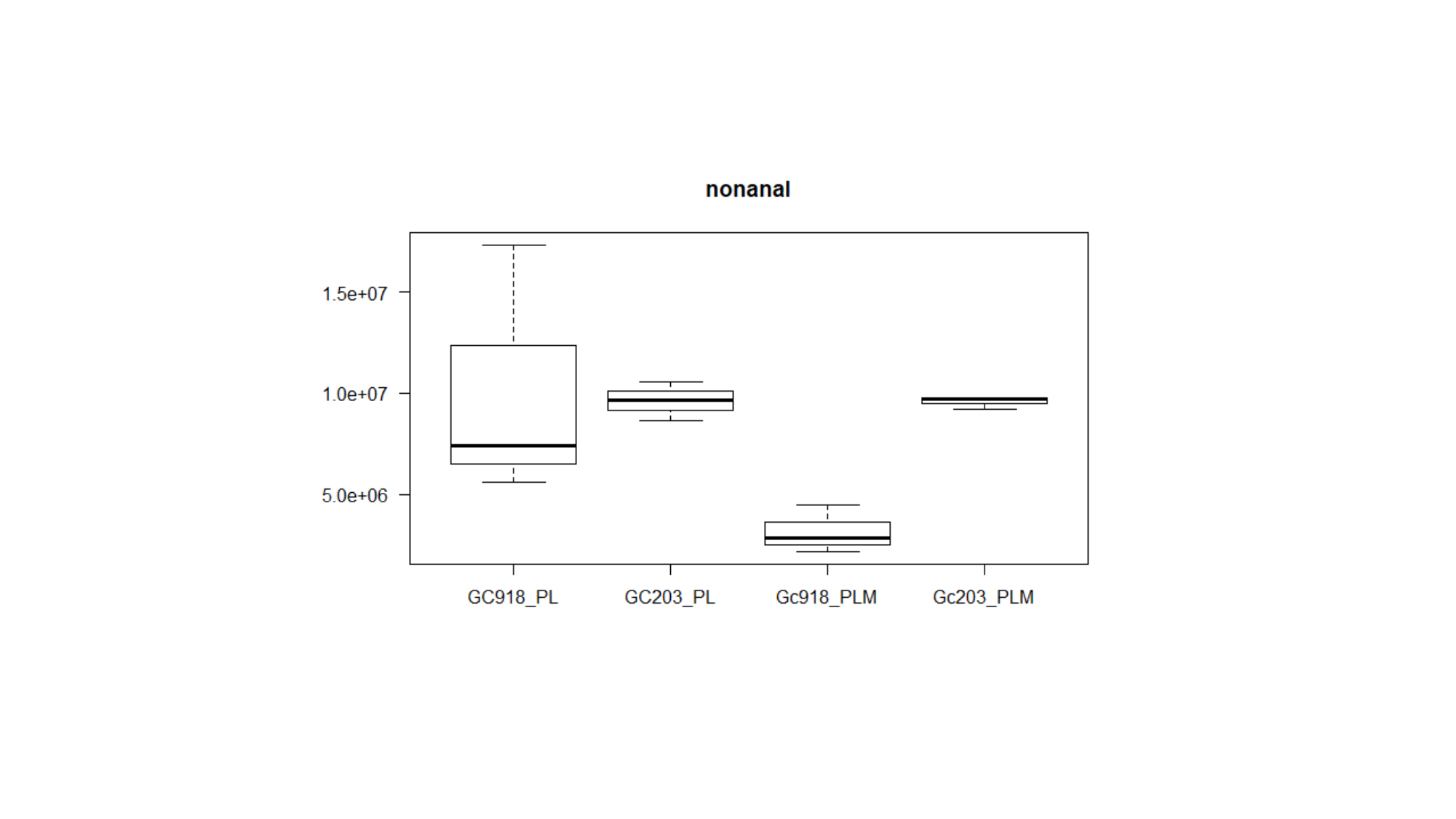

## Slide 18
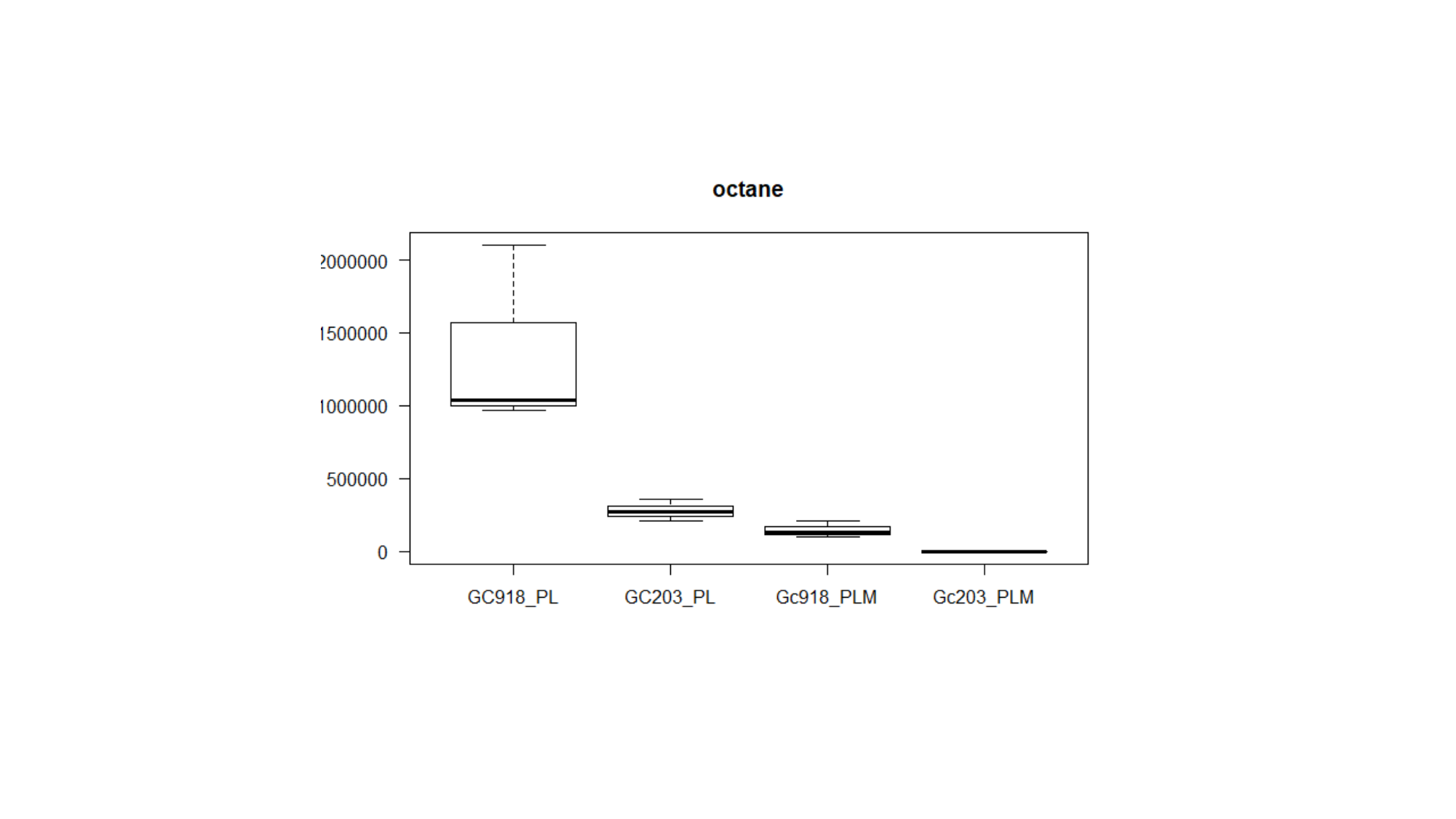

## Slide 19
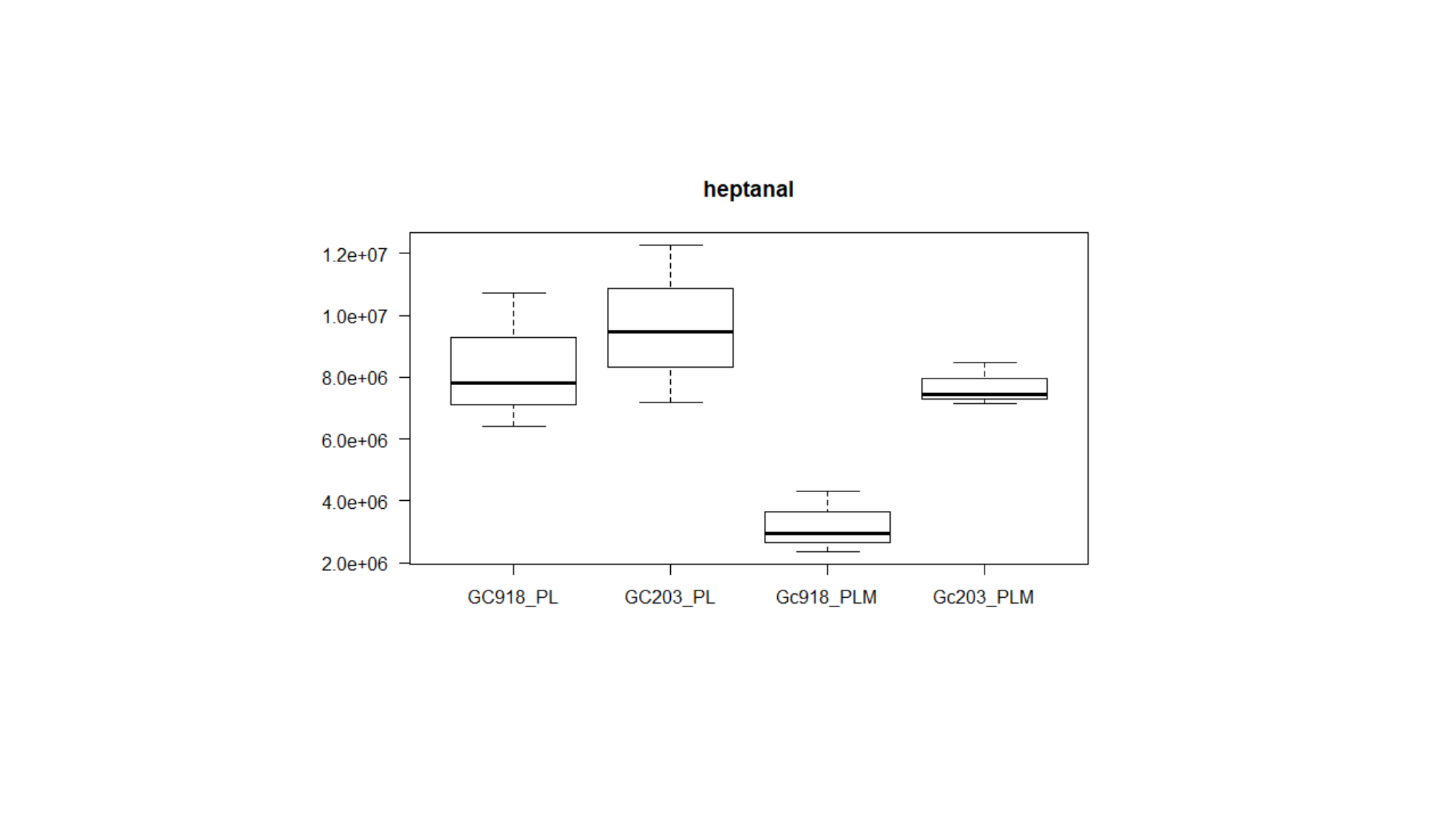

## Slide 20
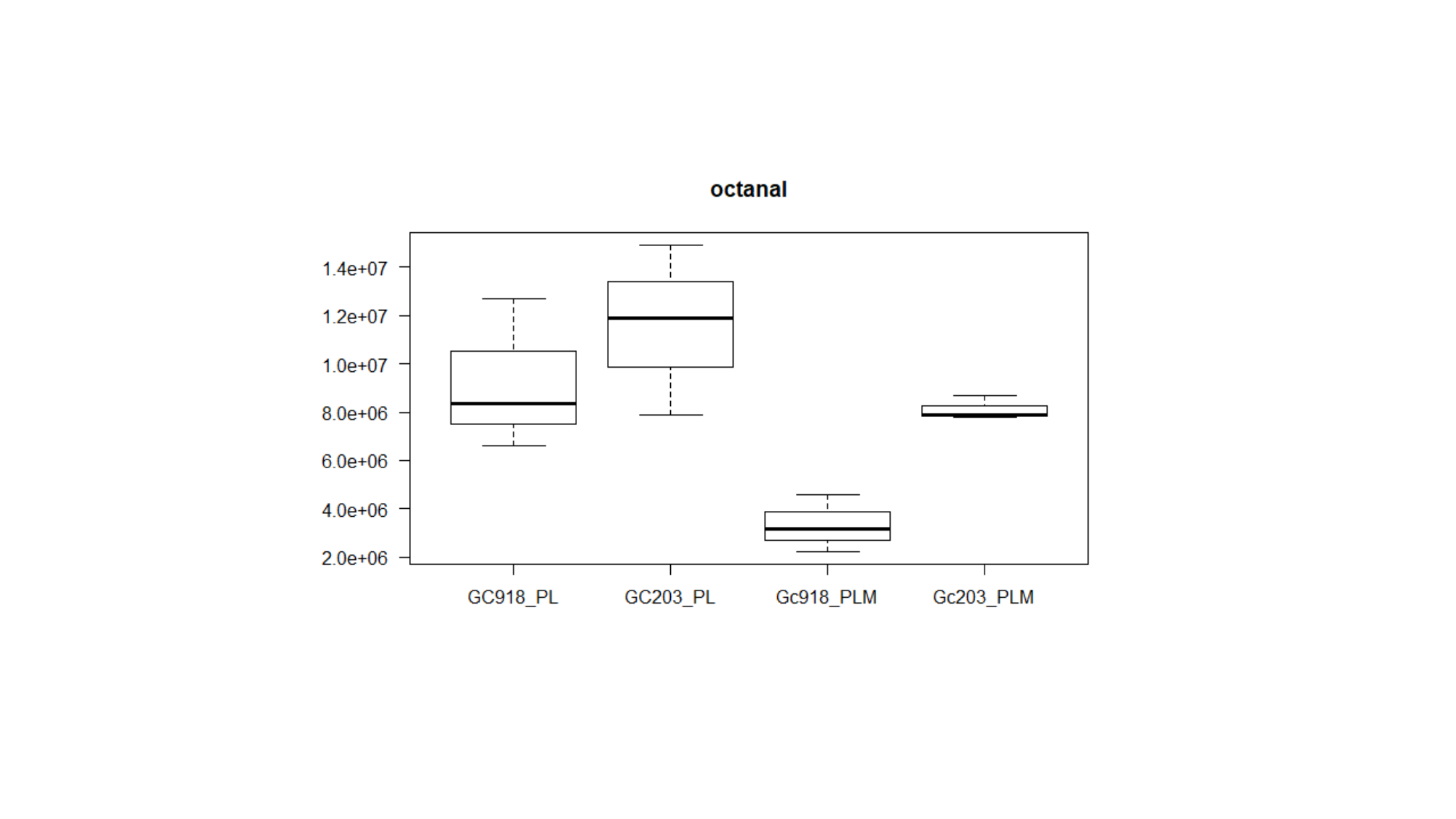

## Slide 21
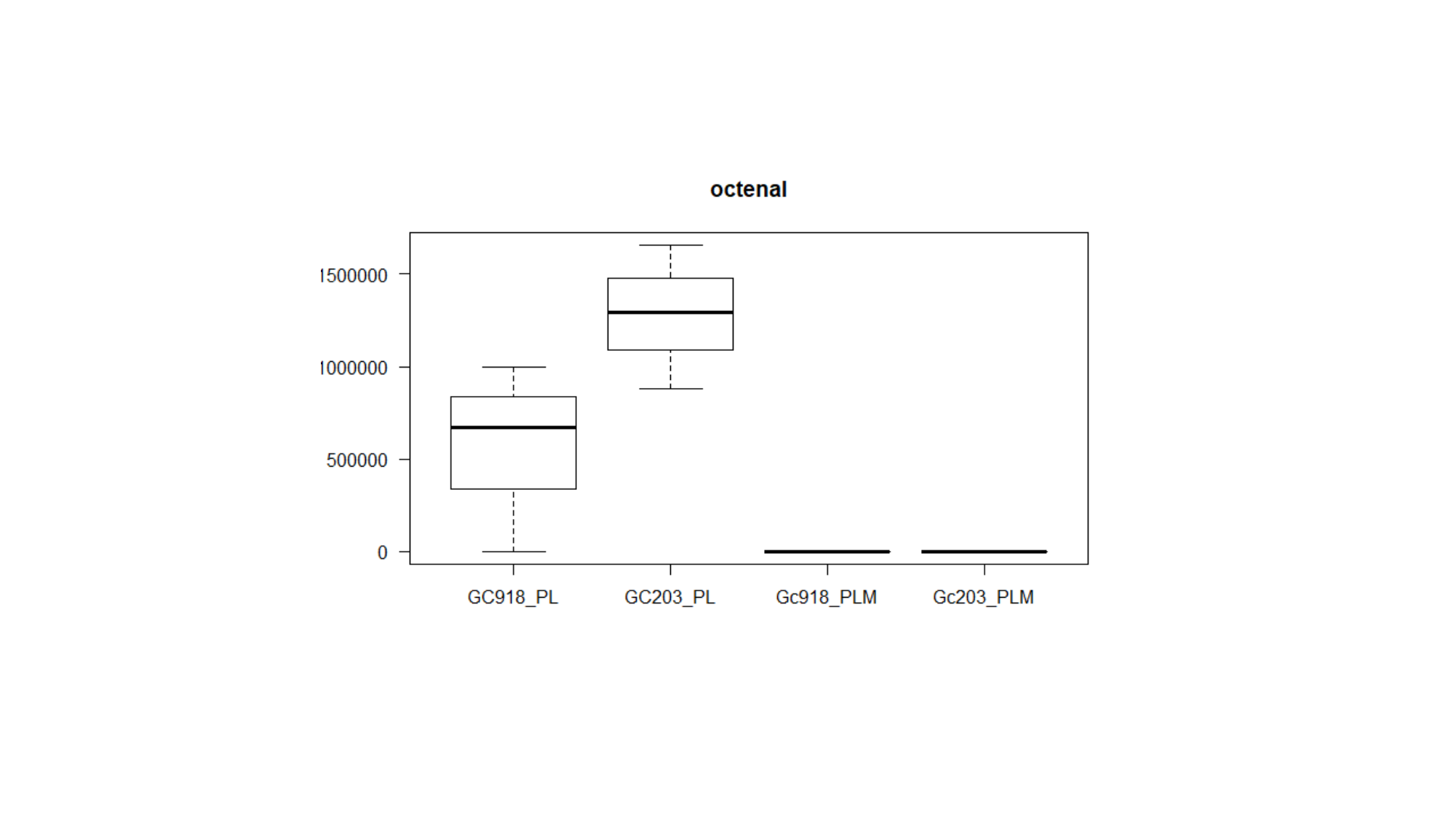

## Slide 22
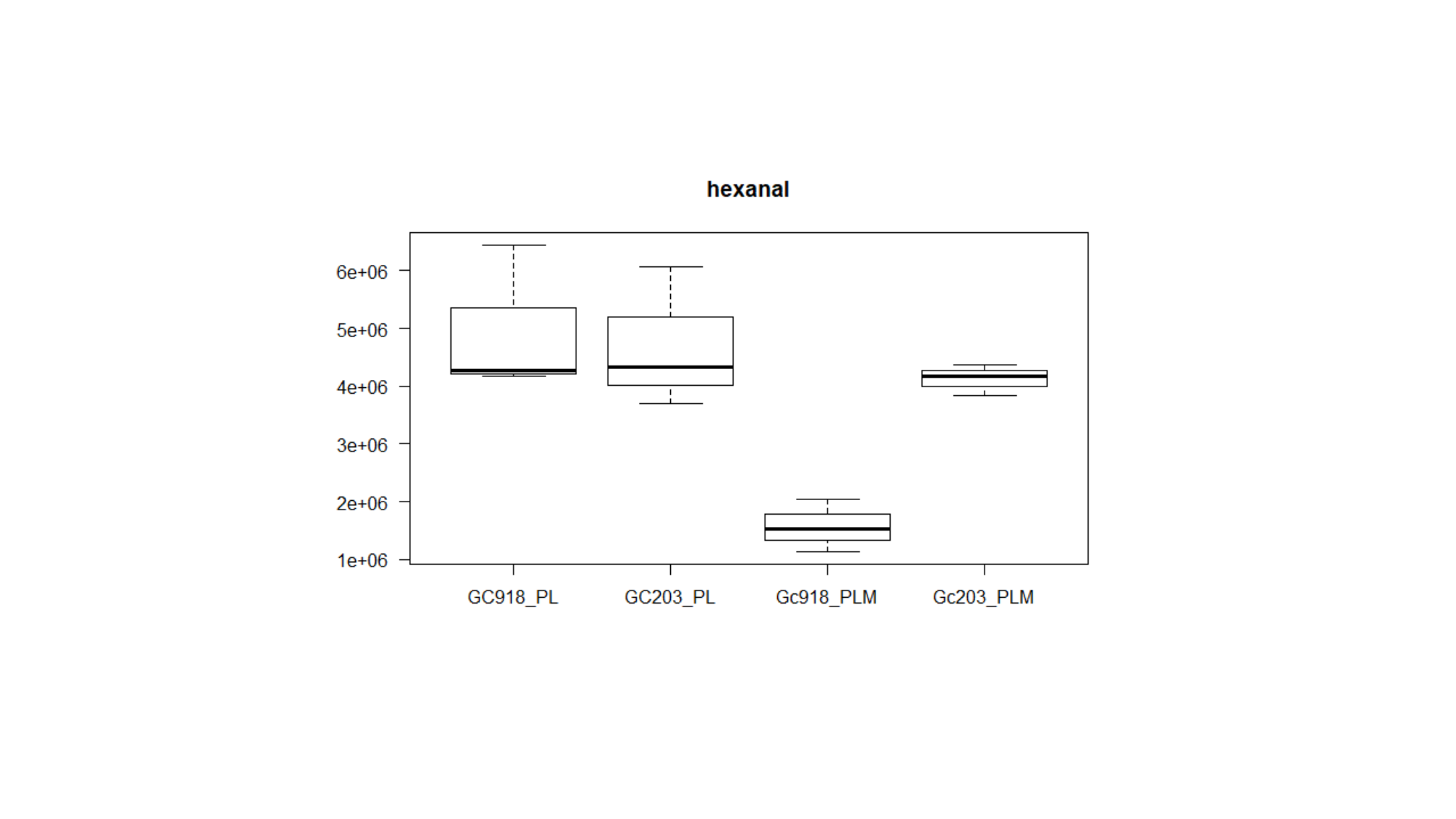

## Slide 23
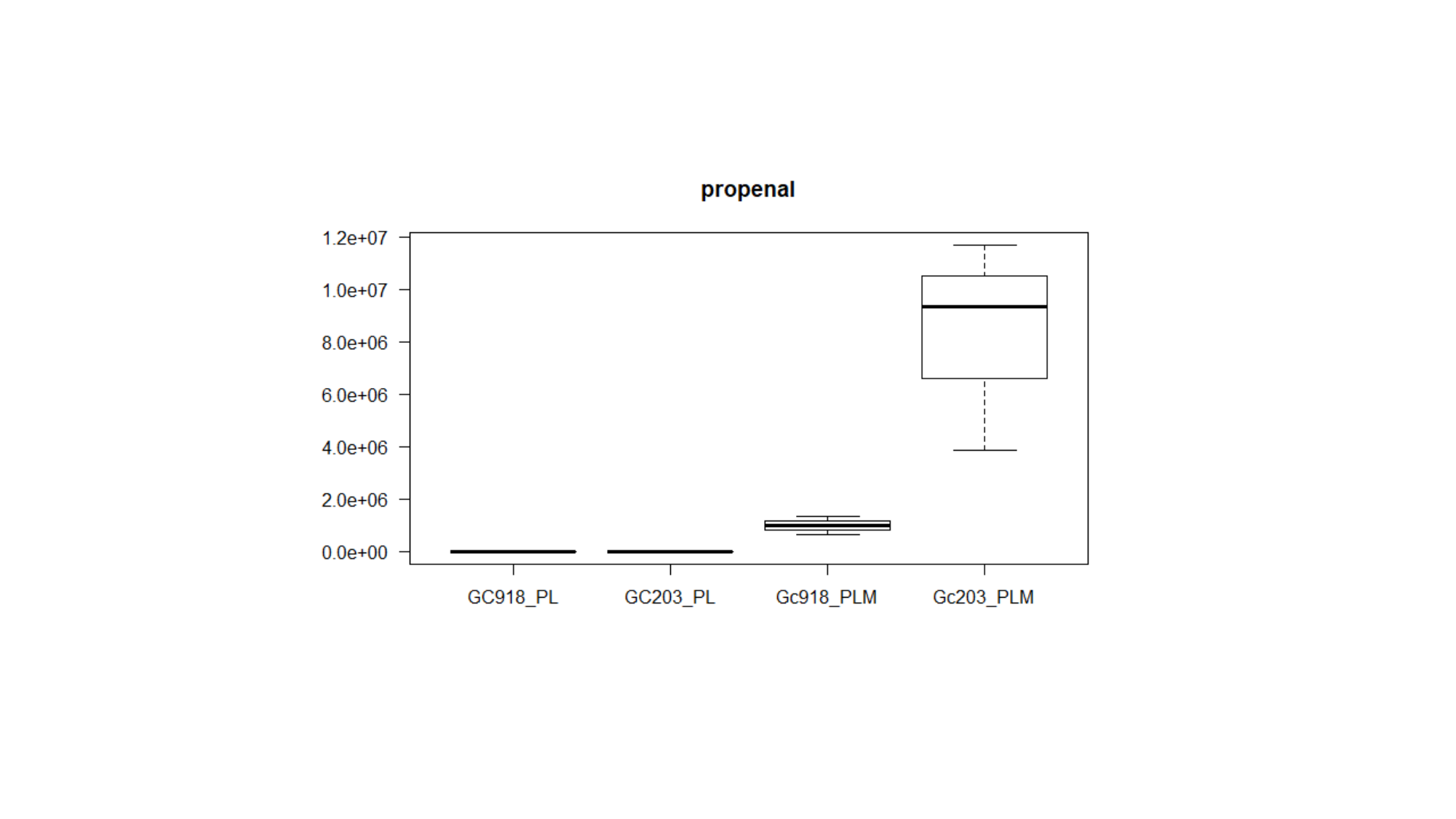

## Slide 24
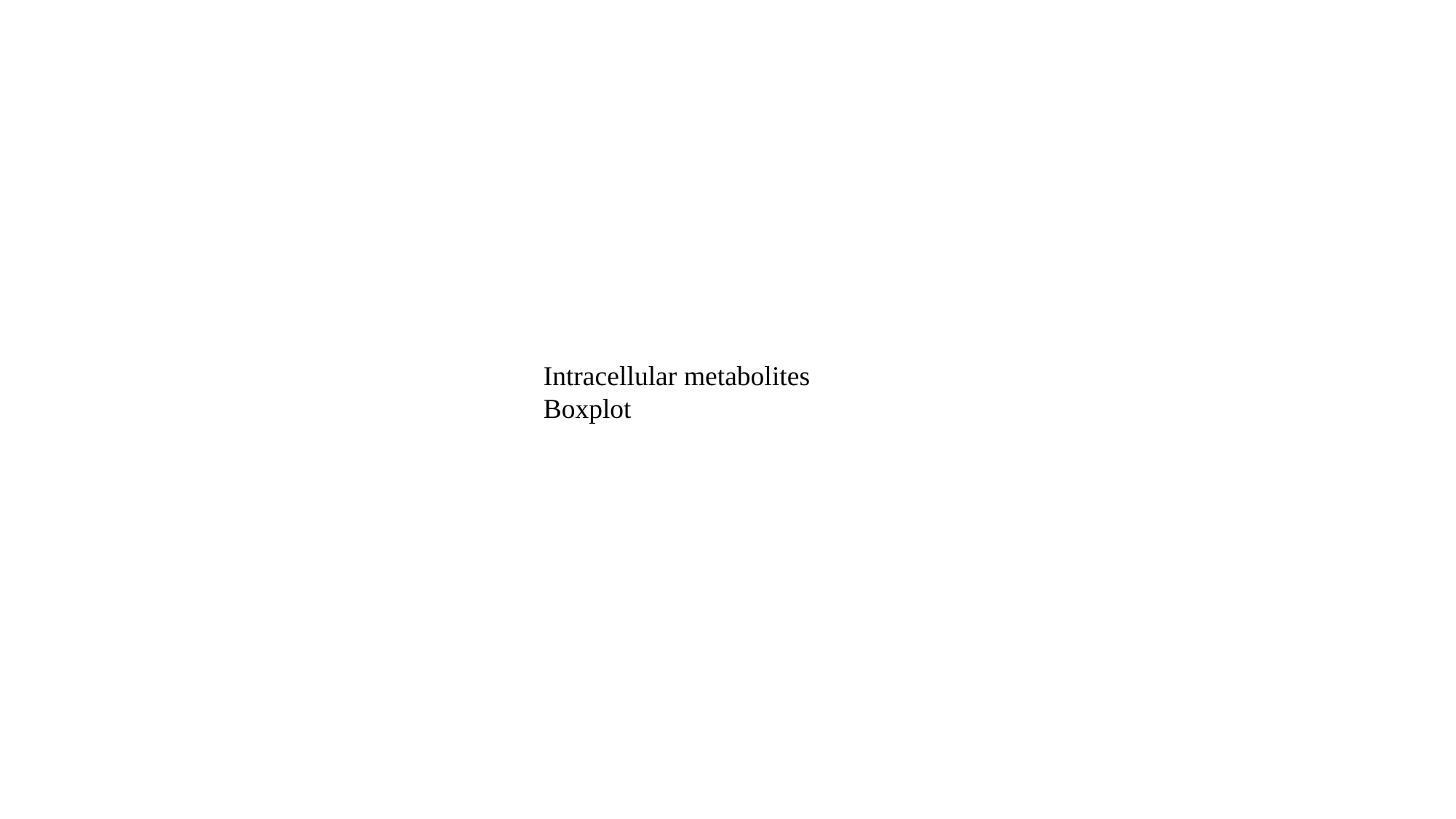

Intracellular metabolites
Boxplot

## Slide 25
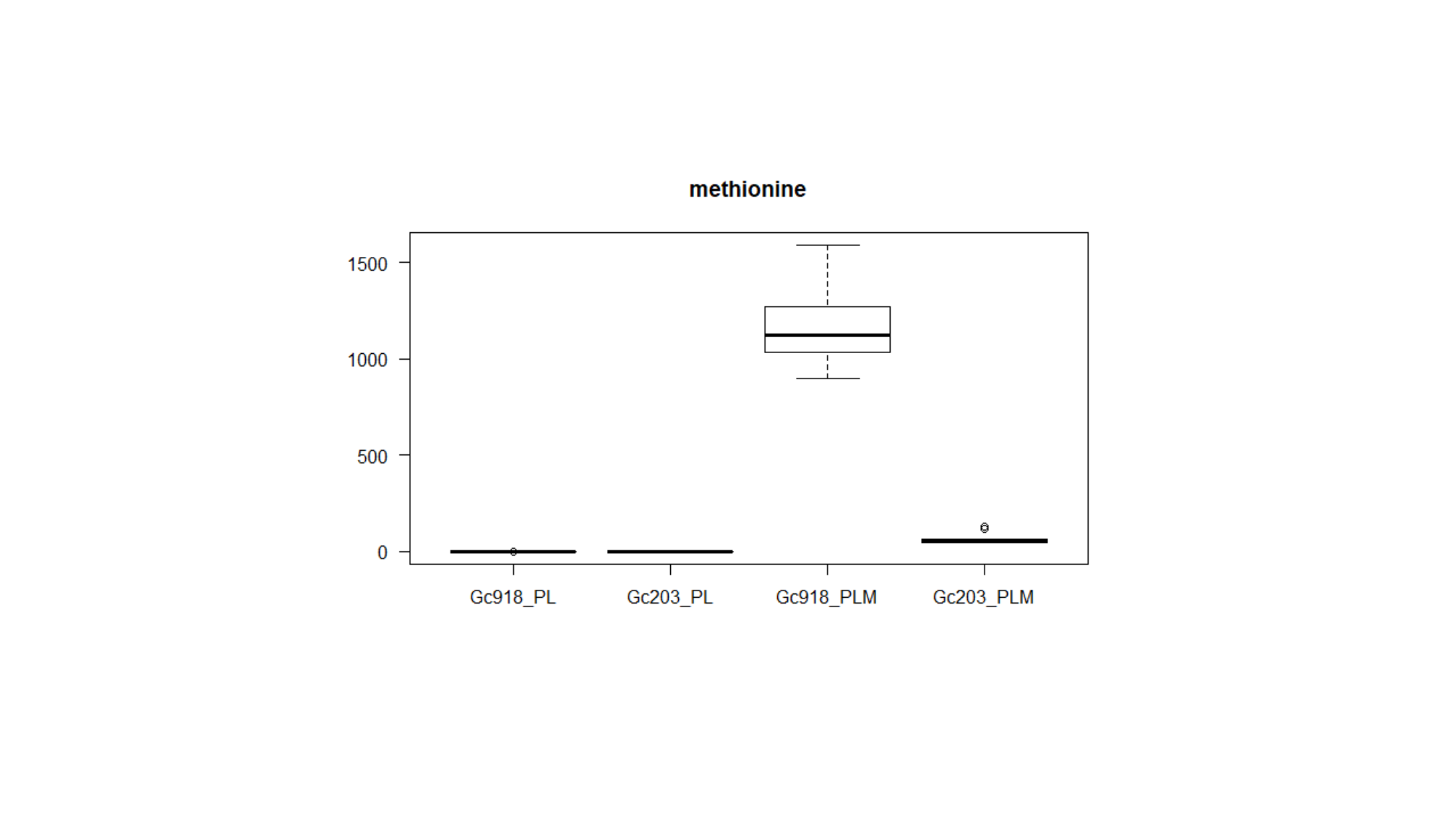

## Slide 26
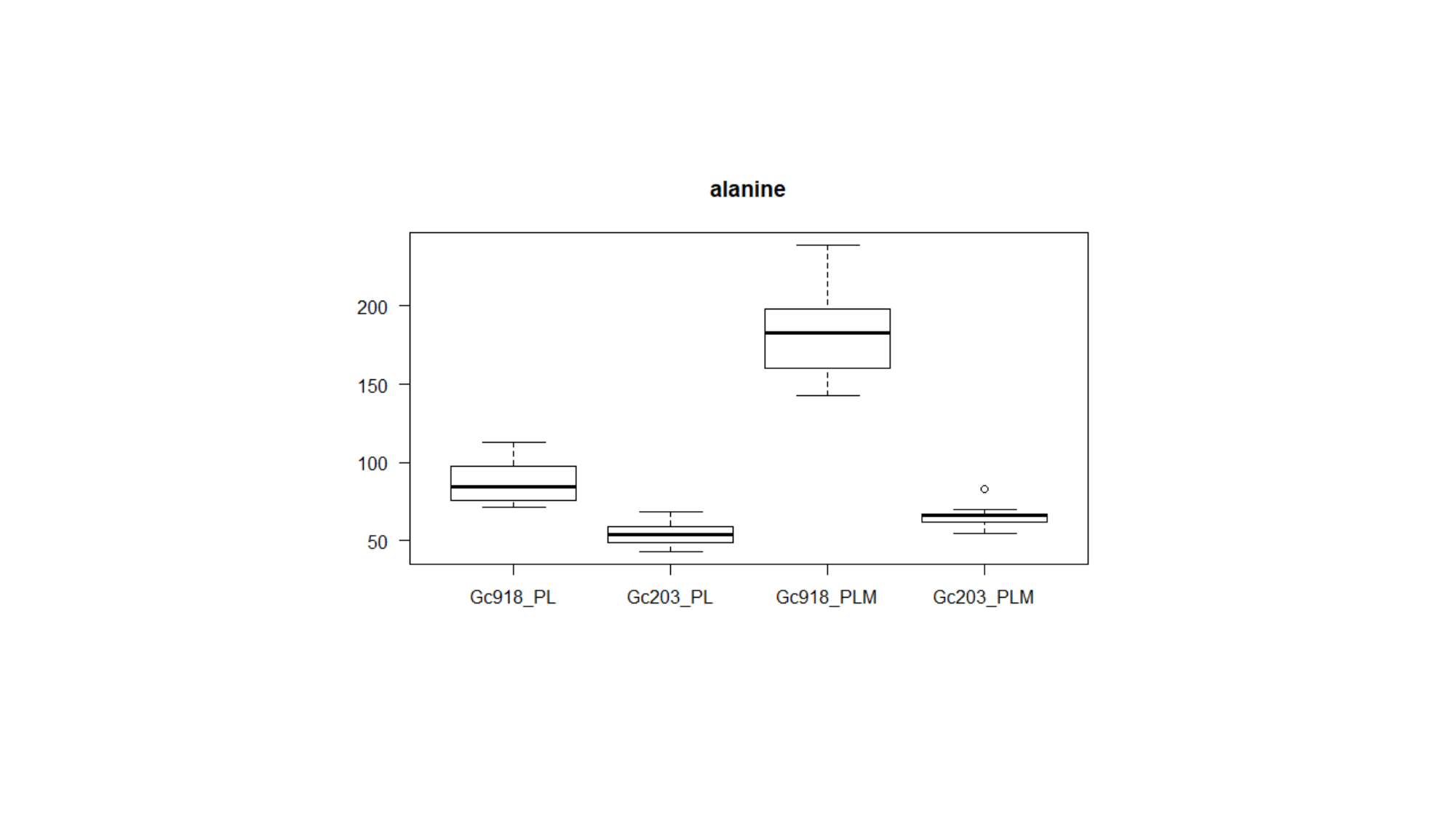

## Slide 27
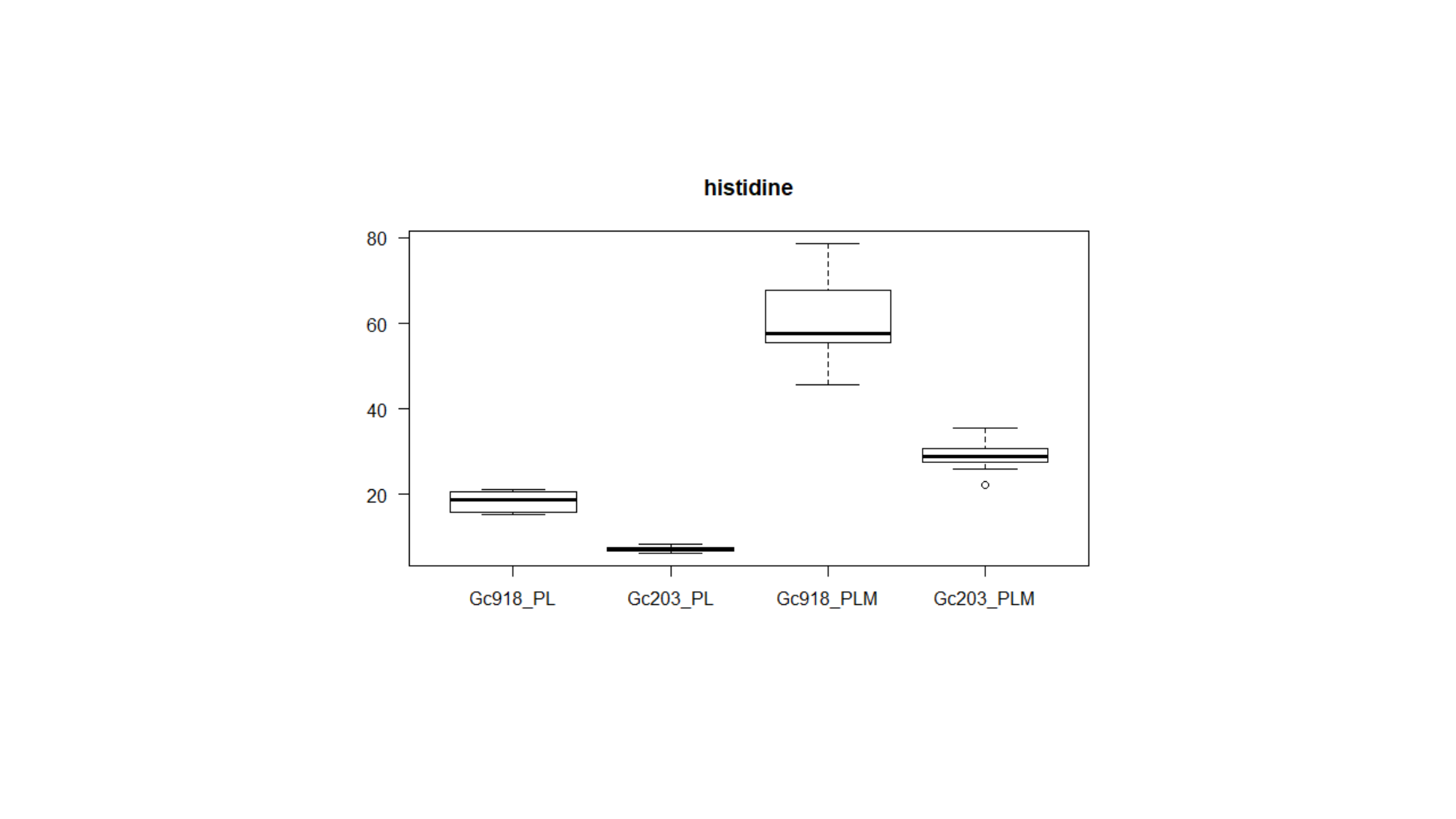

## Slide 28
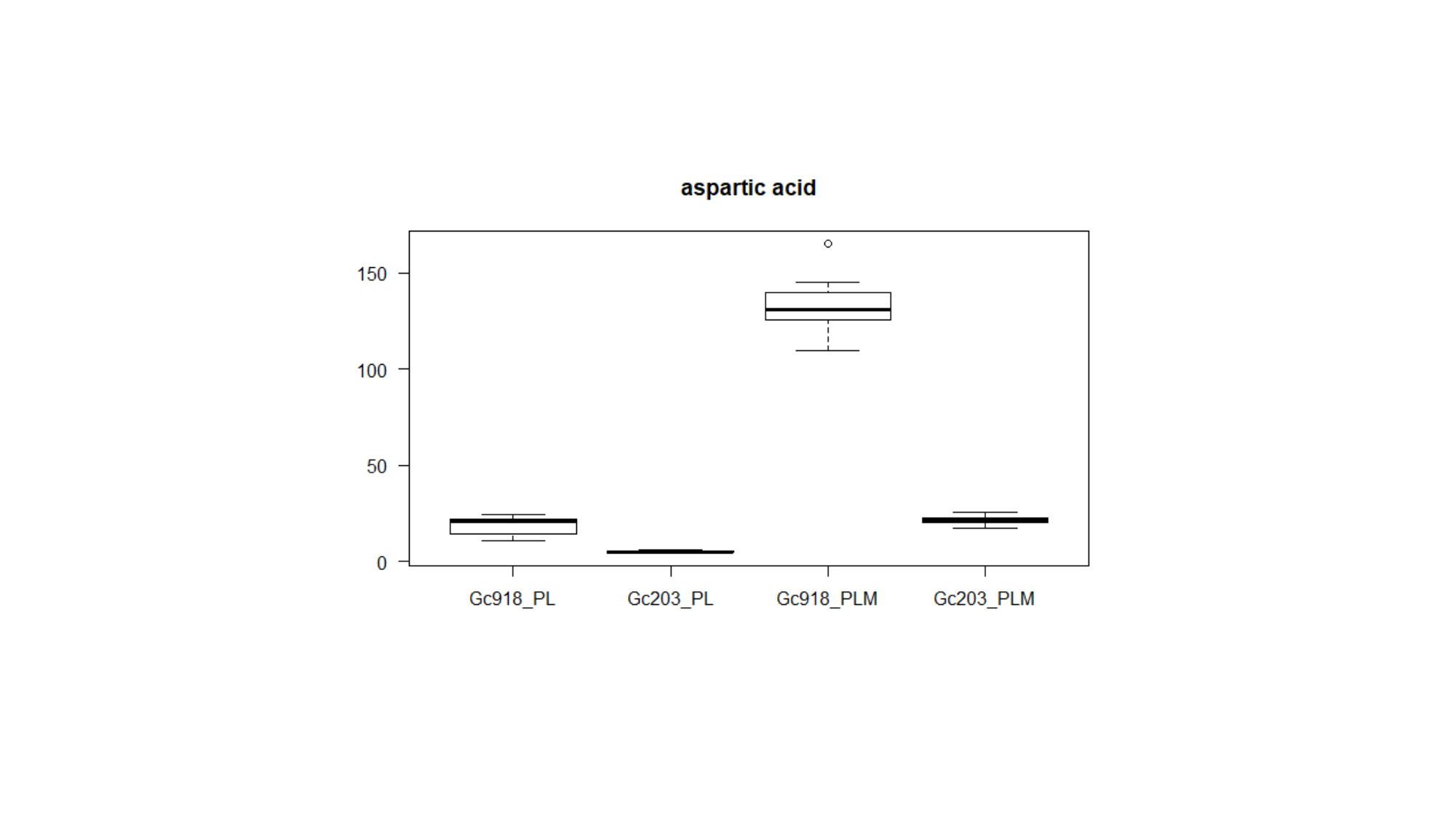

## Slide 29
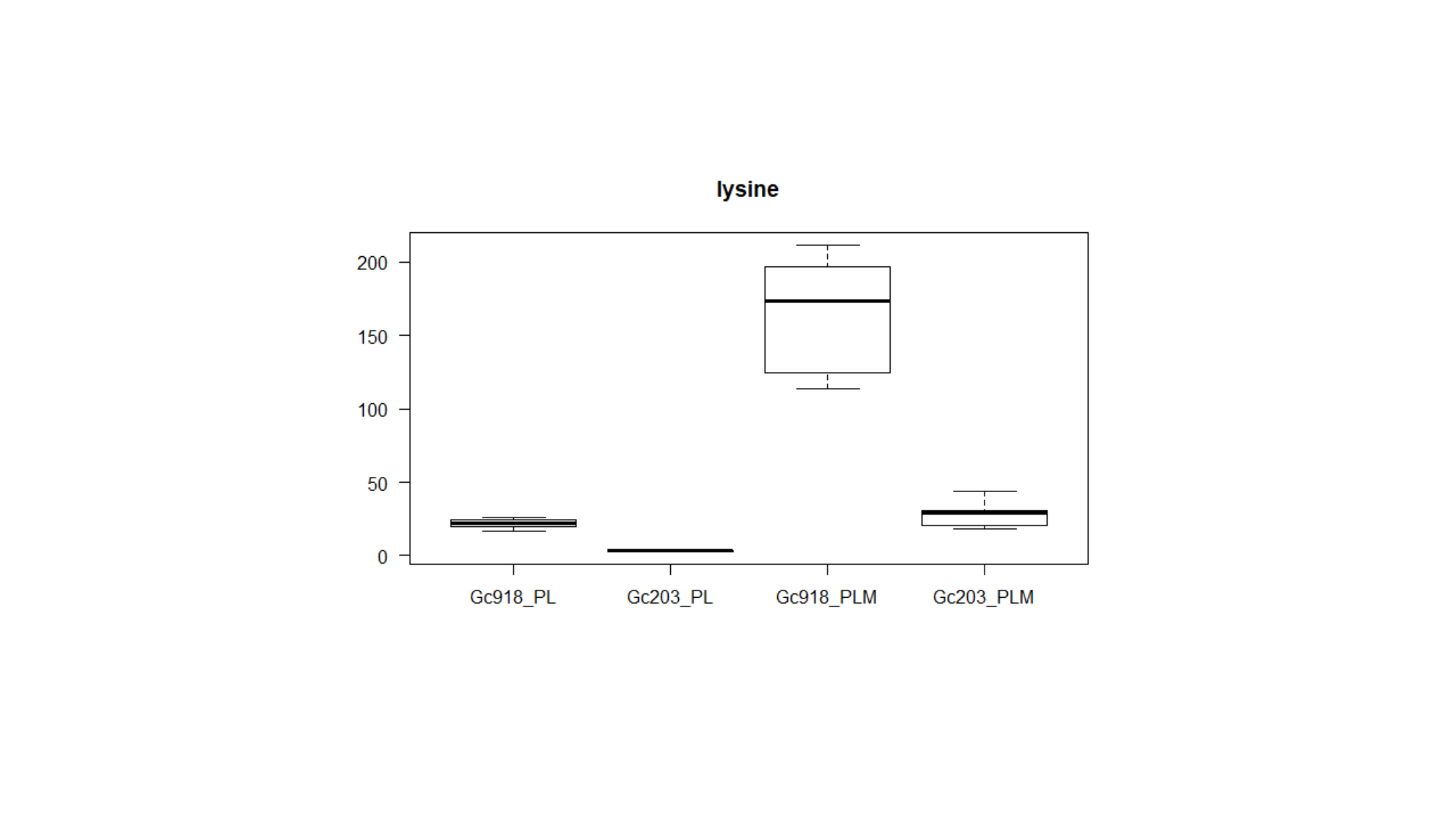

## Slide 30
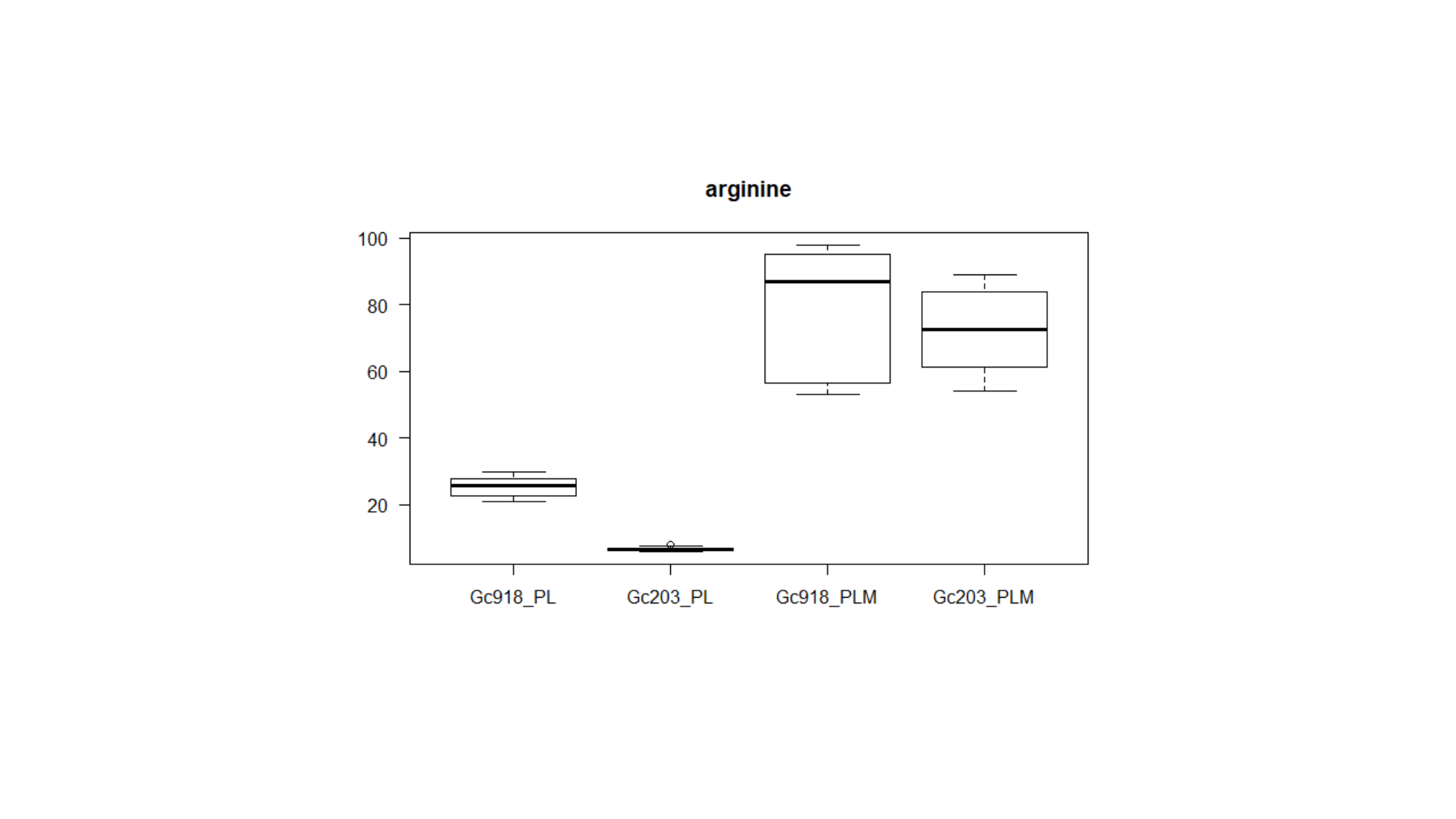

## Slide 31
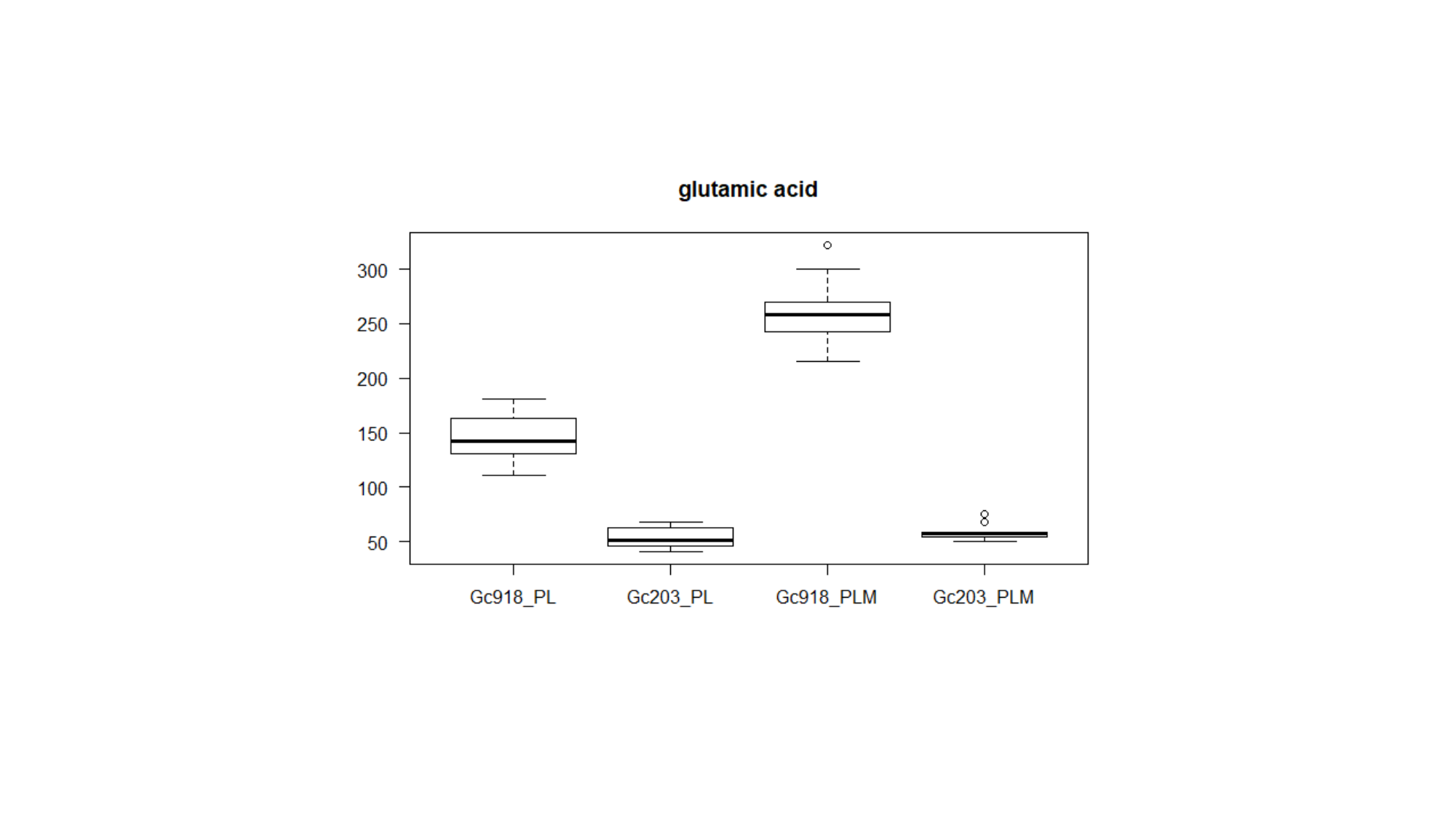

## Slide 32
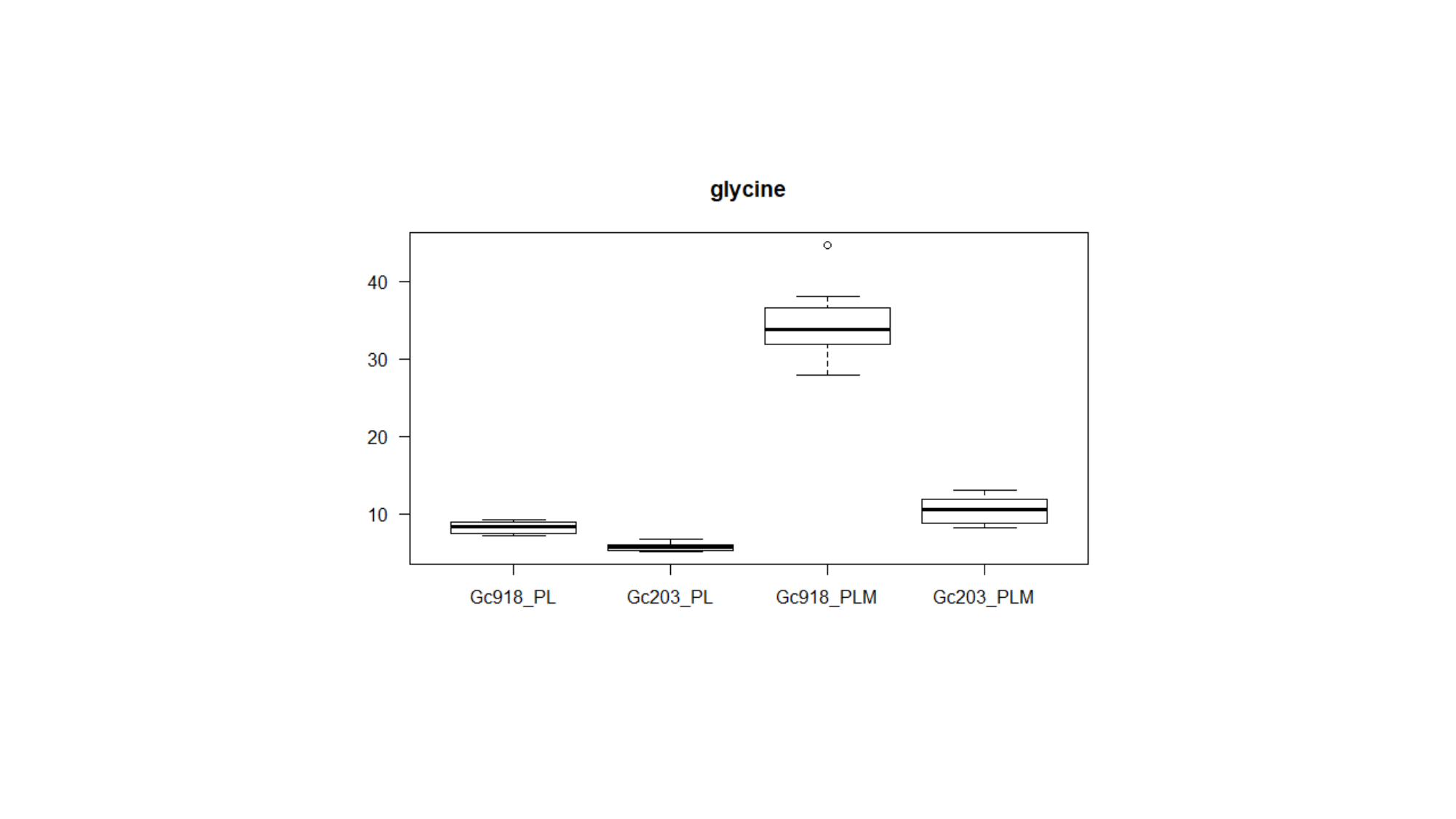

## Slide 33
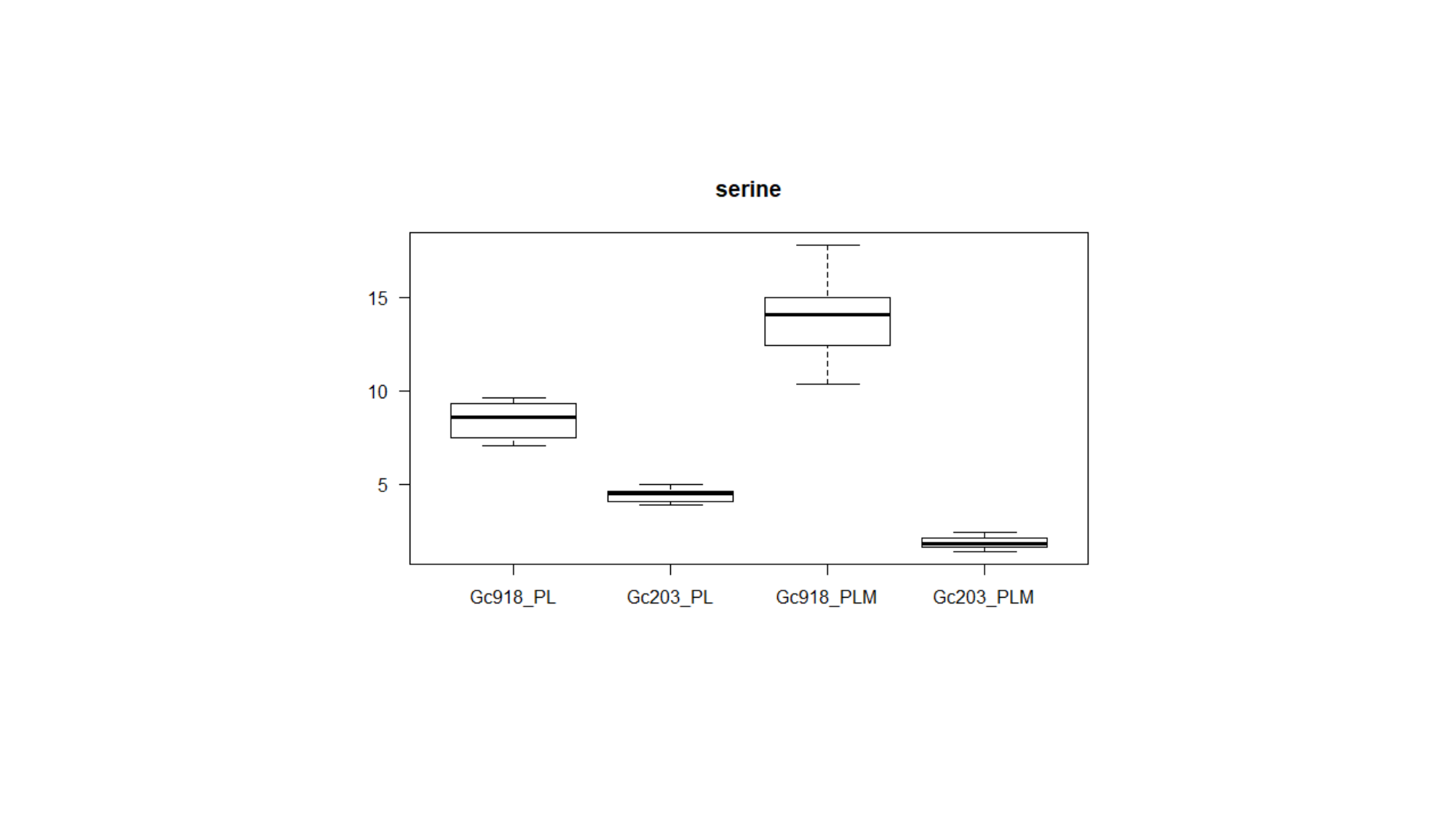

## Slide 34
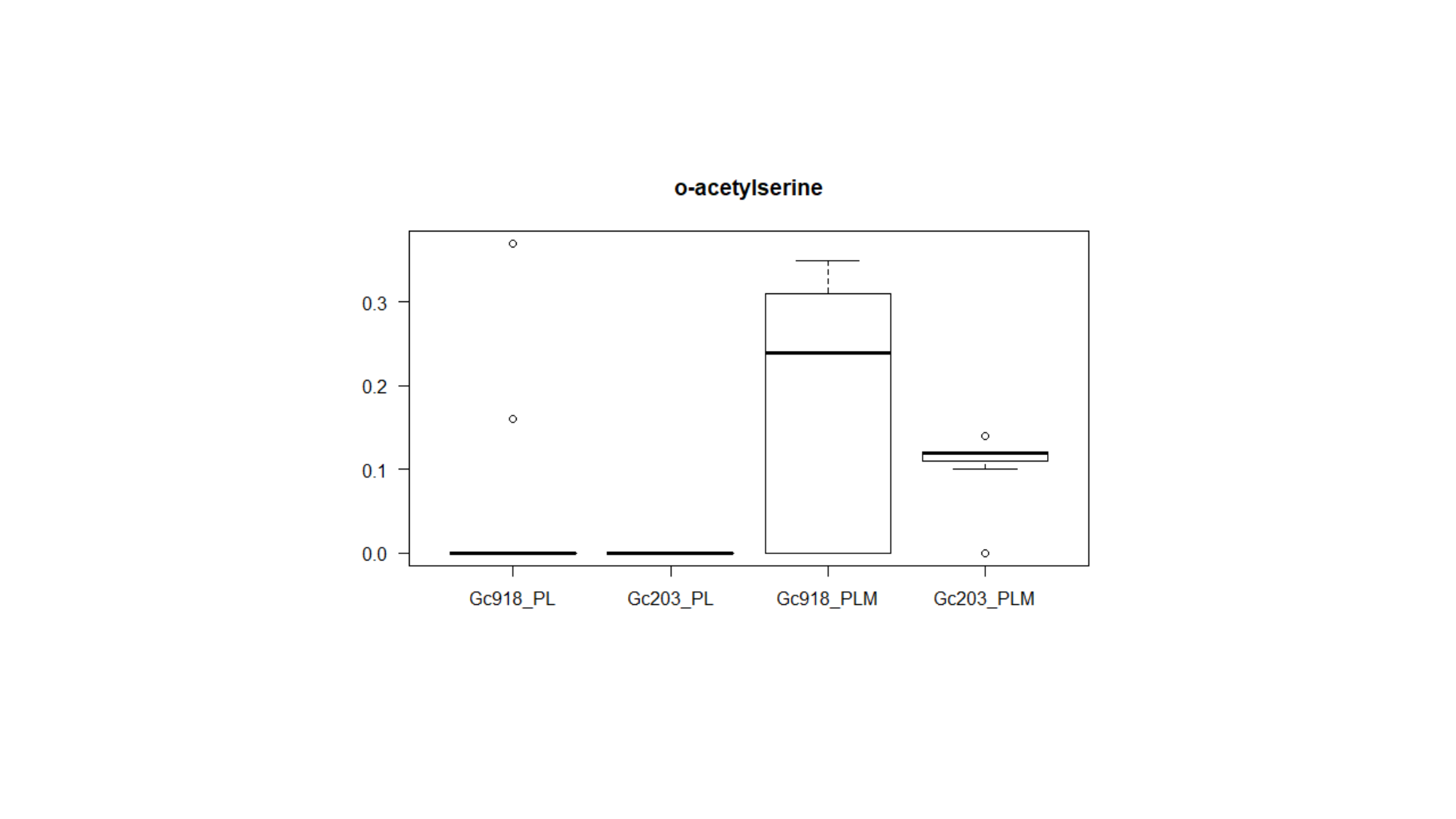

## Slide 35
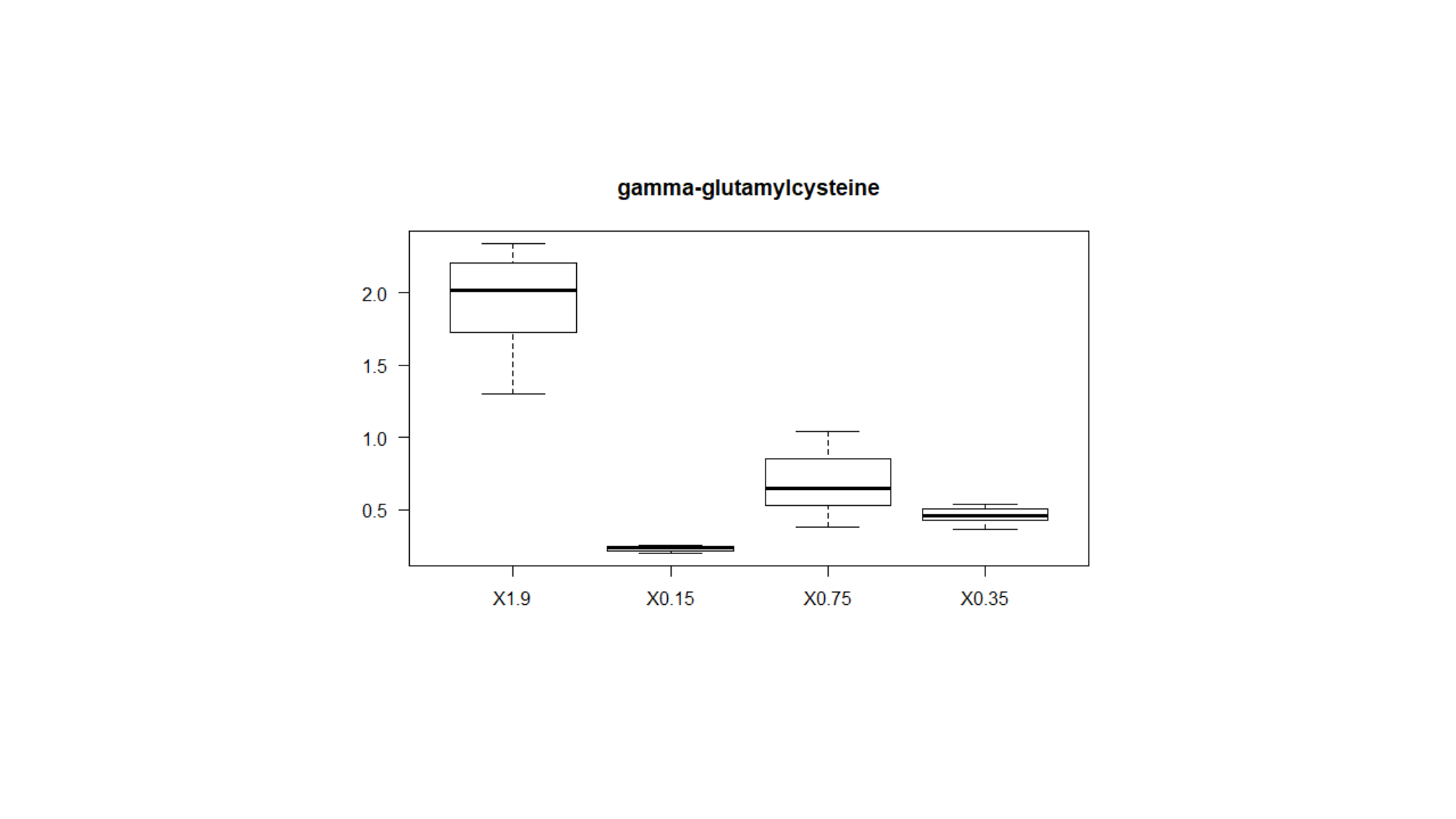

## Slide 36
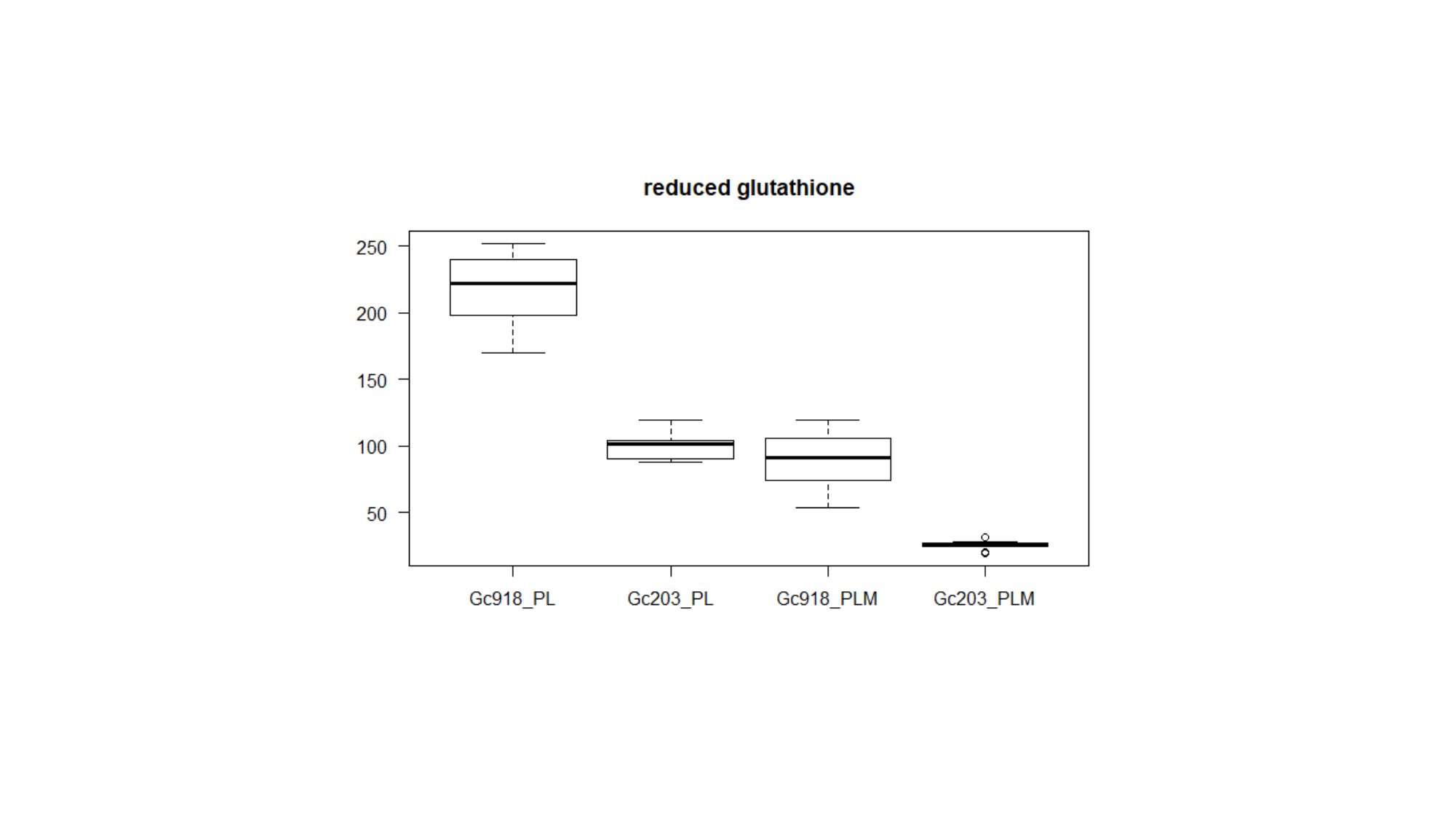

## Slide 37
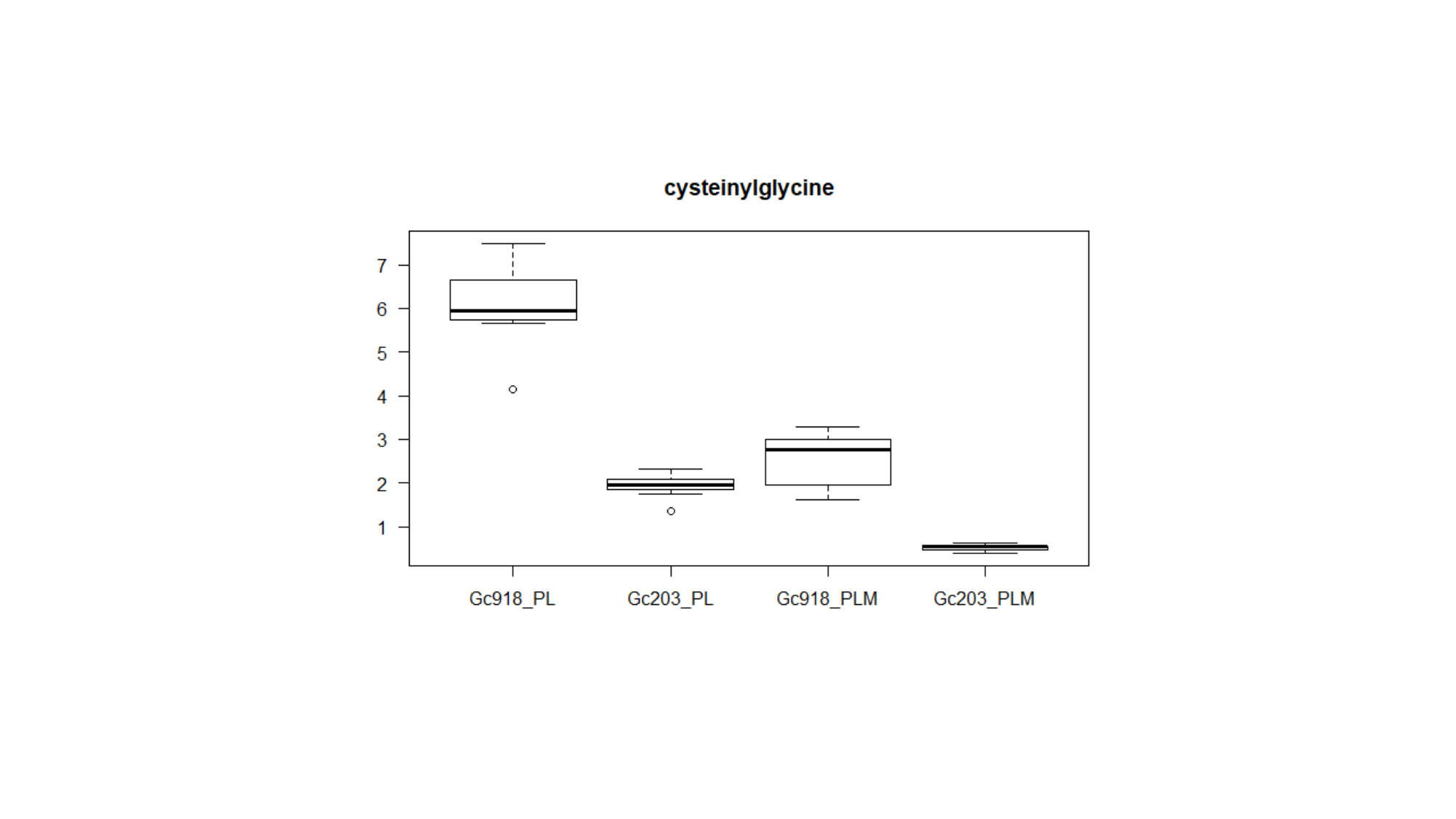

## Slide 38
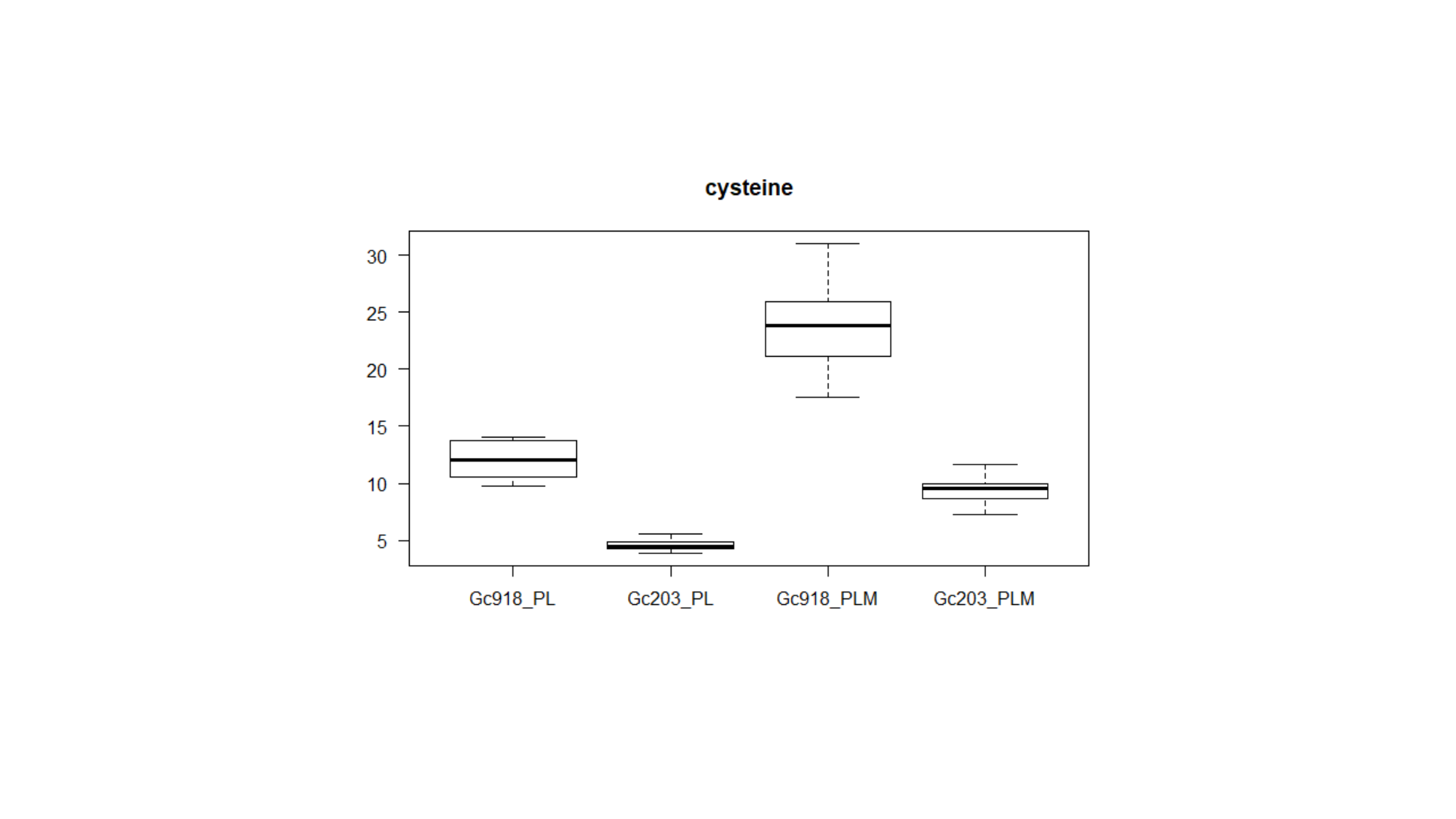

## Slide 39
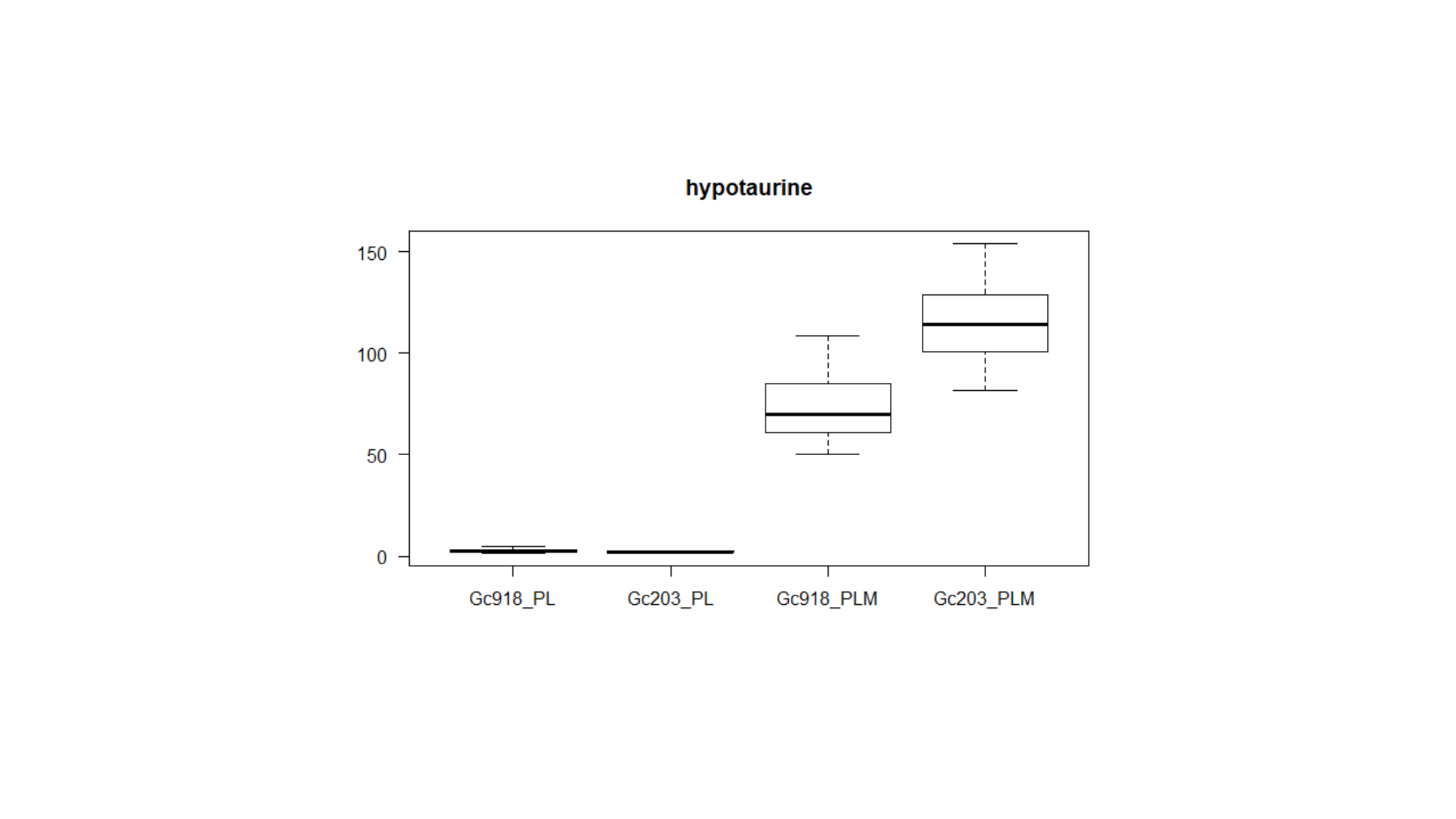

## Slide 40
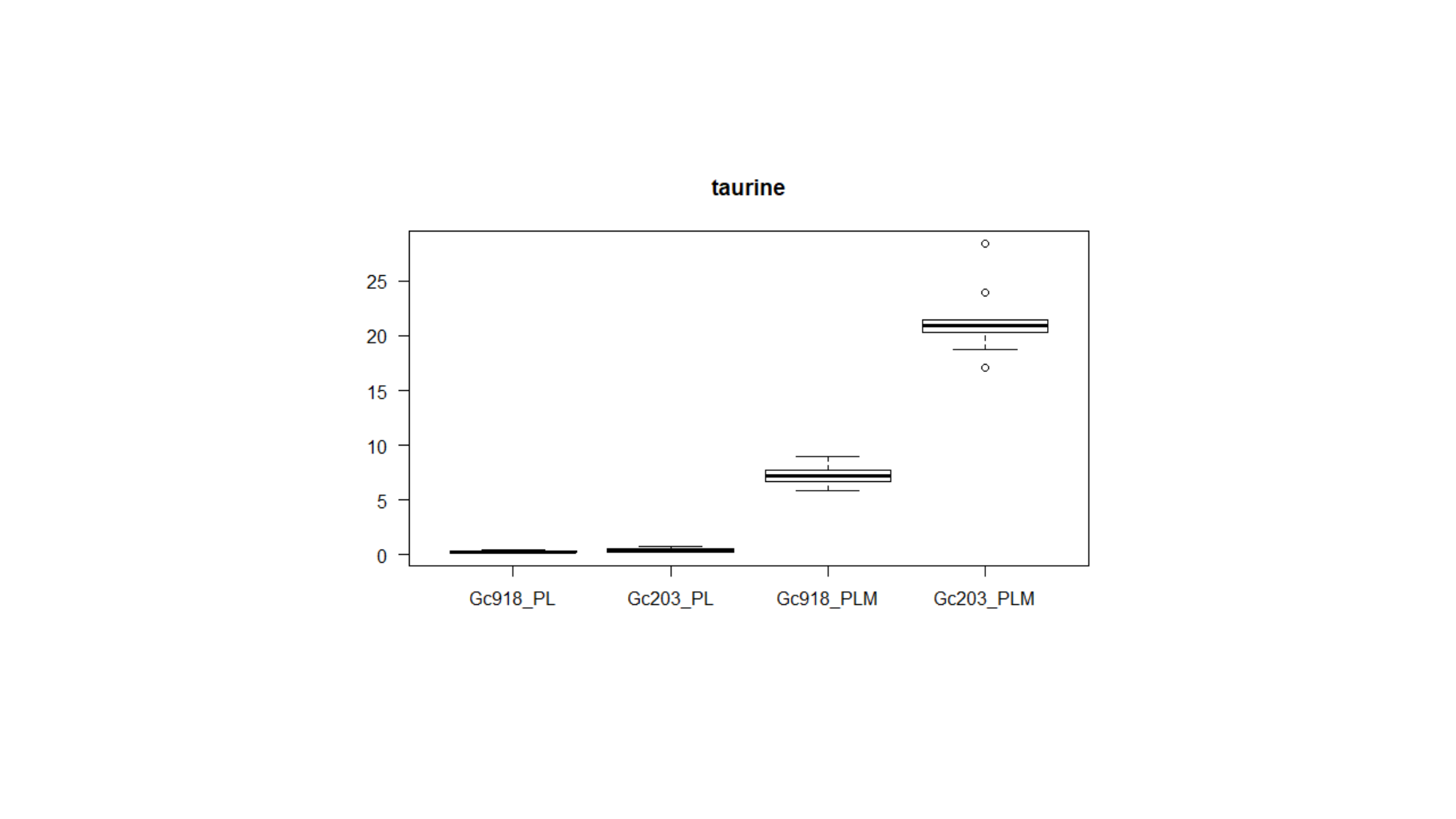

## Slide 41
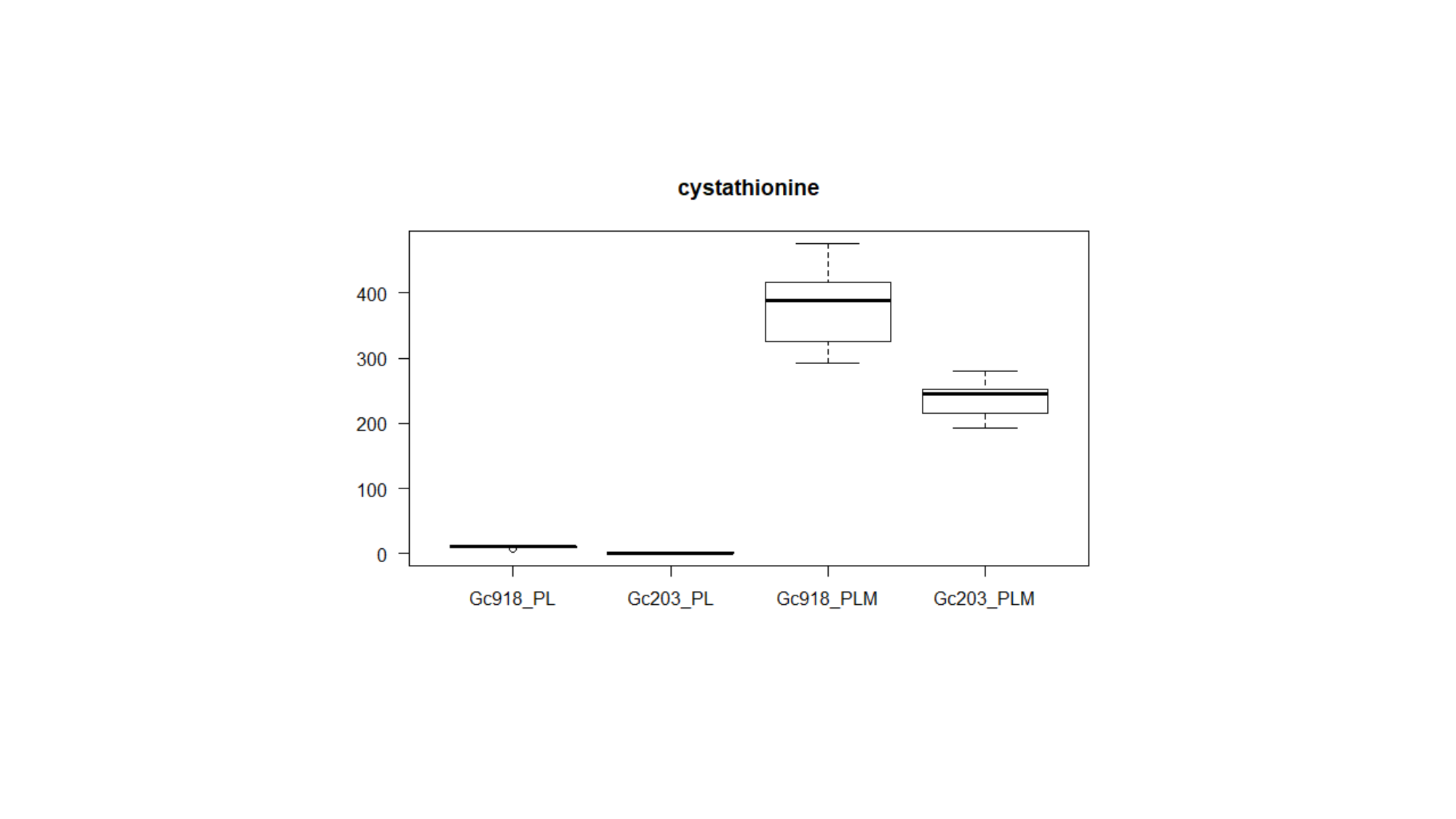

## Slide 42
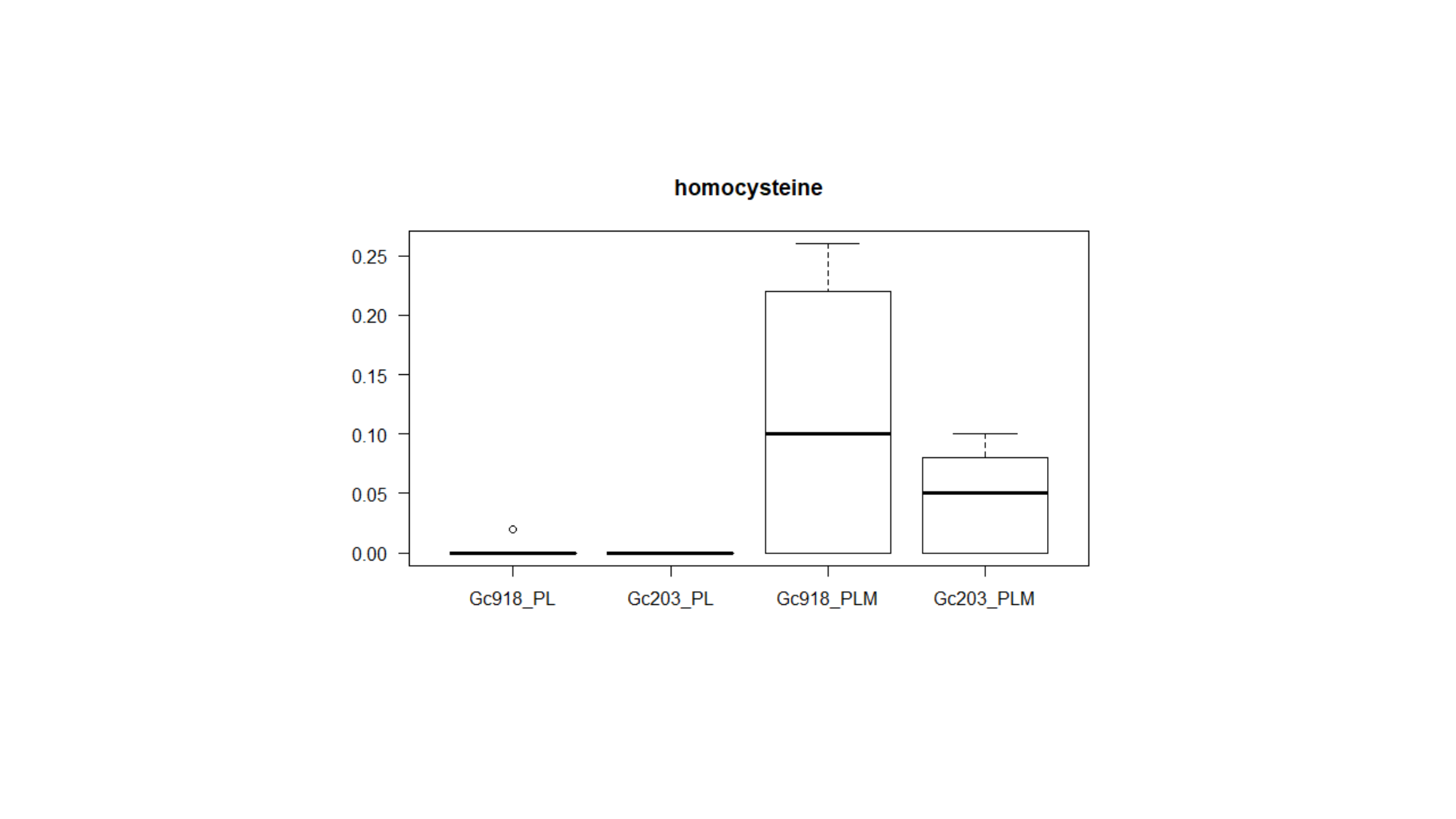

## Slide 43
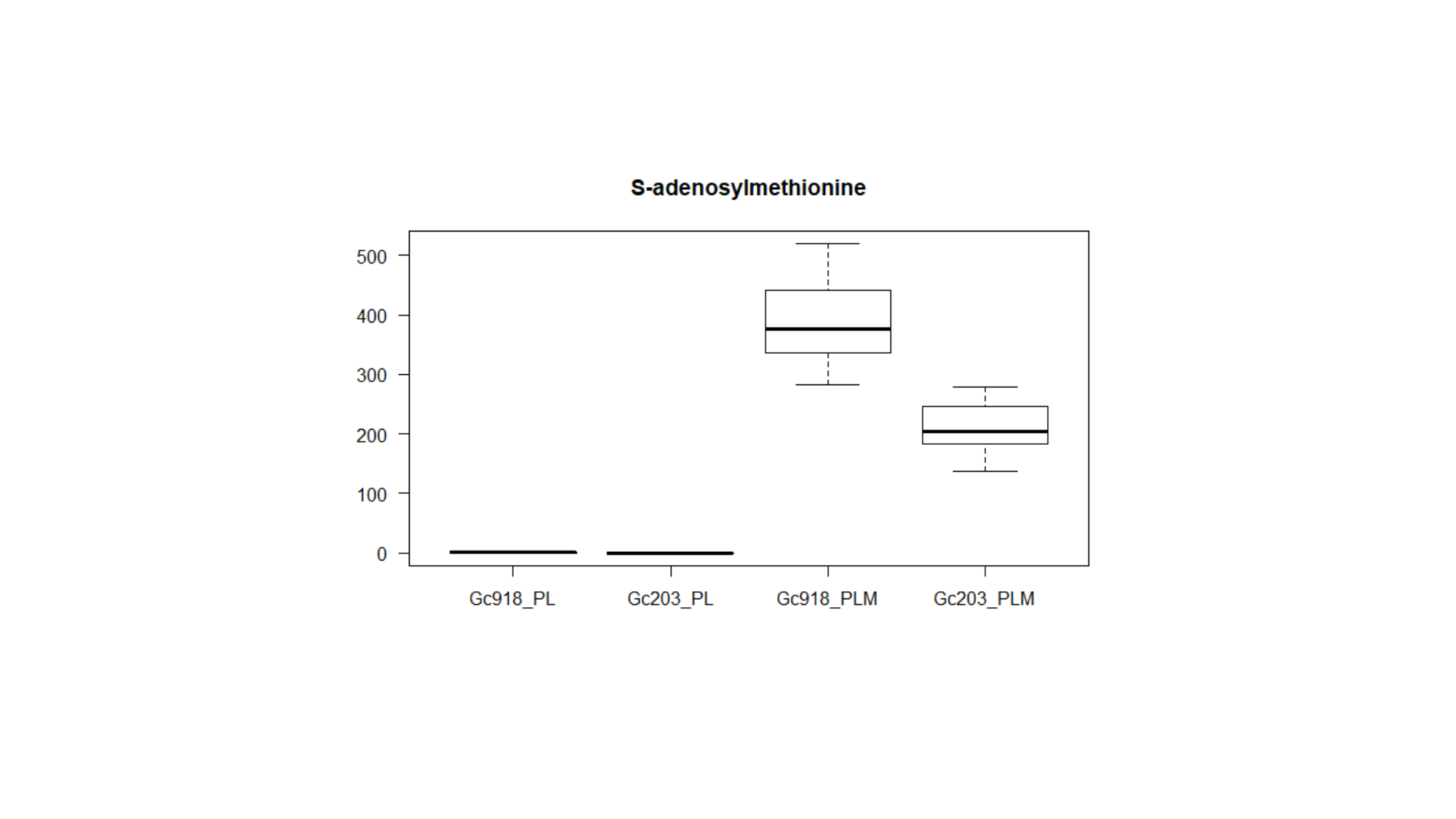

## Slide 44
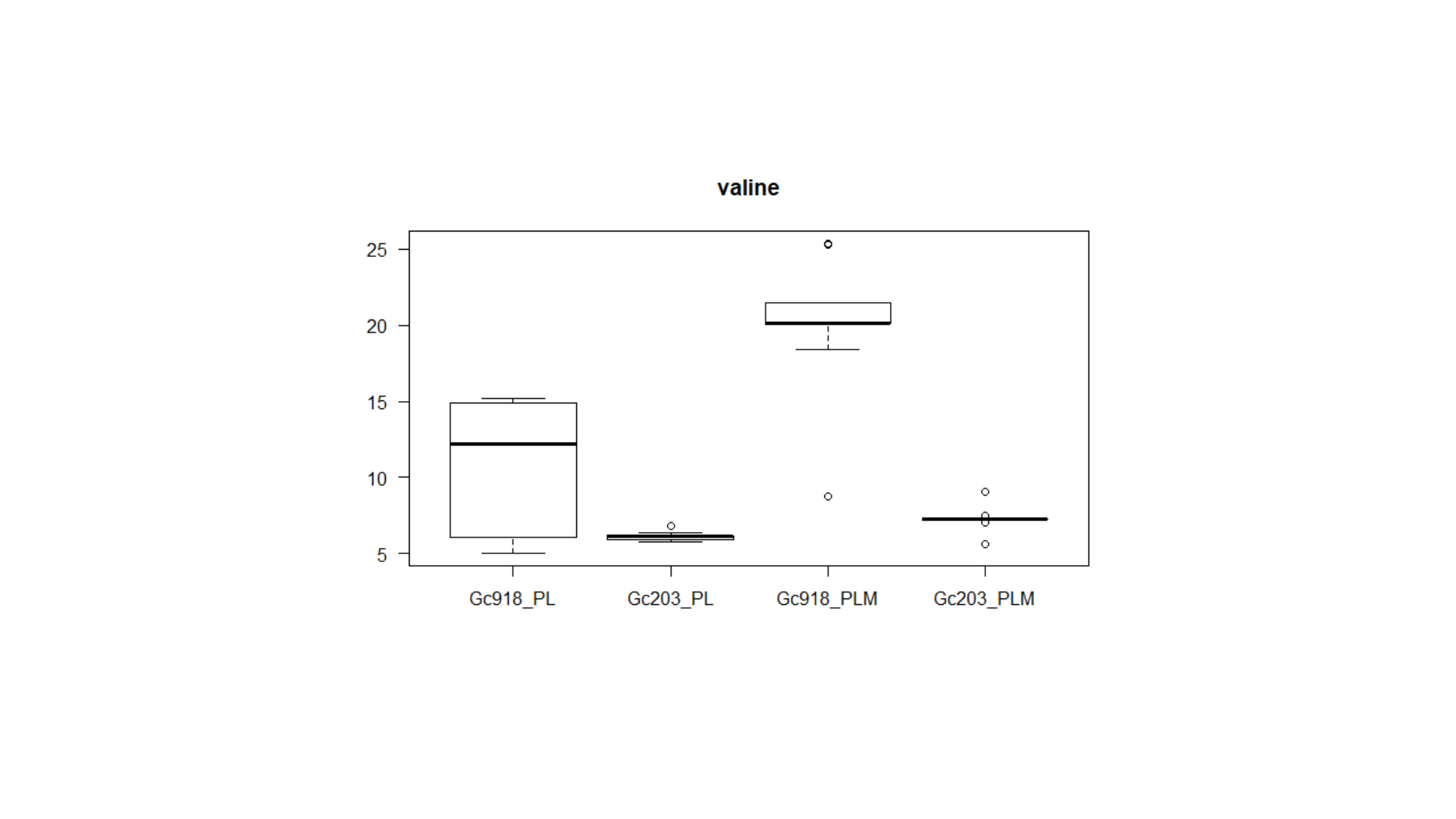

## Slide 45
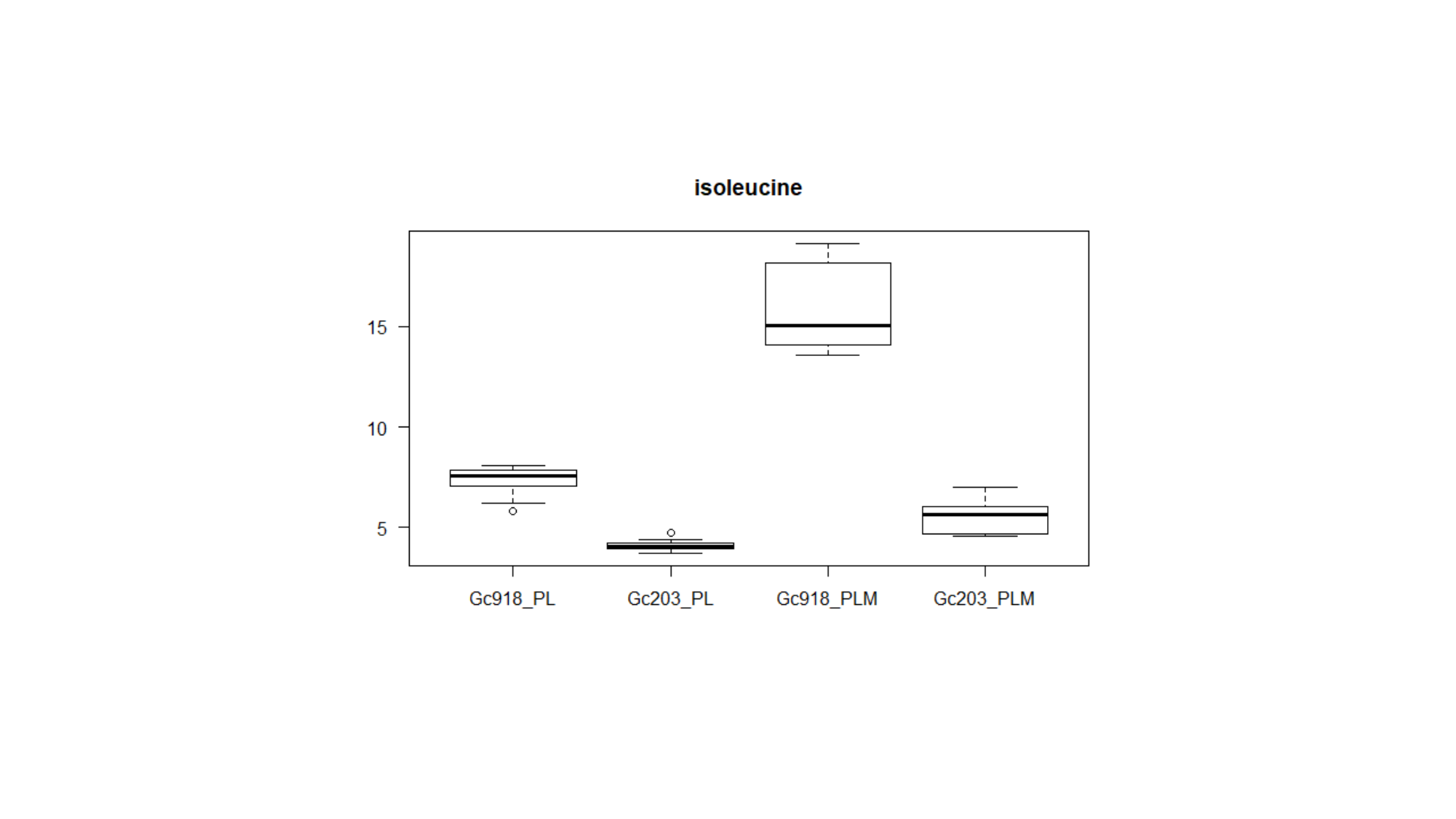

## Slide 46
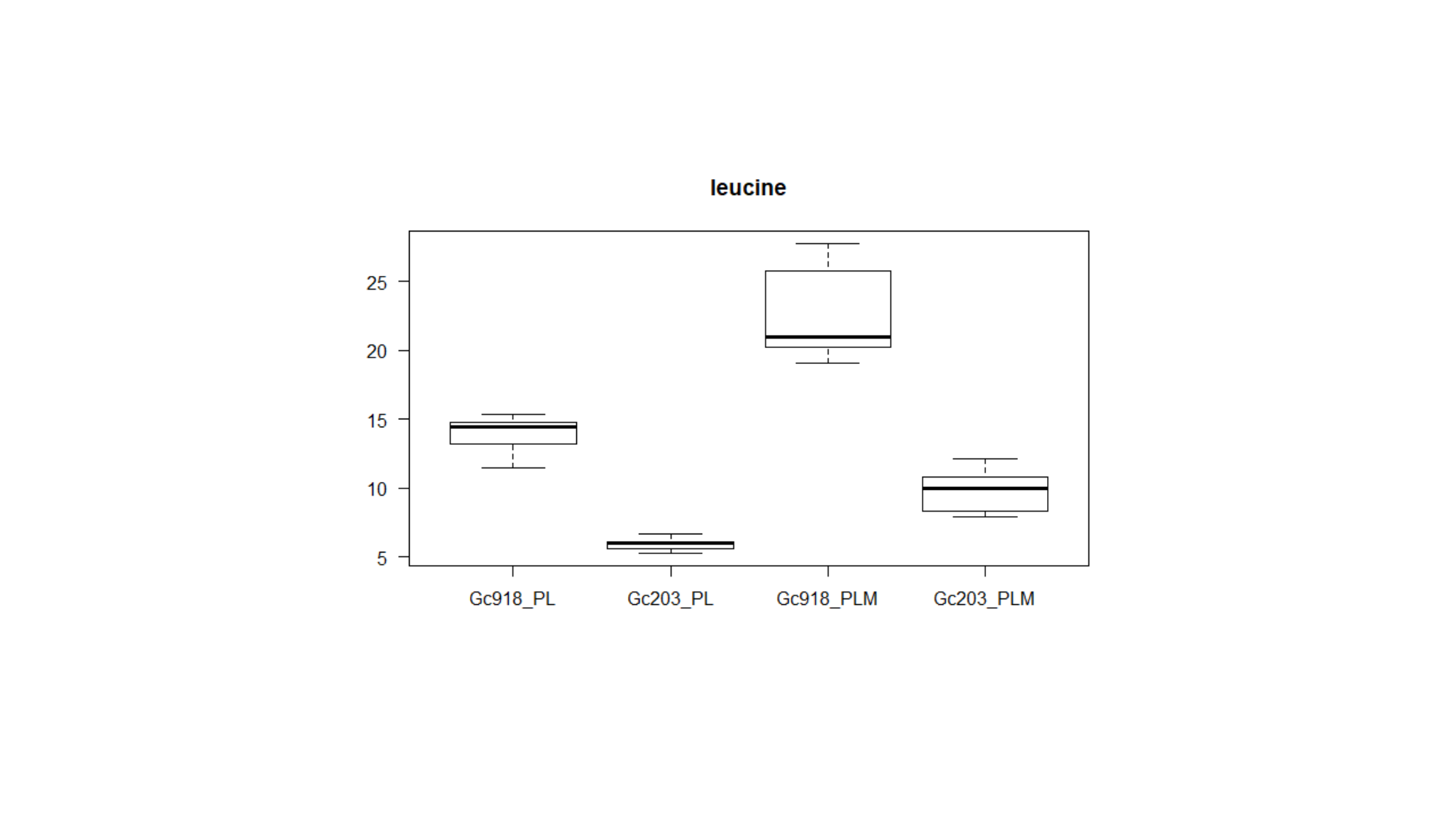

## Slide 47
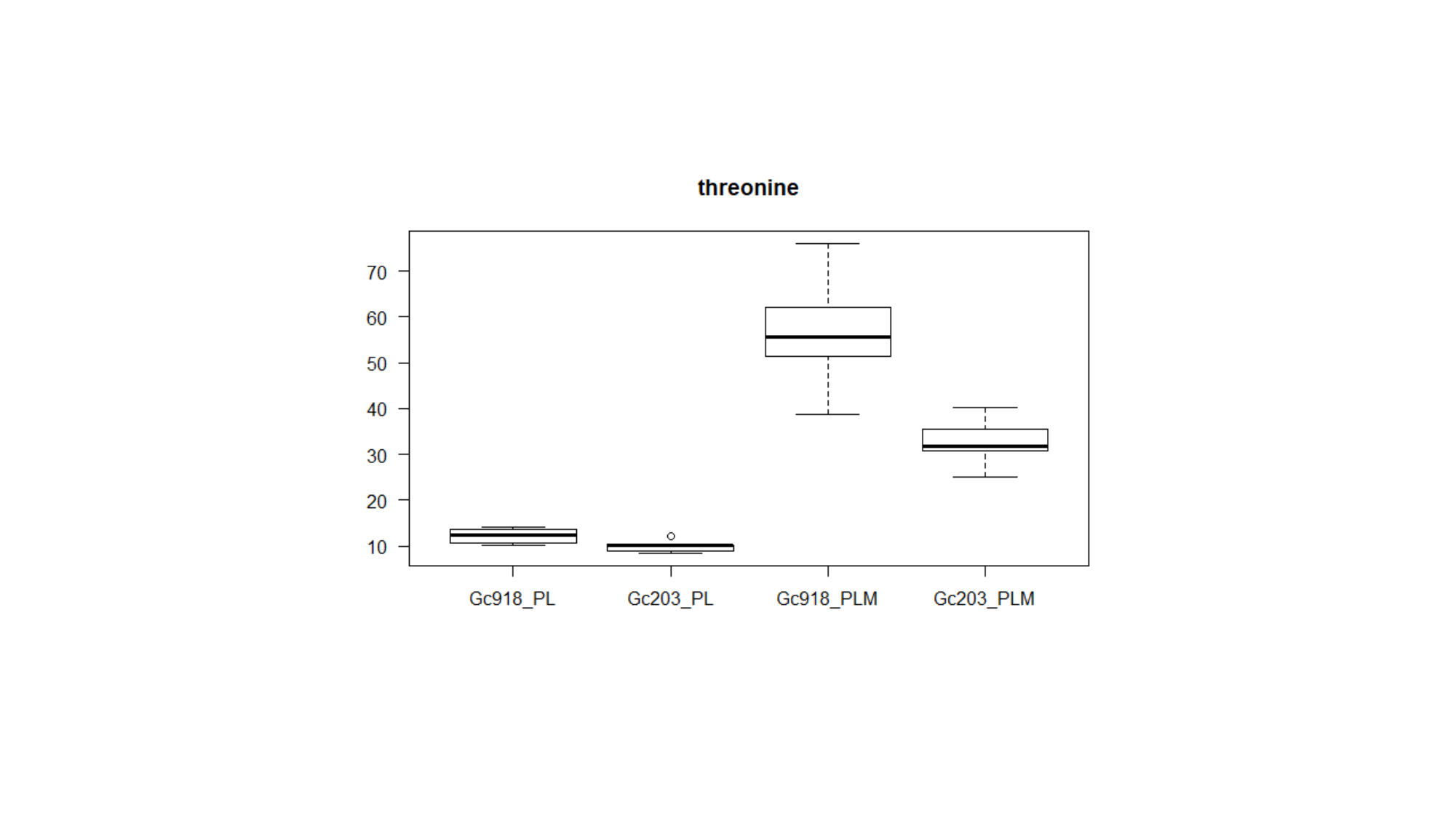

Supplement: Supplementary Data [file foy111_supplemental_files.zip › Supplementary_Boxplots.pptx]
