# Supplementary material for: Geotrichum candidum gene expression and metabolite accumulation inside the cells reflect the strain oxidative stress sensitivity and ability to produce flavour compounds
Source: FEMS Yeast Res. 2018 Oct 5;19(1):foy111. doi: 10.1093/femsyr/foy111 (PMC6211236; doi:10.1093/femsyr/foy111)
Supplement: Supplementary Data [file foy111_supplemental_files.zip › Supplementary_Calibration_table.docx]

| Calibration solution |  | Concentration (ng/µL) | | | |
| --- | --- | --- | --- | --- | --- |
| Retention time (min) | Compound | 0.05 | 0.5 | 2.5 | 5 |
| 1.55 | L-Cysteinesulfinic acid | 0.037 | 0.616 | 3.231 | 5.712 |
| 1.81 | Taurine | 0.099 | 0.86 | 3.078 | 5.226 |
| 1.88 | Hypotaurine | 0.066 | 0.758 | 3.386 | 5.942 |
| 2.53 | L-Aspartic acid | 0.088 | 0.747 | 3.5 | 5.914 |
| 2.67 | L-Serine | 0.108 | 0.628 | 2.717 | 4.559 |
| 2.81 | o-acetylserine | 0.059 | 0.683 | 2.98 | 5.001 |
| 2.87 | L-Glycine | 0.088 | 0.495 | 1.959 | 3.141 |
| 2.88 | L-Cysteine | 0.028 | 0.317 | 1.544 | 2.5 |
| 2.90 | L-Threonine | 0.088 | 0.744 | 3.169 | 5.132 |
| 3.05 | L-Glutamic acid | 0.093 | 0.862 | 3.592 | 6.132 |
| 3.18 | L-Alanine | 0.086 | 0.566 | 2.303 | 3.772 |
| 3.26 | L-Proline | 0.107 | 0.789 | 2.971 | 4.782 |
| 3.56 | DL-Cystathionine | 0.032 | 0.471 | 2.643 | 5.291 |
| 5.27 | DL-Homocysteine | 0.049 | 0.608 | 2.926 | 5.148 |
| 5.87 | L-Valine | 0.077 | 0.707 | 3.044 | 5.004 |
| 6.06 | L-Glutathione-reduced | 0.036 | 0.467 | 2.99 | 5.703 |
| 6.42 | Cysteinylglycine | 0.034 | 0.531 | 2.82 | 5.3 |
| 6.57 | Gamma-glutamylcysteine | 0.03 | 0.448 | 2.768 | 5.435 |
| 6.82 | L-Histidine | 0.079 | 0.782 | 3.585 | 6.613 |
| 8.09 | L-Methionine | 0.075 | 0.775 | 3.752 | 6.396 |
| 8.24 | L-Lysine | 0.058 | 0.624 | 3.319 | 6.112 |
| 9.94 | DL-Arginine | 0.085 | 0.822 | 3.977 | 7.593 |
| 10.11 | L-Tyrosine | 0.056 | 0.716 | 4.108 | 8.315 |
| 10.30 | Isoleucine | 0.094 | 0.782 | 3.456 | 5.725 |
| 10.46 | L-Leucine | 0.096 | 0.764 | 3.445 | 5.912 |
| 10.87 | L-Phenylalanine | 0.121 | 0.826 | 4.08 | 7.329 |
| 10.98 | S-adenosylmethionine | 0.032 | 0.427 | 2.708 | 5.394 |
