# Supplementary material for: Geotrichum candidum gene expression and metabolite accumulation inside the cells reflect the strain oxidative stress sensitivity and ability to produce flavour compounds
Source: FEMS Yeast Res. 2018 Oct 5;19(1):foy111. doi: 10.1093/femsyr/foy111 (PMC6211236; doi:10.1093/femsyr/foy111)
Supplement: Supplementary Data [file foy111_supplemental_files.zip › Supplementary_Primers_table.docx]

Primers list

|  | Forward primer (5’-3’) | Reverse primer (5’-3’) |
| --- | --- | --- |
| Reference genes |  |  |
| GECA01s10823g-ACT1  Actin | TCGATTCCGGTGATGGTGT | GTCGGTCAAGTCACGGC |
| GECA02s04817g-TFC1  RNA polymerase III transcription initiation factor | TTAAAGGAGCTGGAAGGCCC | CACCAAACTTGACAGCCGT |
| GECA04s07127g-TUB2  Beta-tubulin | AGCTCAATGGTGATCTGCG | GAGTTGGCAGCAAAGAGAGG |
| Aminotransferase genes |  |  |
| GECA08s02584g-BAT1  Mitochondrial  branched-chain amino acid aminotransferase | ACCATGATCCAGCTTATTTCCG | GTAGTCAGTGGCCTCGAGT |
| GECA01s10493g-BAT2  Cytosolic branched-chain  amino acid aminotransferase | ACTCTGTCAACGGTTGGT | TAGCACGATACGACGGCTAGAA |
| GECA03s05400g-ARO8-03  Aromatic aminotransferase | ATGAGGACTGGGACGTGATA | TGTCAAGGGCTTCAGGAATAATG |
| GECA27s00967g-ARO8-27  Aromatic aminotransferase | GAAAGTCCTACACTGAGAAGCG | CCTTAATGCCATGCTCGTAGA |
